# Forecasting the course of bipolar disorder using rest-activity rhythms: Protocol for a multi-study modelling project

**DOI:** 10.12688/wellcomeopenres.24189.1

**Published:** 2025-06-18

**Authors:** Greg Murray, Sandipan Ray, Richard Porter, Fatemeh Hadaeghi, Denny Meyer, Maree Choi, Sara Lapsley, Matthew Tennant, Bridgette Thwaites, Ly Nguyen, Hailey Tremain, Saibal Saha, Jan Scott

**Affiliations:** 1 Centre for Mental Health and Brain Sciences, Swinburne University of Technology, Melbourne, Victoria, Australia; 2 Department of Biotechnology, Indian Institute of Technology Hyderabad, Hyderabad, India; 3 Mental Health Clinical Research Unit, University of Otago, Christchurch, Otago, New Zealand; 4 Institute of Computational Neuroscience, Universitatsklinikum Hamburg-Eppendorf, Hamburg, Hamburg, Germany; 5 City University in Canada, Victoria Campus, Canada; 6 Psychological Medicine, Newcastle University, Newcastle, UK

**Keywords:** bipolar disorder, relapse, actigraphy, prospective, circadian, sleep, activity, molecular clock

## Abstract

**Background:**

Bipolar disorder (BD) is defined by complex, poorly understood mood dynamics. Our current inability to predict significant changes in mood, particularly episode relapses, is one of the most challenging features of BD for patients and clinicians. Research into sleep and circadian rhythm disruption suggests that relapse risk may be measurable in 24-hour rest-activity rhythms (RAR) collected by actigraphy. This protocol describes a multi-study project with two synergistic aims - to develop an algorithm of relapse risk based on RAR data and to advance mechanistic understanding of sleep and circadian rhythm features of RARs.

**Method:**

Three prospective studies will enrol adults living with BD I or II. Study 1 (Australia) will recruit a sample of *N* = 100 individuals with inter-episode BD who will participate in three contiguous 26-week monitoring epochs. Each epoch commences with 14 days of actigraphy, followed by follow-up interviews at 13 and 26 weeks. RAR predictors of time to first relapse will be explored using Cox’s survival analysis, Bayesian Network and Machine Learning analyses, and Hadaeghi’s Complex System model of BD. Study 2 (India) will recruit 25 individuals with BD and 25 matched healthy controls and replicate one of the 26-week epochs conducted in Study 1. Study 2 tests the replicability of models identified in Study 1 in a lower-middle-income country and investigates the association between amplitude of the RAR and amplitude of the diurnal rhythm in clock gene expression and associated metabolites. Study 3 (New Zealand) will collect actigraphy data from people experiencing a severe episode of BD depression ( *N* = 30) or mania ( *N* = 15), to model the prediction of treatment response and outcome of acute illness episodes.

**Discussion:**

The 5-year Tipping Point project uses novel methods and analyses to develop clinically critical inferences about the short-term course of BD. If replicated in future independent samples, the risk forecasting algorithm to be identified here could be translated into practice.

## Plain Language Summary

Even with the best available treatments, people living with bipolar disorder (BD) remain at risk of new episodes of depression and mania. One of the most challenging features of BD for patients and clinicians is that we cannot reliably predict when an individual might experience a further episode. This paper describes the protocol for an international project that aims to help with this problem. The Tipping Point project builds on research into, (i) sleep and body clock disturbances in BD, and (ii) ongoing monitoring in rest- activity and sleep-wake patterns in BD using an actigraph (a wrist-worn movement sensor similar to, but a bit more sophisticated than ‘consumer wearables’ such as Fitbits or sports watches). A collaborative team of researchers, clinicians, and lived experience experts have designed three studies which, together, will support development of an algorithm (based on rest-activity patterns) that forecasts relapse risk and response to treatment in BD. Of course, we appreciate our project will not answer all the questions people have about predicting BD outcomes, but we will share our data and our algorithm with other groups around the world to see if our findings can be replicated in different populations and geographical locations. A particular advantage of the project is that it offers a low-cost, non-invasive method that allows both clinicians and individuals with BD to spot early warning signs and symptoms of relapse and enables people to better manage their BD.

## Introduction

Bipolar disorder (BD) is a serious episodic mood disorder associated with significant morbidity and mortality ^1^. The psychological, familial, social, and clinical burden of BD is reflected in its economic cost ^2, 3^. In Australia, the excess health care costs of BD are about AU$7.3 billion per annum ^4^. It is estimated that three-quarters of this cost could be saved with improved management, particularly avoidance of hospitalisation via early warning of impending relapse ^4^. The present project takes up the challenge of improving forecasting of the short-term course (weeks and months) of BD, with the clinical aim of identifying an algorithm for BD relapse risk based on rest-activity rhythms (RAR). ^1^

The course of BD remains poorly understood, but we do know it is highly heterogeneous within and between individuals ^5^, and clinical characteristics (prior course, residual symptoms, etc.) are only modest predictors of relapse and recurrence ^6^. Emerging computational approaches have great potential to improve understanding the course of mood disorders, with models ranging from purely data-driven [e.g., 7, 8] to theoretically-informed models [e.g., 9, 10]. Here, the latter approach is prioritised, leveraging off rapidly expanding investment [e.g., 11] and knowledge about sleep and circadian rhythm disruption (SCRD) in psychopathology ^12, 13^.

The SCRD framework has unique strengths as an explanatory approach to BD. First, recent reviews highlight evidence for SCRD involvement in BD’s development and course, and specifically that SCRD variables may act as predictive biomarkers ^14– 16^. Secondly, an associated passive data collection methodology – 24-hour rest-activity rhythms (RAR) measured by actigraphy - supports investigation of chronobiology in the real world ^17^. Furthermore, actigraphy is non-invasive and inexpensive, and could potentially translate to an automated early warning tool ^18, 19^.

### Introduction to the circadian system and sleep

Evolution on earth has favoured organisms that anticipate, rather than react to, the planet’s dramatic daily shifts between light and dark. The mechanism of this ‘predictive homeostasis’ is endogenous biological clockwork, which coordinates internal physiology and environmental activity in most species ^20^. The functional endpoint of this clockwork is circadian (approximately 24-hour) rhythmicity in biological (e.g., core body temperature), behavioural (the activity-rest cycle), and mental (attention, affect) processes.

The molecular genetics of self-sustained circadian rhythmicity is well characterised at the cellular level in mammals ^21^. Within the master oscillator of the suprachiasmatic nucleus (SCN), self-sustained rhythmicity is generated by a primary intra-cell autoregulatory transcription-translation feedback loop (TTFL) involving the activators CLOCK and BMAL1, and their target genes Per1, Per2, Cry1 and Cry2, whose products form a negative-feedback repressor complex [additional stabilisation and auxiliary loops interlock with this core process, 22]. Similar cellular clocks operate in most cells, which receive inputs from the SCN to coordinate internal timing across tissues: Diurnal variation in clock gene expression in red blood cells, for example, has been used as an indicator of circadian amplitude and phase ^23, 24^.

 Despite their being collapsed in the acronym ‘SCRD’, it is important to think about circadian biology and sleep separately. From a circadian viewpoint, the sleep-wake cycle is the most visible circadian rhythm in humans ^25^. From the viewpoint of sleep *per se*, the circadian system is one of two primary drivers of sleep timing [the other being a homeostatic sleep drive, 26]. Parsing circadian from sleep processes in humans is a methodological challenge, traditionally addressed by sleep-disrupting laboratory protocols that are unsuitable for people with BD ^27^. As discussed below, contemporary translational research is increasingly using passive data collection and computational methods to study these processes in everyday life.

### Sleep and circadian rhythm disruption may be a predictive biomarker in BD

Sleep and circadian rhythm disturbances represent common symptoms of BD episodes but have also been shown to precede full-threshold episodes of BD [e.g., 28– 33]. Genetic variations in central clock genes have shown associations with mood disorder phenotypes, suggesting that knowledge of an individual’s circadian genotype may in the future be used to predict risk for mood disorder ^34^. For example, candidate gene association studies provide some support for associations between BD and common variants in *CLOCK* [the ClockΔ19-mutant mouse is the best-characterised animal model of BD, 35], and variants of *ARNTL* [see, for a review, 16].

A recent international expert review concluded that SCRD plays an important role in mental health, but causal pathways remain poorly understood ^11^. In mood disorders, several SCRD pathways have been proposed to be mechanistically important ^36– 38^. For example, decreased robustness of circadian rhythmicity - measurable in the attenuated amplitude of downstream rest-activity rhythms – may be an endophenotype of BD [e.g., 14, 16, 34, 39– 41].

### Actigraphy

Actigraphy was originally used to measure sleep patterns, where it has been shown to be reliable and valid ^42^, including amongst individuals with BD ^43, 44^. The last decade has seen rapidly increasing psychiatric interest in actigraphy due to: (i) recognition of activity disturbance as core to the BD phenotype ^45, 46^; (ii) its promotion by the US National Institute of Mental Health as a method for investigating arousal and regulatory systems ^47^; (iii) expanding research into digital phenotyping and passive sensing via wearables ^48– 51^, and (iv) emerging personalised models of psychopathology ^8, 52^.

Polysomnography (PSG) is the gold standard measure of sleep patterns and circadian markers of sleep-wake cycles. However, studies using actigraphy are overtaking PSG as the former offers an ecologically valid measurement of individual chronobiological functioning in everyday life ^53^. Over recent years, actigraphy research in BD has moved beyond the analysis of sleep parameters generated from the manufacturers’ software programmes (e.g., mean and standard deviation estimates of total sleep time, sleep onset latency, sleep efficiency, wake after sleep onset) to include estimation of RAR parameters that may be more critical for mental disorders such as stability, variability or predictability of 24-hour sleep-wake cycles and the regularity of timing of activity ^54^. Prominent in this work are three non-parametric RAR variables originally proposed by Van Someren and colleagues: Relative Amplitude, Intradaily Variability, and Interdaily Stability ^55^. Other work in BD has investigated actigraphy as a measure of chronotype [often delayed in BD compared to healthy controls, 53] and a range of sleep timing variables ^56^.

Actigraphy generates a high-resolution data stream (14 days’ recording generates 1.2 million data points), supporting a range of analyses, including but not limited to the macro-level sleep and circadian proxy variables introduced above [see, 53]. Actigraphic measurement figures prominently in computational models of mental health ^57, 58^, and mood disorders particularly [e.g., 59, 60]. Alongside survival analysis, the present project uses two complementary modelling approaches to advance prediction of BD course: machine learning (ML) models such as network analysis and complex systems analysis. Both are grounded in sleep and circadian science and a complex systems approach to psychopathology ^61^, and both have been championed by members of our team ^33, 62^.

***Actigraphic Measurement of the 24-h Rest-Activity Rhythm can Predict Course of BD.*** A substantial body of research has investigated *cross-sectional* associations between actigraphy-measured RAR variables and BD phenotypes. A recent systematic review identified 70 studies investigating RAR via actigraphy in BD samples ^17^. Consistent with the circadian robustness hypothesis (above), attenuated amplitude of RAR has been shown to associate with several BD phenotypes ^63– 65^. The largest study to date ^31^ found that a one-quintile (20%) decrease in relative amplitude (a measure of the average difference in most and least active periods over the course of a day compared with total activity) was associated with a small but significant increased risk of lifetime major depressive disorder (OR 1.06, 95% CI 1.04-1.08) and lifetime BD (OR 1.11, 1.03-1.20). ^2^

While such cross-sectional studies cannot determine the direction of causality, several groups have argued for the potential of RAR signals to generate a clinically-useful predictive algorithm of short-term course in mood disorders and BD specifically ^10, 17, 19, 42, 67– 69^. Prospective empirical investigations are limited to date, but an illustrative example is the study of Ferrand and colleagues, who sampled RAR with 14 days actigraphy data before prospectively tracking 69 euthymic individuals with BD for a median duration of 3.5 years ^70^. Some 65% of participants experienced a relapse or recurrence during follow-up. After adjusting for key clinical variables (age, sex, BD subtype, number of mood stabilizers, etc), time to episode onset was predicted by an actigraphy dimension comprising amplitude and variability/stability of circadian rhythms ( *p* = 0.009). The area under the curve (AUC) was improved from 0.64 for clinical predictors alone to 0.82 with the addition of actigraphy-derived IV ( *p* = 0.04).

An evidence-map review of prospective studies investigated actigraphy parameters as predictors of course, outcome, and treatment response in BD ^54^. Scott *et al.* concluded that actigraphy markers have the potential to predict future mental state, with the strongest individual predictors across studies being attenuated amplitude and delayed sleep onset. The authors note significant limitations in existing literature, including small sample sizes, varied endpoints, and an unsystematic approach to covariates and statistical modelling.

### The tipping point project

Existing actigraphy research provides intriguing preliminary evidence that RAR variables may predict the short-term course of BD. The Tipping Point project seeks to advance the field by pursuing two synergistic aims: (i) to advance the critical goal of developing a reliable predictive algorithm of relapse risk from actigraphy, and (ii) to improve mechanistic understanding of SCRD as measured by actigraphy.

As shown in Table 1, the project uses four work packages to address three objectives: 1) test an existing hypothesis of decreased circadian robustness as a predictor of relapse using Survival Analysis, 2) model causal pathways to predict relapse risk using ML models such as Network Analysis, and 3) model abrupt state transitions or tipping points using Hadaeghi’s Complex Systems Model. The protocol that follows introduces the integrative project as a whole - full methodological detail will be provided in three forthcoming study-level protocol papers.

**Table 1.** Project objectives and work packages.

Objective	WP1 Study 1 Inter-episode outpatient	WP2 Study 2 Inter-episode outpatient	WP3 Study 3 In-episode, predominantly inpatient	WP4 Synthesis and algorithm development
**Advance** **mechanistic** **understanding** **of SCRD as** **measured in RARs**	Hypothesis 1: Relative Amplitude from actigraphy is an independent predictor of time to relapse (Cox’s Survival Analysis Study 1 data)	Hypothesis 1: Relative Amplitude from actigraphy is a significant correlate of diurnal rhythms in circadian gene expression and metabolites from blood (Study 2 data) Hypothesis 2: Both Relative Amplitude from actigraphy and molecular diurnal rhythms are attenuated in BD versus matched health controls (Study 2 data)	-	Synthesise predictive findings from WP1-3; develop relapse risk algorithm
**Understand SCRD** **pathways to** **risk via machine** **learning models** **such as network** **analysis**	Dynamic Bayesian network analysis of actigraphy data (train and test on Study 1 data)	Dynamic Bayesian Network analysis of actigraphy data (replication in Study 2 LMIC cohort). Exploration of utility of other ensemble machine learning models (such as Boosting)	-	
**Understand** **risk in complex** **system terms as** **state transition**	Complex system analysis of actigraphy data informed by Hadaeghi model (train and test on Study 1 data)	Complex system analysis of actigraphy data (replication in Study 2 low/middle-income country cohort)	Complex system analysis of actigraphy data (extension to prediction of treatment response in Study 3)	

## Protocol

### Inclusion and exclusion criteria

Inclusion and exclusion criteria are minimal to increase the generalisability of project findings. In all studies, inclusion criteria are age 18–65 years and receiving treatment for a diagnosis of BD I or II. In all studies, participants must have sufficient language fluency (English in Studies 1 and 3; English, Hindi, Telegu, or Bengali in Study 2) to be able to provide informed consent and to complete self-report and interview-based assessments. Reason to believe that participation would interfere with treatment and/or recovery is an exclusion criterion in all three studies.

In Study 1 and 2, being in an episode at baseline is an exclusion criterion. Following common cut-offs [e.g., 71, 72] presence of an episode is defined as a score of > 12 on the Montgomery-Asberg Depression Rating Scale [MADRS, 73], or > 8 on the Young Mania Rating Scale [YMRS, 74]. In Study 3, by contrast, being in an episode of mania or bipolar depression is an inclusion criterion, as defined by a Clinical Global Impression Scale [CGI, 75] of 5 and above.

On duty of care grounds, clinical participants in Study 1 and 2 must be currently receiving treatment for BD from a medical practitioner and provide contact details and consent for the doctor to be contacted if required. Exclusion criteria in Study 1 and 2 are presence of a lifestyle (e.g., shiftwork) or physical problem (e.g., movement disorder) likely to impact RAR interpretation. Additional exclusion criterion in the outpatient Study 1 and 2 are the presence of psychotic symptoms, or active suicidality. Because of blood draws, a further exclusion criterion in Study 2 only is severe anaemia (Hb level < 7g/d). In Study 3, additional exclusion criteria are comorbid neurological disorder or sleep disorders impacting on sleep and movement (Narcolepsy, Restless Leg Syndrome), and current intoxication or withdrawal from illicit substances.

***Written and informed consent.*** In all studies, written informed consent will be obtained from each participant after they receive detailed explanations about the study procedure in the language best understood by them. Participants can withdraw from the study at any time but will not be able to withdraw data already provided. Participants will be invited to provide ‘extended’ consent so that data and analysis plans can be shared with appropriate research groups in this domain. All participants will be assigned a study ID to be used in place of the participant’s name on all assessment materials. The list linking IDs and names will be held under the same security conditions as study data (see below) and will be destroyed after data collection is complete. Participants are therefore potentially re-identifiable during data collection, but not identifiable after data collection is complete.

### Work packages

Three prospective monitoring studies are planned as three work packages. A fourth integrative work package will synthesise mechanistic and modelling findings from Studies 1 – 3 and develop the theoretically-informed relapse risk algorithm for testing in future independent samples.

**Figure 1.**
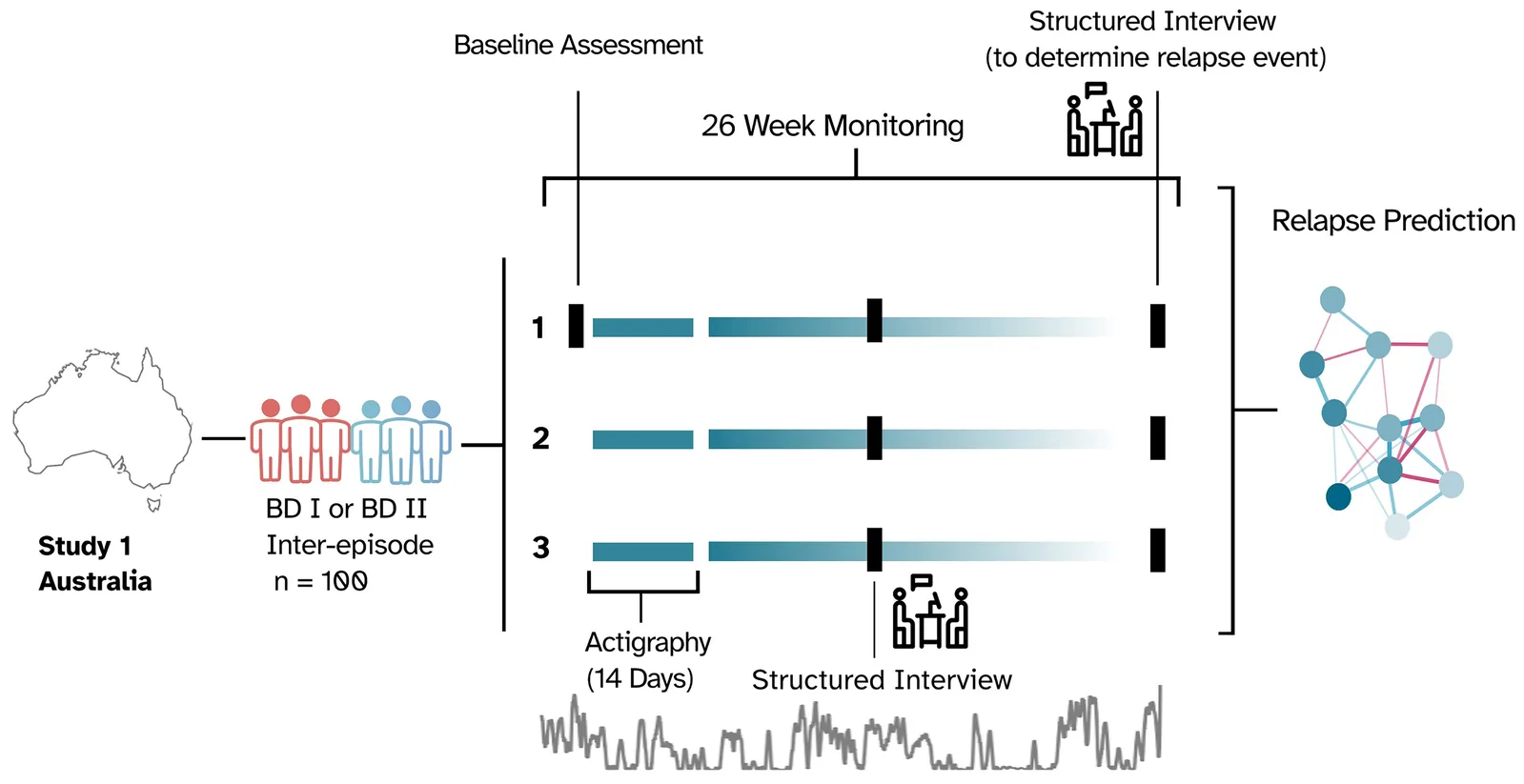
Study 1 design overview.

***Study 1: Predicting relapse in inter-episode BD from rest-activity rhythms.*** Study 1 (Australia) provides the primary 18-month dataset on which to conduct Survival Analysis, explore ML approaches such as Dynamic Bayesian Network (DBN) models in predicting the evolution of symptoms and full-threshold relapse, as well as train (75% of the sample) and test (unseen 25%) and novel approaches such as Hadaeghi’s complex system model. A total of *N* = 100 individuals will wear actigraphs for 14 days at the start of each of three consecutive 26-week monitoring epochs (predictive horizon = 26 weeks). In Study 1 and 2, time to relapse will be measured on the retrospective Longitudinal Interview Follow-up Evaluation (LIFE) interview ^76^ conducted at 13 and 26 weeks of each epoch ( Figure 1).

•   Hypothesis 1: It is hypothesised that Relative Amplitude will be an independent predictor of time to relapse using Cox’s Survival Analysis.

•   Research Question 1: Can an exploratory ML model of RAR variables (such as DBN) predict change in BD psychopathology and full-threshold relapses in a sample of individuals with BD I and II?

•   Research Question 2: Can a ML algorithm informed by Hadaeghi’s Complex System model of relapse, trained on statistical, time-frequency, and nonlinear dynamic features from RAR data collected from a cohort of *n* = 75 participants predict state transition (relapse) in an unseen subset of *n* = 25 participants?

•   Research Question 3: Do the three approaches to prediction (Survival Analysis, ML such as DBN, and complex system) generate convergent findings about relapse risk prediction from RAR?

**Figure 2.**
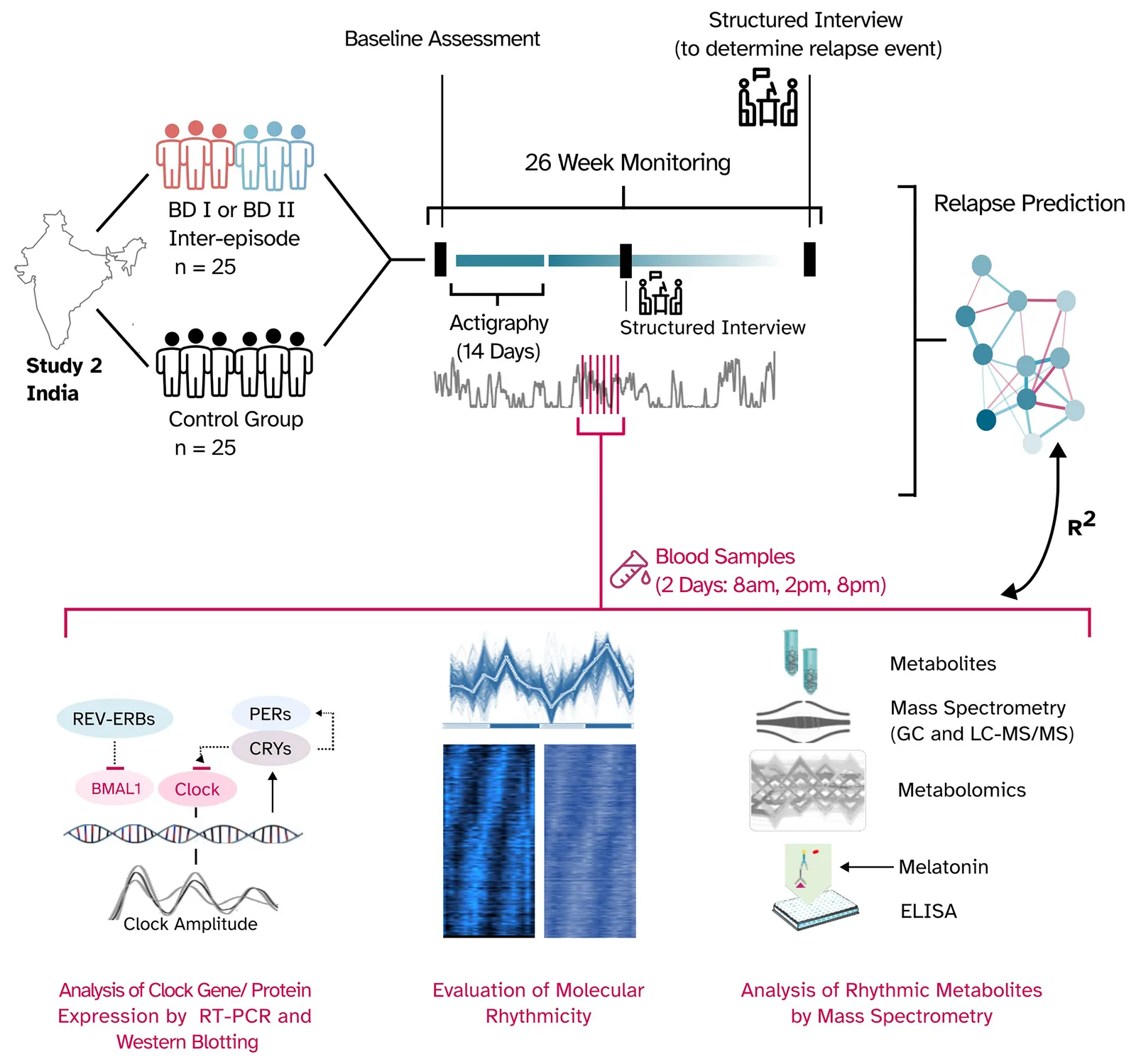
Study 2 design overview.

***Study 2: Exploring replicability of models in a lower-middle-income sample; testing molecular rhythmicity correlates of RAR variables.*** Study 2 (India) parallels Study 1’s prospective design amongst inter-episode individuals with BD, but with a single 26-week epoch ( Figure 2). Study 2 aims, first, to explore the replicability of Study 1’s Survival Analysis, DBN, and complex system models of relapse prediction in an out-of-distribution cohort from a lower-middle-income country (LMIC). Secondly, Study 2 will, (i) test the prediction that Relative Amplitude will correlate with diurnal amplitudes of gene expression and metabolites in blood, and (ii) test the hypothesis that both Relative Amplitude and these biological indicators of circadian robustness are attenuated in individuals with BD ( *n* = 25) relative to matched healthy controls ( *n* = 25).

•   Hypothesis 1: Amongst the complete sample ( *N* = 50), Relative Amplitude will correlate with the diurnal amplitude of both circadian gene expression and blood metabolites.

•   Hypothesis 2: Diurnal amplitude of circadian gene expression and blood metabolites, and Relative Amplitude will be attenuated in people with inter-episode BD ( *n* = 25) compared to age, gender, and employment-matched healthy controls ( *n* = 25).

•   Research Question 1: Does the Cox Survival Analysis model derived in Study 1 replicate amongst *n* = 25 BD participants in an LMIC cohort?

•   Research Question 2: Do any ML models enable examination of relapse prediction amongst *n* = 25 BD participants in a LMIC cohort?

•   Research Question 3 *:* Does the complex system model of state transition (relapse) prediction derived in Study 1 replicate amongst *n* = 25 BD participants in an LMIC cohort?

**Figure 3.**
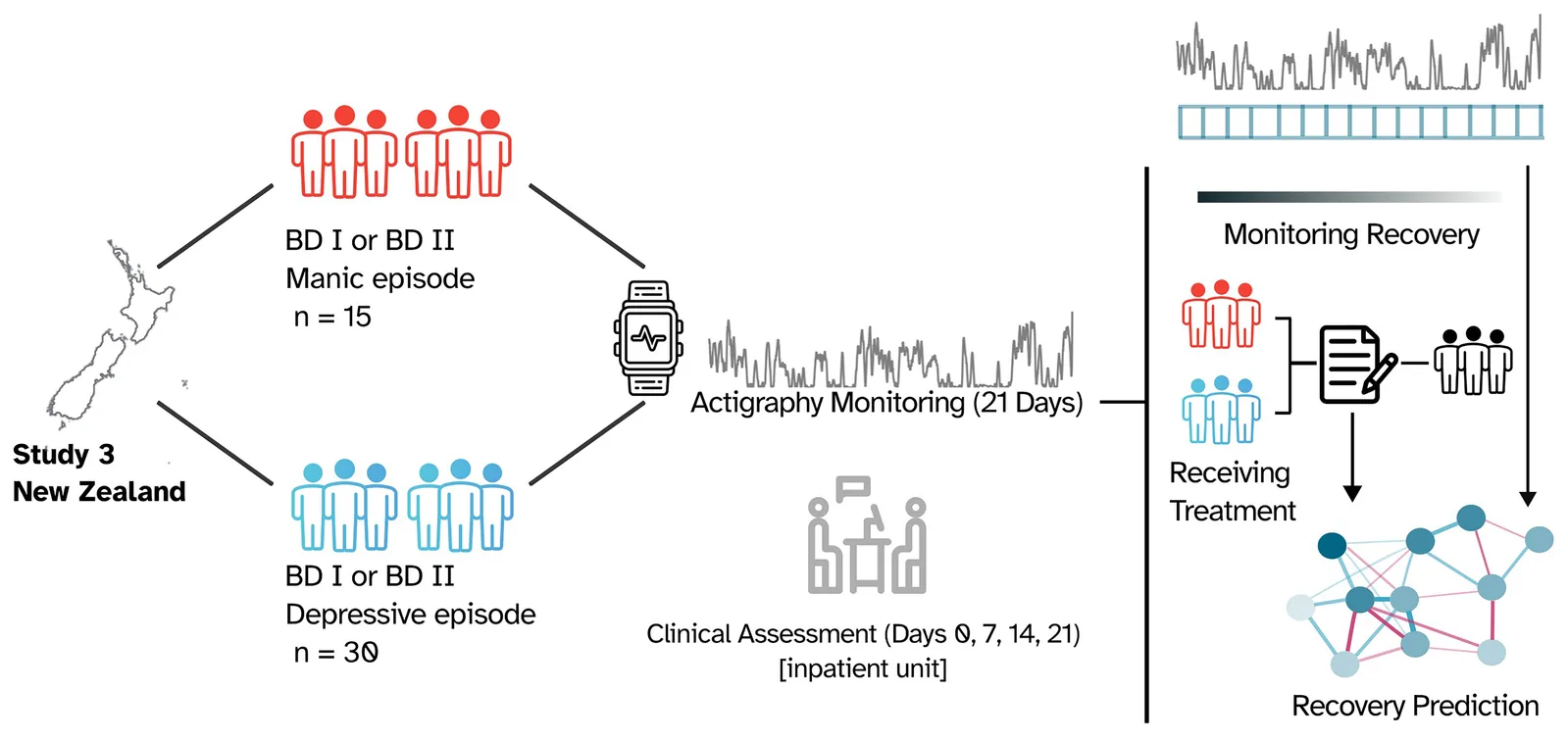
Study 3 design overview.

***Study 3: Exploring generalisability of the Machine Learning model of state transition from relapse prediction to treatment response.*** The primary aim of Study 3 (New Zealand) is to investigate the extension of the machine learning algorithm informed by Hadaeghi’s complex system model of relapse prediction (trained and tested on Study 1 data) to response to treatment, with both relapse (Study 1 outcome) and treatment response (Study 3 outcome) being understood as state transitions ^62^. A secondary aim of Study 3 is to explore the impact of baseline episode polarity (manic versus depressive) on predictive ML models of state transition. Participants will wear actigraphs for 21 days. The primary outcome will be measured at 8 weeks ( Figure 3).

Study 3 investigates the prediction of acute treatment response amongst people who are experiencing a severe episode of BD [an important clinical question in its own right, 77]. Actigraphy data will be collected from individuals with BD who are receiving clinical practice guideline-recommended care and treatment ^78^ for acute manic ( *n* = 15) or bipolar depressive ( *n* = 30) episodes (on an inpatient, outpatient, or hybrid basis). Study 3 participants will be patients of Te Whatu Ora - Waitaha Canterbury Mental Health Services.

- Research Question 1: How many additional samples ( *x*) from the *N* = 45 Study 3 sample are required to retrain the Study 1 complex systems prediction of state transition (prediction of relapse, *N* =75) to achieve acceptable prediction accuracy for a state transition complex system model predicting both relapse amongst inter-episode patients (from Study 1 data) and response to treatment amongst acutely ill patients (from Study 3 data)?
- Research Question 2: Can a complex systems model of state transition trained on *N* = 75 Study 1 samples and *x* (from Research Question 1) Study 3 samples predict state transition in an unseen data set of *N* = 25 Study 1 samples and N = (45 – *x*) samples from Study 3?
- Research Question 3: Is episode polarity at baseline in the Study 3 cohort ( *n* = 30 depression versus *n* = 15 manic) a moderator for the models developed in Research Question 1 and Research Question 2?

***Work package 4: Integrative data synthesis.*** Work package 4 will develop a relapse risk algorithm based on a synthesis of findings across studies. The specific analytic steps to be undertaken will necessarily depend on data patterns collected in the three studies: following advice from Wellcome Open (personal communication 21/6/24), a detailed statistical analysis plan will be published separately when this information is at hand, and prior to analyses being conducted. ^3^

Investigations in work package 4 may include new theoretical work to integrate network-based and complex systems-based approaches in this context [see for examples in other domains, 83– 86]. Two lines of research may be particularly relevant. First, originally developed for multivariate lesion-symptom mapping, multi-perturbation Shapley-value analysis [MSA, 87] has been extended in our work to investigate temporal causal connectivities in brain networks and artificial neural networks ^88, 89^. Briefly, MSA can rank the contribution of symptoms (nodes of the network) in relapse prediction for any given predictive model and any dynamical model used to mimic temporal changes in the symptoms. Second, our team employs excitable brain networks or excitable dynamics on the graph to examine the relationship between structural and functional connectivity in brain networks ^90– 92^.

### Measures

***Studies 1, 2 and 3.*** In all three studies, diagnostic inclusion and exclusion criteria will be confirmed on the Structured Clinical Interview for DSM-5, Research Version [SCID-5-RV, 93], as will the presence of common anxiety and substance use comorbid diagnoses. The Structured Clinical Interview for Sleep Disorders-Revised [SCISD-R, 94] will be used to assess the presence at baseline of sleep disorders (insomnia, hypersomnolence disorder) and circadian sleep-wake disorders (irregular sleep-wake disorder and non-24-hour sleep-wake type disorder).

***Study 1 and 2.*** People interested in participating will be invited to visit the study website (or if internet is not readily available, provided with written documentation where they can review study details), and then complete a consent form and answer an initial screening survey on demographic and inclusion/exclusion criteria (age, sex/gender, currently receiving medical treatment for a diagnosis of BD I or II, presence of lifestyle or physical condition impacting RAR). Those who pass the online screening questions will be invited to an appointment for a screening interview, conducted online in Study 1 and in person in Study 2. Interviewers will confirm that the potential participant is engaged in medical care for their BD and confirm consent. Participants who do not have a medical provider for their BD or who are experiencing acute mood episodes or suicidality will be provided with support and referral information and invited to recontact the researchers once treatment and symptom stability are in place. To minimize fraudulent engagement with these paid studies ^95^, participants’ identity will also be confirmed in Study 1 and 2 by presentation of a photo ID to the interviewer. In Study 1 and 2, the exclusion criterion of active suicidality is operationalized as a score of 3, 4, or 5 on the Columbia Suicide Severity Rating Scale [CSSRS, 96].

***Study 3.*** In Study 3, participants must be in an episode of mania or bipolar depression at baseline as assessed by a senior psychiatrist of the health service and have a CGI score of 5 and above (markedly ill).

### Primary outcome measures

***Study 1 and 2: Time to relapse.*** In Study 1 and 2, the time to relapse of any BD episode (manic, hypomanic, depressive) will be assessed using the LIFE. ^4^ The LIFE was developed to retrospectively assess the longitudinal course of psychiatric disorders over extended periods. Weekly data are elicited retrospectively, in an interview format. The method allows for the overall quantification of mood burden, through the interviewer determining ordinal weekly scores for severity of depression and mania. Patients are rated on a 1–6 scale, where 1—no symptoms, 2—residual symptoms, 3—partial remission, 4—does not meet DSM criteria but has major symptoms or impairment, 5—meets definite DSM criteria for an “episode”, and 6—fulfills definite criteria for an “episode” with the presence of either psychotic symptoms or extreme impairment in functioning. By determining whether syndromal symptoms last 2 weeks, relapse into a depressive episode, or a manic episode can be determined. The LIFE method has been used in a number of longer-term studies in BD to determine time to episode occurrence and overall mood morbidity [e.g., 98– 101]. Porter and colleagues have recently published an analysis of two studies validating the LIFE’s retrospective weekly estimates against concurrently rated clinician ratings on the MADRS and YMRS.

In Study 1 and 2, LIFE interviews will occur twice in each monitoring epoch (13 weeks, and 26 weeks, with 13 weeks’ retrospection window at each interview). The LIFE is commonly administered at 6-month intervals, and there is reason to believe that the 13-week intervals here will improve the reliability and resolution of our estimate of time to relapse ^71, 76^. Because of remote recruitment, the LIFE will be conducted by telehealth in Study 1. In Study 2, the LIFE will be conducted in person.

***Study 3: Treatment response.*** Response to treatment in Study 3 will be measured in weekly clinician assessment of current symptoms on the MADRS and YMRS (baseline, day 7, 14, 21), complemented by administration of the CGI. Administration of MADRS, YMRS, and CGI will be in person for inpatients, and via phone for outpatients. The primary outcome variable will be a 50% reduction in MADRS and YMRS scores at 8 weeks.

### Secondary outcome measures

Two types of secondary analysis will be conducted. First, if the number of observed relapses in Study 1 and 2 are insufficient to support reliable modelling, a secondary analysis will be conducted on the prediction of *symptom burden* in the follow-up window (three epochs in Study 1, one in Study 2). Weekly symptom burden is elicited retrospectively by the LIFE, on 6-point scales for weekly depression and mania (see above). Second, in all three studies modelling analyses will be re-conducted using the secondary outcome measure of self-reported Quality of Life (QoL), a patient-valued outcome. For the present project, QoL scores have the advantage of being a meaningful variable in both the inter-episode cohorts of Study 1 and 2, and the highly symptomatic cohort of Study 3. The BD-specific QoL.BD [short form, 102, 103] will be collected at 13- and 26-week follow-ups in Study 1 and 2, and weekly in Study 3.

### Predictors

***Sleep and circadian rhythm disruption predictors.*** First and foremost, predictors are objectively measured SCRD data from actigraphy (see above). To interpret actigraphy-based sleep variables, participants will also complete the Consensus Sleep Diary ^104^ across the 14 days of monitoring. The analyses described for Objective 1 (focusing on RA) will be repeated with Interdaily Stability or Intradaily Variability replacing Relative Amplitude if DBN or complex system analyses suggest these are important predictors.

Given the intimate involvement of environmental light in circadian entrainment ^105, 106^, predictive models may be improved if the actigraphy signal is augmented with continuous illuminance data [see, e.g., 107, 108]. Participants in Study 1 and 2 will wear *MiEye*, a lapel device measuring effective illuminance in the field, including melanopic and photopic illuminance continuously when out of bed ^109^.

***Clinical predictors.*** The present project assumes that objective SCRD data from actigraphy should be seen as augmenting, rather than competing with clinical variables in the prediction of BD course ^52, 109^. Informed by existing literature, five clinical predictors will be included in modelling of RAR-based variables in Study 1 and 2. Analyses will investigate the impact of, (i) BD subtype, (ii) number of past episodes (measured on the SCID-5-RV), (iii) current use of multiple mood stabilisers by self-report, (iv) residual depressive symptoms measured on the MADRS, and (v) self-reported medication adherence on the Medication Adherence Report Scale ^52^. Other variables will be included if a clear rationale can be provided, but a subject-to-variable ratio of 7 to 1 will be preferred.

### Modelling approaches

As noted above, it is not possible in this exploratory modelling project to present a detailed statistical analysis plan until the data to be analysed is clear. Here, we present a power analysis and the three planned analytic approaches.

***Power Analysis Study 1.*** Based on the effect size identified by Ferrand *et al.* (see above), a one standard deviation increase in the actigraphy measure increases the risk of relapse by 57% (Hazard Ratio = 1.57). Assuming a sample size of *N* = 100 participants, initially assuming zero drop-out and then 20% drop-out ( *N* = 80) we used simulation to calculate power estimates based on different Hazard Rate assumptions for a Cox Survival Analysis. First, a sample of *N* = 100 provides 90.6% power to detect a 57% increase in the relative risk of relapse while a sample size of *N* = 80 provides 75.6% power to detect such an increase. Second, a sample of *N* = 100 provides 83.0% power to detect a 49% increase in the relative risk of relapse while a sample size of *N* = 80 provides 83.8% power to detect a 65% increase in the relative risk of relapse. This suggests that even with a 20% dropout the study is nearly sufficiently powered to detect the expected relative risk of relapse (Hazard Ratio = 1.57) and well powered to detect a slightly higher relative risk of relapse (Hazard Ratio = 1.65).

***Cox Survival Analysis.*** Cox Survival Analysis will be employed with Elastic Net ^110^ to determine variables to include. To validate RA as a predictor of relapse, we will include RA in a random effects or frailty model of survival time to relapse, across the three epochs of Study 1 and the single epoch of Study 2.

***Machine Learning e.g., Network analysis.*** Network analysis is a variant of ML designed to illuminate the architecture of complex systems ^110^. In this project, we will use network analysis to model potential causal pathways amongst sleep and circadian variables in the prediction of relapse. The analysis generates a graphical representation of nodes (individual characteristics and variables) and edges (connections and links among nodes). Location of nodes and strength/valence of links between nodes represent the specific association between two nodes while controlling for all other variables.

Scott and colleagues recently undertook a pilot study in a sample of young people with emerging BD and produced a network model of RAR variables relative to each other and to phenotypic features of BD ^33^. This study found further support for the importance of robust circadian rhythmicity as measured in RA (see above). However, the authors highlight significant uncertainties in their modelling outcomes, concluding that “…whilst all research findings require replication and confirmation, we stress that this is particularly important in this emerging field of research” (p.225). The network analysis of Scott *et al.* was developed using cross-sectional data, while the present prospective project will permit exploration of how RAR variables change in relation to each other and to other BD symptoms over time. Dynamic Bayesian Network modelling (DBN) enables such an investigation [see, e.g., 111, 112] and its use will be explored here. In addition, we examine whether some other ML predictive models such as ensemble techniques like Boosting can be employed (these can be useful in unbalanced samples or where outcomes of interest are rarer).

***Complex systems analysis.*** A key clinical challenge to predicting the course of BD is its dramatic state changes, most commonly discussed in relation to switching ^113^. It has been proposed that switching and other nonlinear patterns in BD course are best understood from a complex systems viewpoint as clinically critical transitions in state space ^69, 114^.

The existing Hadaeghi Complex System model of BD is based on domain knowledge about interactions between the circadian pacemaker and the frontal cortex, and [in contrast to network analyses, 83] can account for endogenous and exogenous feedback loops ^62, 115^. The model has been demonstrated to replicate the erratic dynamics of BD that are sometimes exogenously driven (e.g., through intensive treatment). Critically for the present project, the model has been validated through secondary analysis of actigraphy data ^62^.

Informed by Hadaeghi’s model, the project aims to advance understanding and prediction of short-term course in BD by developing from actigraphy data a predictive model of clinically critical transitions in state space or tipping points. State transition is measured in Study 1 and 2 as the occurrence of relapse amongst inter-episode outpatients, and in Study 3 as the response to treatment amongst people experiencing an acute manic or bipolar depressive episode (Study 3).

### Procedures

***Project registration.*** The three-study project as detailed here was registered with Open Science Foundation on 24 ^th^ April 2025 ( https://doi.org/10.17605/OSF.IO/UABNJ). 

***Ethics.*** Ethics approval for Study 1 has been provided by Swinburne University of Technology Human Research Ethics Committee (SUHREC/2025/8127). Study 2 has received the biosafety approval from Institutional Biosafety Committee (IBSC) of Indian Institute of Technology Hyderabad and will now submit for approval to Institutional Ethics Committees of the Medical College Kolkata and Indian Institute of Technology Hyderabad (ITH/IEC/2025/01/2). Study 3 has ethical approval from the New Zealand Health and Disability Ethics Committee (HDEC, 2024 EXP 22105). Scientific findings will be reported according to the Strengthening the Reporting of OBservational studies in Epidemiology (STROBE) guidelines ^116^. The project sponsor is Swinburne University of Technology. 

***Risk management approach.*** In all studies, treatment as usual will continue throughout. All participants in Study 3 will be receiving active management under a specialist mental health team: Everyone receiving care under the specialist mental health services is required to have a treatment plan which includes a risk management plan. The nurse or research assistant conducting interviews will be aware of the individual’s existing treatment plan and will refer to this if required.

Risk management for Study 1 and 2 has been developed through our experience with other online interventions and websites for BD [e.g., 117– 120], and through consultation with our lived experience colleagues. Key principles of the approach include protection of autonomy where possible, explicit assignment of risk management to participants and their local networks, and advance planning for crises ^121^. Statements of informed consent highlight, (i) the project cannot and does not provide any therapy, treatment, support, or emergency management, (ii) the participant and their local networks of care remain central in their own safety and well-being throughout. The project landing page reiterates that no crisis support can be obtained through the site, but includes a ‘crisis’ tab, which provides suicide hotline phone numbers worldwide ( *unsuicide.wikispaces.com*).

To minimise the impact of any adverse events in Study 1 and 2, being under the ongoing care of a nominated medical practitioner is an inclusion criterion. Once participants consent, a letter/email will be sent to their practitioner noting that the patient has enrolled in the trial, that the researchers are not taking over their care, and that the provider will potentially be contacted should the researchers become aware of crisis or risk. At each self-report assessment, clinicians’ contact details will be updated if required and suicidality checked by re-administering the CSSRS. People are ineligible for participation if it is determined that their participation might compromise care. Participants who are at baseline (or become) ineligible due to active suicidality or other clinical concerns will be provided with support and referred back to their clinician, and invited to recontact researchers once treatment and symptom stability are in place.

In Study 1 and 2, all team members who have direct contact with participants will receive training concerning self-harm, suicidality, mania or depression symptom severity scores, and use of the C-SSRS interview to assess suicidality. A detailed manual will provide assessment probes, decision rules, referral resources, and contact information for use during emergency situations. Training will emphasize procedures for providing feedback in a clinically sensitive manner, and at all times patient welfare will be prioritised over collection of data. All staff will be trained to escalate to a clinical psychologist (Study 1) or psychiatrist (Study 2) if they learn of active suicidality.

***Data Security.*** Data for each study will be gathered using REDCap, a highly secure web-based research data collection system. REDCap provides several data security features, including secure passwords required for staff log-in, well-defined levels of data access based on a staff member’s role in the project, and data storage on the password-protected OneDrive of the lead organisation (Swinburne University of Technology). REDCap system design will be standardised across studies where possible to facilitate the merging of the data for work package 4. An additional separate REDCap system will be created for the genetic and metabolomic data of Study 2. Video contact with participants will be conducted by Swinburne Zoom accounts or Teams accounts; participants have the option to turn off their cameras during those meetings or to request telephone interviews in place of video calls (except for the initial confirmation of identity via photo ID).

Only members of the research team will have access to the data during data collection. However, appropriate data will also be released to the data safety and monitoring committee (DSMC). All data collected at each of the sites will be de-identified before transfer and uploaded as re-identifiable, password-protected files to the Swinburne OneDrive. Swinburne will hold a backup of all these data. Only members of the research team will be permitted to access these data. Data access and security arrangements will be detailed in the Participant Information Form and consented to by all participants.

A key deliverable of WP4 is a new multi-national dataset and data processing pipelines to be shared with future researchers. Data records and materials will be retained by Swinburne and archived for 20 years on Swinburne’s OneDrive. These data may be made available upon request on a case-by-case basis to external researchers consistent with the participants’ provision of extended consent. Code for the actigraphy processing pipeline will be shared through the project's GitHub repository server. Metabolomics data will be submitted to the Metabolights platform for sharing ( https://www.ebi.ac.uk/metabolights/index). This data repository follows globally accepted regulations.

To protect participant confidentiality, privacy standards that match or exceed those of all countries that the project recruits in will be maintained. Swinburne University of Technology will be the data custodian of data from all sites. Upon completion of the project and acceptance of its primary scientific outputs, de-identified data, data analysis scripts, statistical analysis plan, informed consent, publicly available (non-copyrighted) measures will be shared on the Open Science Foundation.

### Project governance

**Figure 4.**
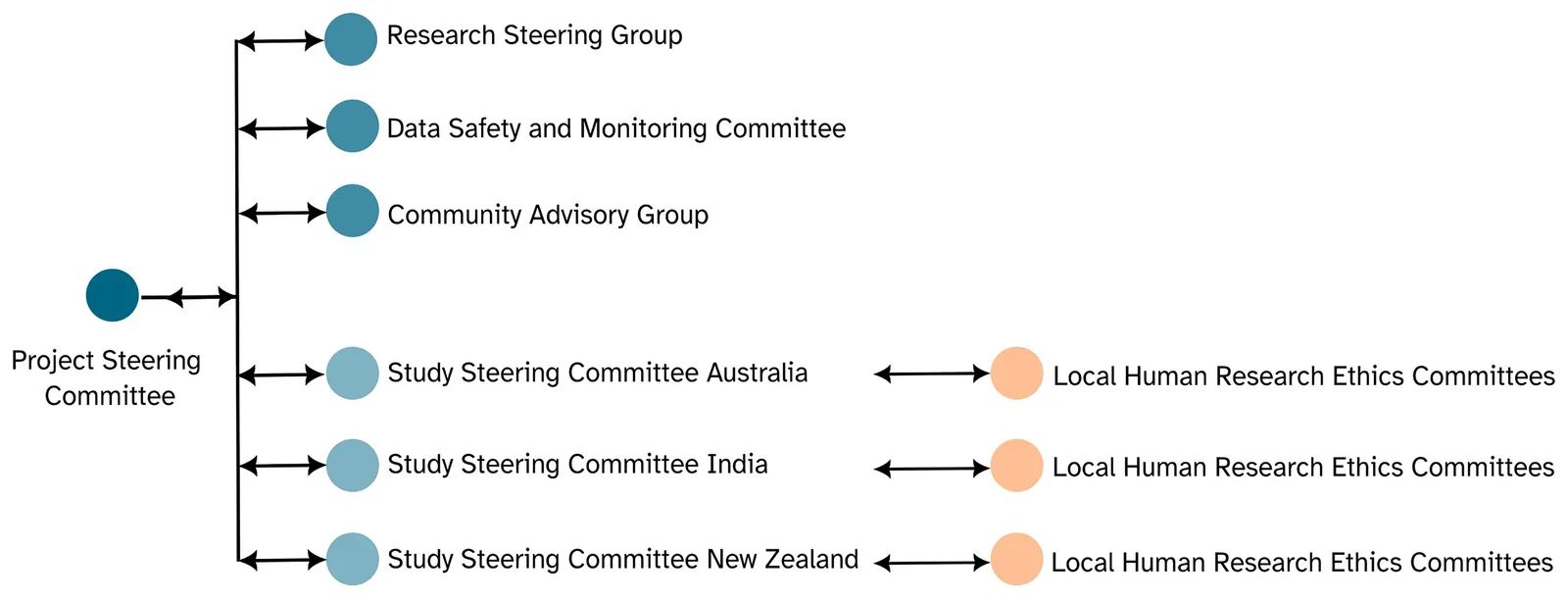
Project governance structure.

A Project Steering Committee will meet monthly to oversee project management, and scientific, ethical, legal, and societal aspects of the project (see Figure 4). Members of the Committee will include the Project Manager (Secretary), the individual study leads for the three empirical studies (WP1 = GM; WP2 = SR; WP3 = RP), leads of the data synthesis and modelling work (JS, FH, DM) and at least one LE collaborator. Local study steering committees will have day-to-day responsibility for the three empirical studies.

A research steering group (RSG) has been constituted to keep the funder informed about project progress. Membership of the RSG includes the Principal Investigator (GM); at least one independent expert adviser with experience relevant to the Project appointed by Wellcome; one representative of the Swinburne’s technology transfer office; and at least one representative or nominee of Wellcome. The RSG meets annually and receives project reports every 6 months.

The DSMC will be led by an independent chair. The committee charter empowers the committee to recommend removing participants from a study or recommending that a study be modified or stopped. Membership includes an independent statistician, and an early career researcher with experience in circadian rhythms, mood disorders, and prospective study designs. The committee will review all aspects of the protocol before the project begins and be informed of the progress of the three empirical studies, including enrolment, attrition, outcome variables, adverse events, and planned protocol changes. The DSMC will hold regular meetings to review individual safety reports, aggregate event rates, data quality and security, protocol adherence, participant recruitment and retention rates (including participant burden issues). The DSMC will review participants’ formal outcome assessments and any adverse events. Serious adverse events (participant death, hospital admissions, episodes of crisis assessment team treatment, attempted suicide, violent incidents leading to police involvement) will be recorded systematically. The DSMC will review data of the first 5 baseline assessments for Study 1, 2, and 3 to exclude systematic adverse events associated with participation. The DSMC will meet twice during the first year of data collection, and annually thereafter or as required. Reports of the DSMC will be forwarded to the RSG (and where required, local HRECs).

Finally, a community advisory group (CAG) will be developed to provide input to the Project Steering Committee. Chaired by one of our LE collaborators (SL), the CAG will meet three times throughout the project with provisional agendas set for each session (e.g., Yr 1: project protocol, participant burden of assessments and recruitment, Yr 3: recruitment and retention, Yr 5: interpreting and disseminating findings, next steps, participants’ views of the findings). The Project Steering Committee will provide feedback to the CAG on how their input did or did not affect project plans and why.

### Timelines and deliverables

All four work packages will be conducted in parallel (see Table 2). Careful attention has been paid to staffing of the work packages to ensure feasible workloads across researchers and project staff.

**Table 2.** Gantt chart of project activities.

Task	Year 1				Year 2				Year 3				Year 4				Year 5			
	1	2	3	4	1	2	3	4	1	2	3	4	1	2	3	4	1	2	3	4
**Project oversight**																				
Governance structures																				
Inter-institution contracts																				
Project-level protocol paper																				
Recruit project manager																				
**Work Package 1 Australia**																				
Ethics approval																				
Recruit and train study staff																				
Build local data base																				
Recruitment																				
Data collection																				
Local data cleaning and analysis																				
**Work Package 2 India**																				
Ethics approval																				
Build local data base																				
Recruitment																				
Data collection																				
Local data cleaning and analysis																				
**Work Package 3 New Zealand**																				
Ethics approval																				
Build local data base																				
Recruitment																				
Data collection																				
Local data cleaning and analysis																				
**Work Package 4**																				
Theoretical work																				
Data pipeline development																				
Coordinate data transfer to Swinburne																				
Cross-validation of findings																				
Data synthesis																				
Model-building																				
**Dissemination**																				
Draft publications																				
Prepare conference submissions																				
Report findings to participants, seek feedback																				

*Note: Project formal commencement date 14th July, 2024*

## Discussion

As a relatively large 5-year multi-study international project with a strong theoretical rationale, the Tipping Point project is the obvious next step in a stream of research investigating mood disorder biomarkers via actigraphy. The project extends upon previous prospective studies by including larger, more representative samples of individuals with BD and purposefully applying three complementary approaches to modelling RAR.

The project has a number of strengths. It is highly interdisciplinary, bringing together psychology, psychiatry, statistics, computational biology, molecular biology, and behavioural analyses. Our long-standing LE collaborators were influential in developing the grant application, the present protocol, and will remain active in data collection, governance, and translation of findings. Growing from our work in participatory research [e.g., 122– 124], an assumption of the project is that participants are also collaborators in, rather than subjects of, the research: they will be properly remunerated for their time and consulted on project findings to inform next steps. A unique strength of the project is the collection and comparison of data from Australian and Indian samples: whilst the latter sample is of modest size, the project is an important first attempt to see if modelling findings replicate beyond high-income countries ^125^.

Four embedded PhD projects will train the next generation of researchers in this area. Projects will generate important contextual knowledge about lived experience perspectives on prospective monitoring of relapse by actigraphy (embedded in Study 1), optimising measurement of circadian rhythmicity by blood-based molecular measurement (Study 2), understanding consent to participate in research amongst individuals currently unwell with mania (Study 3), and theoretical considerations in integrating network and complex systems predictive models using ML (work package 4).

The most important limitation of the project is the small number of data points for machine learning analyses ^126^. For example, the person-days of Study 1 ( *N* = 14 days X 3 epochs X 100 participants = up to 4,200 actigraph days; *N* = 7 days X 26 weeks X 3 epochs X 100 participants = up to 54,600 follow-up days) make it the largest prospective investigation of actigraphic predictors of BD course. However, the total number of participants ( *N* = 100 at baseline) for modelling relapse events in Study 1 is small: A commonly cited prospective study estimates individuals with BD I experience 0.4 new episodes per annum on average ^127^, so we estimate only some 60 relapses will be observed. Relatively small samples in all three studies also mean we are collapsing different polarities in primary analyses and can only statistically explore potentially important covariates including BD diagnosis, co-morbidities, and medication regimen. Replication of the project’s findings in unseen data sets is therefore absolutely critical, and we are in discussion with other teams worldwide for data sharing to support these tests.

The project’s findings will have several potential implications. First and foremost, if a predictive algorithm of relapse risk can be identified and subsequently replicated in independent samples, the project will have made a significant step towards an automated forecasting tool for BD [as being trialled for people living with epilepsy, 128]. Beyond providing early warning advice, such an indicator may play a more positive role in BD management, by informing treatment decisions. For example, such a tool could play a part in deciding who may not require ‘infinite maintenance treatment’ for their BD ^129^.

Secondly, the project will generate an entirely novel network model of the relationship between activity, sleep, and circadian parameters as predictors of relapse in BD. As argued in a recent expert report, mechanistic understanding of SCRD in mental health is lacking ^11^. The actigraphy signal alone is not a direct measure of biological mechanisms, but the addition of ML, network analysis, and the triangulation provided by three studies (including a traditional biosignature design in Study 2) will support parsing out these interwoven signals ^53^. Similarly, the investigation of non-linear dynamic models of predicting state transitions is likely to advance understanding of BD from a complex systems perspective.

A further deliverable is a large open science database and analysis pipelines, to be shared with future researchers who wish to apply different statistical and analytic techniques. In close collaboration with other research groups funded by the Wellcome Trust SCRD funding scheme, the research team will ensure that the multi-national dataset and processing pipelines meet the FAIR criteria ^130^.

## Conclusion

The present paper introduces the rationale for, and four work packages of, a 5-year international project designed to incrementally advance understanding of SCRD as a predictor of mood dynamics in BD.

## Ethical considerations

Australian and Indian participants will provide written and informed consent at study sign-up via the online sign-up form. Participants will then reconfirm their written and informed consent with the research assistants prior to the commencement of the baseline assessment. New Zealand participants will provide written and informed consent prior to the commencement of the study interview after their capacity to provide consent has been confirmed by an independent senior clinician. Participants will be encouraged to use supported decision making with a trusted family member or friend and will have their consent revisited during and after their time in the study, acknowledging that these participants are experiencing an acute mood state which is dynamic in nature may impact their decision to take part. Ethical approval for Study 1 was provided by Swinburne University of Technology Human Research Ethics Committee on 11/11/2024 (SUHREC/2025/8127). Study 2 has received the biosafety approval from Institutional Biosafety Committee (IBSC) of Indian Institute of Technology Hyderabad on 30/12/2024 (ITH/IEC/2025/01/2). Study 3 has ethical approval (2024 EXP 22105), from the New Zealand Health and Disability Ethics Committee (HDEC).

## References

[1] Whiteford HA Degenhardt L Rehm J : Global Burden of Disease attributable to mental and substance use disorders: findings from the Global Burden of Disease Study 2010. *Lancet.* 2013;382(9904):1575–1586. 10.1016/S0140-6736(13)61611-6 23993280

[2] Bergeson JG Kalsekar I Jing Y : Medical care costs and hospitalization in patients with bipolar disorder treated with atypical antipsychotics. *Am Health Drug Benefits.* 2012;5(6):379–386. 24991334 PMC4031693

[3] Chen M Fitzgerald HM Madera JJ : Functional outcome assessment in bipolar disorder: a systematic literature review. *Bipolar Disord.* 2019;21(3):194–214. 10.1111/bdi.12775 30887632 PMC6593429

[4] KPMG: Paving the way for mental health: the economics of optimal pathways to care. 2014. Reference Source

[5] Duffy A Grof P : Longitudinal studies of bipolar patients and their families: translating findings to advance individualized risk prediction, treatment and research. *Int J Bipolar Disord.* 2024;12(1): 12. 10.1186/s40345-024-00333-y 38609722 PMC11014837

[6] Etain B Bellivier F Olié E : Clinical predictors of recurrences in bipolar disorders type 1 and 2: a FACE-BD longitudinal study. *J Psychiatr Res.* 2021;134:129–137. 10.1016/j.jpsychires.2020.12.041 33385631

[7] Colombo F Calesella F Mazza MG : Machine learning approaches for prediction of bipolar disorder based on biological, clinical and neuropsychological markers: a systematic review and meta-analysis. *Neurosci Biobehav Rev.* 2022;135: 104552. 10.1016/j.neubiorev.2022.104552 35120970

[8] Lipschitz JM Lin S Saghafian S : Digital phenotyping in bipolar disorder: using longitudinal Fitbit data and personalized machine learning to predict mood symptomatology. *Acta Psychiatr Scand.* 2025;151(3):434–447. 10.1111/acps.13765 39397313 PMC13552231

[9] Esaki Y Obayashi K Saeki K : Association between circadian activity rhythms and mood episode relapse in bipolar disorder: a 12-month prospective cohort study. *Transl Psychiatry.* 2021;11(1): 525. 10.1038/s41398-021-01652-9 34645802 PMC8514471

[10] Fried EI Proppert RKK Rieble CL : Building an early warning system for depression: rationale, objectives, and methods of the WARN-D study. *Clin Psychol Eur.* 2023;5(3): e10075. 10.32872/cpe.10075 38356901 PMC10863640

[11] Wellcome Trust: Sleep, circadian rhythms and mental health.Wellcome Trust: London, UK, 2022;47. Reference Source

[12] Hickie IB Crouse JJ : Sleep and circadian rhythm disturbances: plausible pathways to major mental disorders? *World Psychiatry.* 2024;23(1):150–151. 10.1002/wps.21154 38214638 PMC10786001

[13] Meyer N Lok R Schmidt C : The sleep-circadian interface: a window into mental disorders. *Proc Natl Acad Sci U S A.* 2024;121(9): e2214756121. 10.1073/pnas.2214756121 38394243 PMC10907245

[14] Scott J Etain B Miklowitz D : A systematic review and meta-analysis of sleep and circadian rhythms disturbances in individuals at high-risk of developing or with early onset of bipolar disorders. *Neurosci Biobehav Rev.* 2022;135: 104585. 10.1016/j.neubiorev.2022.104585 35182537 PMC8957543

[15] Scott J Hennion V Meyrel M : An ecological study of objective rest-activity markers of lithium response in bipolar-I-disorder. *Psychol Med.* 2022;52(12):2281–2289. 10.1017/S0033291720004171 33183364

[16] McCarthy MJ Gottlieb JF Gonzalez R : Neurobiological and behavioral mechanisms of circadian rhythm disruption in bipolar disorder: a critical multi-disciplinary literature review and agenda for future research from the ISBD task force on chronobiology. *Bipolar Disord.* 2022;24(3):232–263. 10.1111/bdi.13165 34850507 PMC9149148

[17] Panchal P de Queiroz Campos G Goldman DA : Toward a digital future in bipolar disorder assessment: a systematic review of disruptions in the rest-activity cycle as measured by actigraphy. *Front Psychiatry.* 2022;13: 780726. 10.3389/fpsyt.2022.780726 35677875 PMC9167949

[18] Bullock B Judd FK Murray G : Using actigraphy to monitor sleep-wake patterns in bipolar disorder - A case study. *Clinical Psychology Forum.* 2014; (253):37–41. 10.53841/bpscpf.2014.1.253.37

[19] Jakobsen P Côté-Allard U Riegler MA : Early warning signals observed in motor activity preceding mood state change in bipolar disorder. *Bipolar Disord.* 2024;26(5):468–478. 10.1111/bdi.13430 38639725

[20] Foster RG : Sleep, circadian rhythms and health. *Interface Focus.* 2020;10(3): 20190098. 10.1098/rsfs.2019.0098 32382406 PMC7202392

[21] Jagannath A Taylor L Wakaf Z : The genetics of circadian rhythms, sleep and health. *Hum Mol Genet.* 2017;26(R2):R128–R138. 10.1093/hmg/ddx240 28977444 PMC5886477

[22] Takahashi JS : Transcriptional architecture of the mammalian circadian clock. *Nat Rev Genet.* 2017;18(3):164–179. 10.1038/nrg.2016.150 27990019 PMC5501165

[23] Laloum D Robinson-Rechavi M : Methods detecting rhythmic gene expression are biologically relevant only for strong signal. *PLoS Comput Biol.* 2020;16(3): e1007666. 10.1371/journal.pcbi.1007666 32182235 PMC7100990

[24] Yang MY Yang WC Lin PM : Altered expression of circadian clock genes in human chronic myeloid leukemia. *J Biol Rhythms.* 2011;26(2):136–48. 10.1177/0748730410395527 21454294

[25] Webb WB : Sleep as a biological rhythm: a historical review. *Sleep.* 1994;17(2):188–194. 10.1093/sleep/17.2.188 8036374

[26] Borbély AA Daan S Wirz-Justice A : The two-process model of sleep regulation: a reappraisal. *J Sleep Res.* 2016;25(2):131–43. 10.1111/jsr.12371 26762182

[27] Yousefzadehfard Y Wechsler B DeLorenzo C : Human circadian rhythm studies: practical guidelines for inclusion/exclusion criteria and protocol. *Neurobiol Sleep Circadian Rhythms.* 2022;13: 100080. 10.1016/j.nbscr.2022.100080 35989718 PMC9382328

[28] American Psychiatric Association: Text revision of the diagnostic and statistical manual of mental disorders. fifth edition: DSM-5-TR. 5th ed. Washington, DC: Author,2022;1120. Reference Source

[29] Gottlieb JF Benedetti F Geoffroy PA : The chronotherapeutic treatment of Bipolar Disorders: a systematic review and practice recommendations from the ISBD task force on chronotherapy and chronobiology. *Bipolar Disord.* 2019;21(8):741–773. 10.1111/bdi.12847 31609530

[30] Logan RW McClung CA : Rhythms of life: circadian disruption and brain disorders across the lifespan. *Nat Rev Neurosci.* 2019;20(1):49–65. 10.1038/s41583-018-0088-y 30459365 PMC6338075

[31] Lyall LM Wyse CA Graham N : Association of disrupted circadian rhythmicity with mood disorders, subjective wellbeing, and cognitive function: a cross-sectional study of 91 105 participants from the UK Biobank. *Lancet Psychiatry.* 2018;5(6):507–514. 10.1016/S2215-0366(18)30139-1 29776774

[32] McCarthy MJ : Missing a beat: assessment of circadian rhythm abnormalities in Bipolar Disorder in the genomic era. *Psychiatr Genet.* 2019;29(2):29–36. 10.1097/YPG.0000000000000215 30516584

[33] Scott J Etain B Grierson A : A network analysis of rest-activity rhythms in young people with emerging Bipolar Disorders. *J Affect Disord.* 2022;305:220–226. 10.1016/j.jad.2022.03.007 35288205

[34] Bhatnagar A Murray G Ray S : Circadian biology to advance therapeutics for mood disorders. *Trends Pharmacol Sci.* 2023;44(10):689–704. 10.1016/j.tips.2023.07.008 37648611

[35] Vitaterna MH King DP Chang AM : Mutagenesis and mapping of a mouse gene, *clock*, essential for circadian behavior. *Science.* 1994;264(5159):719–25. 10.1126/science.8171325 8171325 PMC3839659

[36] Hühne A Welsh DK Landgraf D : Prospects for circadian treatment of mood disorders. *Ann Med.* 2018;50(8):637–654. 10.1080/07853890.2018.1530449 30265156

[37] McClung CA : How might circadian rhythms control mood? Let me count the ways. *Biol Psychiatry.* 2013;74(4):242–9. 10.1016/j.biopsych.2013.02.019 23558300 PMC3725187

[38] Roguski A Ritter P Smith DJ : Sensitivity to light in bipolar disorder: implications for research and clinical practice. * Br J Psychiatry.* 2024;224(5):143–146. 10.1192/bjp.2023.150 38174418 PMC7615859

[39] Bullock B Murray G : Reduced amplitude of the 24 hour activity rhythm: a biomarker of vulnerability to bipolar disorder? *Clin Psychol Sci.* 2014;2(1):86–96. 10.1177/2167702613490158

[40] Guglielmo R Miskowiak KW Hasler G : Evaluating endophenotypes for bipolar disorder. *Int J Bipolar Disord.* 2021;9(1): 17. 10.1186/s40345-021-00220-w 34046710 PMC8160068

[41] Hwang JY Choi JW Kang SG : Comparison of the Effects of Quetiapine XR and Lithium Monotherapy on Actigraphy-Measured Circadian Parameters in Patients With Bipolar II Depression. *J Clin Psychopharmacol.* 2017;37(3):351–354. 10.1097/JCP.0000000000000699 28328790

[42] Patterson MR Nunes AAS Gerstel D : 40 years of actigraphy in sleep medicine and current state of the art algorithms. *NPJ Digit Med.* 2023;6(1): 51. 10.1038/s41746-023-00802-1 36964203 PMC10039037

[43] Kaplan KA Talbot LS Gruber J : Evaluating sleep in bipolar disorder: comparison between actigraphy, polysomnography, and sleep diary. *Bipolar Disord.* 2012;14(8):870–9. 10.1111/bdi.12021 23167935 PMC3549461

[44] Ng TH Chung KF Ho FYY : Sleep–wake disturbance in interepisode bipolar disorder and high-risk individuals: a systematic review and meta-analysis. *Sleep Med Rev.* 2015;20:46–58. 10.1016/j.smrv.2014.06.006 25060968

[45] American Psychiatric Association: Diagnostic and statistical manual of mental disorders: DSM-5.5th ed. Washington, DC: Author.2013;947. 10.1176/appi.books.9780890425596

[46] Scott J Murray G Henry C : Activation in bipolar disorders: a systematic review. *JAMA Psychiatry.* 2017;74(2):189–196. 10.1001/jamapsychiatry.2016.3459 28002572

[47] National Advisory Mental Health Council Workgroup on Tasks and Measures for Research Domain Criteria: Behavioral Assessment Methods for RDoC Constructs.National Institutes of Health: Bethesda, MD, USA,2016. Reference Source

[48] de Zambotti M Goldstein C Cook J : State of the science and recommendations for using wearable technology in sleep and circadian research. *Sleep.* 2024;47(4): zsad325. 10.1093/sleep/zsad325 38149978

[49] Hauser TU Skvortsova V De Choudhury M : The promise of a model-based psychiatry: building computational models of mental ill health. *Lancet Digit Health.* 2022;4(11):e816–e828. 10.1016/S2589-7500(22)00152-2 36229345 PMC9627546

[50] Lee MP Kim DW Fang Y : The real-world association between digital markers of circadian disruption and mental health risks. *NPJ Digit Med.* 2024;7(1): 355. 10.1038/s41746-024-01348-6 39639100 PMC11621392

[51] Liang Y Zheng X Zeng DD : A survey on big data-driven digital phenotyping of mental health. *Inform Fusion.* 2019;52:290–307. 10.1016/j.inffus.2019.04.001

[52] Wright AGC Woods WC : Personalized models of psychopathology. *Annu Rev Clin Psychol.* 2020;16(1):49–74. 10.1146/annurev-clinpsy-102419-125032 32070120

[53] Murray G Gottlieb J Hidalgo MP : Measuring circadian function in bipolar disorders: empirical and conceptual review of physiological, actigraphic, and self-report approaches. *Bipolar Disord.* 2020;22(7): 693–710. 10.1111/bdi.12963 32564457

[54] Scott J Colom F Young A : An evidence map of actigraphy studies exploring longitudinal associations between rest-activity rhythms and course and outcome of bipolar disorders. *Int J Bipolar Disord.* 2020;8(1): 37. 10.1186/s40345-020-00200-6 33258017 PMC7704984

[55] Van Someren EJ Hagebeuk EE Lijzenga C : Circadian rest-activity disturbances in Alzheimer's disease. *Biol Psychiatry.* 1996;40(4):259–270. 10.1016/0006-3223(95)00370-3 8871772

[56] Fekedulegn D Andrew ME Shi M : Actigraphy-based assessment of sleep parameters. *Ann Work Expo Health.* 2020;64(4):350–367. 10.1093/annweh/wxaa007 32053169 PMC7191872

[57] Anikwe CV Nweke HF Ikegwu AC : Mobile and wearable sensors for data-driven health monitoring system: state-of-the-art and future prospect. *Expert Syst Appl.* 2022;202: 117362. 10.1016/j.eswa.2022.117362

[58] Chen ZS Kulkarni PP Galatzer-Levy IR : Modern views of machine learning for precision psychiatry. *Patterns (N Y).* 2022;3(11): 100602. 10.1016/j.patter.2022.100602 36419447 PMC9676543

[59] Abd-Alrazaq A AlSaad R Shuweihdi F : Systematic review and meta-analysis of performance of wearable Artificial Intelligence in detecting and predicting depression. *NPJ Digit Med.* 2023;6(1): 84. 10.1038/s41746-023-00828-5 37147384 PMC10163239

[60] Tietbohl-Santos B Salgado TA Roza TH : Chapter 7 - Precision psychiatry in bipolar disorder.In: *Biomarkers in bipolar disorders.*R. Machado-Vieira and J.C. Soares, Editors. Academic Press,2022;115–124. 10.1016/B978-0-12-821398-8.00001-1

[61] Fried EI Robinaugh DJ : Systems all the way down: embracing complexity in mental health research. *BMC Med.* 2020;18(1): 205. 10.1186/s12916-020-01668-w 32660482 PMC7359484

[62] Hadaeghi F Golpayegani MRH Jafari S : Toward a complex system understanding of bipolar disorder: a chaotic model of abnormal circadian activity rhythms in euthymic bipolar disorder. *Aust N Z J Psychiatry.* 2016;50(8):783–92. 10.1177/0004867416642022 27164924

[63] Grierson AB Hickie IB Naismith SL : Circadian rhythmicity in emerging mood disorders: state or trait marker? *Int J Bipolar Disord.* 2016;4(1): 3. 10.1186/s40345-015-0043-z 26763505 PMC4712181

[64] Merikangas KR Swendsen J Hickie IB : Real-time mobile monitoring of the dynamic associations among motor activity, energy, mood, and sleep in adults with bipolar disorder. *JAMA Psychiatry.* 2019;76(2):190–198. 10.1001/jamapsychiatry.2018.3546 30540352 PMC6439734

[65] Shou H Cui L Hickie I : Dysregulation of objectively assessed 24–hour motor activity patterns as a potential marker for Bipolar I Disorder: results of a community-based family study. *Transl Psychiatry.* 2017;7(8):e1211. 10.1038/tp.2017.136 28892068 PMC5611716

[66] Kahawage P : The impact of chronotherapeutic interventions on mood: a systematic scoping review across multiple populations. *Clin Psychol Rev.* Under review.

[67] Esaki Y Obayashi K Saeki K : Circadian variability of objective sleep measures predicts the relapse of a mood episode in bipolar disorder: findings from the APPLE cohort. *Psychiatry Clin Neurosci.* 2023;77(8):442–448. 10.1111/pcn.13556 37092883

[68] Heath RA Murray G : Multifractal dynamics of activity data in bipolar disorder: towards automated early warning of manic relapse. *Fractal Geometry and Nonlinear Analysis in Medicine and Biology.* 2016;2(1):140–149. 10.15761/FGNAMB.1000124

[69] Indic P Salvatore P Maggini C : Scaling behavior of human locomotor activity amplitude: association with bipolar disorder. *PLoS One.* 2011;6(5): e20650. 10.1371/journal.pone.0020650 21655197 PMC3105113

[70] Ferrand L Hennion V Godin O : Which actigraphy dimensions predict longitudinal outcomes in bipolar disorders? *J Clin Med.* 2022;11(8):2204. 10.3390/jcm11082204 35456294 PMC9027161

[71] Porter RJ Moot W Inder ML : Validation of the Longitudinal Interval Follow-up Evaluation for the long-term measurement of mood symptoms in Bipolar Disorder. *Brain Sci.* 2022;12(12):1717. 10.3390/brainsci12121717 36552176 PMC9776034

[72] Earley W Durgam S Lu K : Clinically relevant response and remission outcomes in cariprazine-treated patients with bipolar I disorder. *J Affect Disord.* 2018;226:239–244. 10.1016/j.jad.2017.09.040 29017067

[73] Montgomery SA Asberg M : A new depression scale designed to be sensitive to change. *Br J Psychiatry.* 1979;134:382–389. 10.1192/bjp.134.4.382 444788

[74] Young RC Biggs JT Ziegler VE : A rating scale for mania: reliability, validity and sensitivity. *Br J Psychiatry.* 1978;133:429–35. 10.1192/bjp.133.5.429 728692

[75] Guy W : Clinical global impression scale. The ECDEU assessment manual for psychopharmacology-revised. DHEW, Publ No ADM 76.1976. Reference Source

[76] Keller MB Lavori PW Friedman B : The Longitudinal Interval Follow-up Evaluation: a comprehensive method for assessing outcome in prospective longitudinal studies. *Arch Gen Psychiatry.* 1987;44(6):540–548. 10.1001/archpsyc.1987.01800180050009 3579500

[77] Etain B Meyrel M Hennion V : Can actigraphy be used to define lithium response dimensions in bipolar disorders? *J Affect Disord.* 2021;283:402–409. 10.1016/j.jad.2021.01.060 33581466

[78] Malhi GS Bell E Bassett D : The 2020 royal Australian and New Zealand college of psychiatrists clinical practice guidelines for mood disorders. *Aust N Z J Psychiatry.* 2021;55(1):7–117. 10.1177/0004867420979353 33353391

[79] Ioannidis JPA : Pre-registration of mathematical models. *Math Biosci.* 2022;345: 108782. 10.1016/j.mbs.2022.108782 35090877

[80] Nosek BA Ebersole CR DeHaven AC : The preregistration revolution. *Proc Natl Acad Sci U S A.* 2018;115(11):2600–2606. 10.1073/pnas.1708274114 29531091 PMC5856500

[81] Dirnagl U : Preregistration of exploratory research: learning from the golden age of discovery. *PLoS Biol.* 2020;18(3): e3000690. 10.1371/journal.pbio.3000690 32214315 PMC7098547

[82] Beam AL Manrai AK Ghassemi M : Challenges to the reproducibility of machine learning models in health care. *JAMA.* 2020;323(4):305–306. 10.1001/jama.2019.20866 31904799 PMC7335677

[83] Bertone E Sahin O Stewart RA : Role of financial mechanisms for accelerating the rate of water and energy efficiency retrofits in Australian public buildings: hybrid Bayesian network and system dynamics modelling approach. *Appl Energy.* 2018;210:409–419. 10.1016/j.apenergy.2017.08.054

[84] Brase JM Brown DL : Modeling, simulation and analysis of complex networked systems: a program plan.Lawrence Livermore National Laboratory,2009.

[85] Freeborn L Andringa S Lunansky G : Network analysis for modeling complex systems in SLA research. *Studies in Second Language Acquisition.* 2023;45(2):526–557. 10.1017/S0272263122000407

[86] Lambiotte R Rosvall M Scholtes I : From networks to optimal higher-order models of complex systems. *Nat Phys.* 2019;15(4):313–320. 10.1038/s41567-019-0459-y 30956684 PMC6445364

[87] Keinan A Hilgetag CC Meilijson I : Causal localization of neural function: the Shapley value method. *Neurocomputing.* 2004;58–60:215–222. 10.1016/j.neucom.2004.01.046

[88] Fakhar K Hilgetag CC : Systematic perturbation of an Artificial Neural Network: a step towards quantifying causal contributions in the brain. *PLoS Comput Biol.* 2022;18(6): e1010250. 10.1371/journal.pcbi.1010250 35714139 PMC9246164

[89] Fakhar K Dixit S Hadaeghi F : Downstream network transformations dissociate neural activity from causal functional contributions. *Sci Rep.* 2024;14(1): 2103. 10.1038/s41598-024-52423-7 38267481 PMC10808222

[90] Fretter C Lesne A Hilgetag CC : Topological determinants of self-sustained activity in a simple model of excitable dynamics on graphs. *Sci Rep.* 2017;7(1): 42340. 10.1038/srep42340 28186182 PMC5301238

[91] Messé A Hütt MT König P : A closer look at the apparent correlation of structural and functional connectivity in excitable neural networks. *Sci Rep.* 2015;5(1): 7870. 10.1038/srep07870 25598302 PMC4297952

[92] Messé A Hütt MT Hilgetag CC : Toward a theory of coactivation patterns in excitable neural networks. *PLoS Comput Biol.* 2018;14(4): e1006084. 10.1371/journal.pcbi.1006084 29630592 PMC5908206

[93] First MB Williams J Karg RS : Structured Clinical Interview for DSM-5, Research Version (SCID-5-RV). Arlington, VA: American Psychiatric Association,2015.

[94] Taylor DJ Wilkerson AK Pruiksma KE : Reliability of the structured clinical interview for DSM-5 sleep disorders module. *J Clin Sleep Med.* 2018;14(3):459–464. 10.5664/jcsm.7000 29458705 PMC5837848

[95] Kumarasamy V Goodfellow N Ferron EM : Evaluating the problem of fraudulent participants in health care research: multimethod pilot study. *JMIR Form Res.* 2024;8: e51530. 10.2196/51530 38833292 PMC11185910

[96] Posner K Brown GK Stanley B : The Columbia-Suicide severity rating scale: initial validity and internal consistency findings from three multisite studies with adolescents and adults. *Am J Psychiatry.* 2011;168(12):1266–77. 10.1176/appi.ajp.2011.10111704 22193671 PMC3893686

[97] Tohen M Frank E Bowden CL : The International Society for Bipolar Disorders (ISBD) task force report on the nomenclature of course and outcome in bipolar disorders. *Bipolar Disord.* 2009;11(5):453–73. 10.1111/j.1399-5618.2009.00726.x 19624385

[98] Alloy LB Boland EM Ng TH : Low social rhythm regularity predicts first onset of bipolar spectrum disorders among at-risk individuals with reward hypersensitivity. *J Abnorm Psychol.* 2015;124(4):944–952. 10.1037/abn0000107 26595474 PMC4662076

[99] Fiedorowicz JG Merranko JA Iyengar S : Validation of the youth mood recurrences risk calculator in an adult sample with bipolar disorder. *J Affect Disord.* 2021;295:1482–1488. 10.1016/j.jad.2021.09.037 34563392

[100] Inder ML Crowe MT Luty SE : Randomized, controlled trial of interpersonal and social rhythm therapy for young people with bipolar disorder. *Bipolar Disord.* 2015;17(2):128–138. 10.1111/bdi.12273 25346391

[101] Simon GE Ludman EJ Bauer MS : Long-term effectiveness and cost of a systematic care program for bipolar disorder. *Arch Gen Psychiatry.* 2006;63(5):500–508. 10.1001/archpsyc.63.5.500 16651507

[102] Michalak EE Murray G, CREST.BD : Development of the QoL.BD: a disorder-specific scale to assess quality of life in bipolar disorder. *Bipolar Disord.* 2010;12(7):727–740. 10.1111/j.1399-5618.2010.00865.x 21040290

[103] Morton E Murray G Yatham LN : The Quality of Life in Bipolar Disorder (QoL.BD) questionnaire a decade on – a systematic review of the measurement of condition-specific aspects of quality of life in bipolar-disorder. *J Affect Disord.* 2021;278:33–45. 10.1016/j.jad.2020.09.017 32949871

[104] Carney CE Buysse DJ Ancoli-Israel S : The consensus sleep diary: standardizing prospective sleep self-monitoring. *Sleep.* 2012;35(2):287–302. 10.5665/sleep.1642 22294820 PMC3250369

[105] Legates TA Fernandez DC Hattar S : Light as a central modulator of circadian rhythms, sleep and affect. *Nat Rev Neurosci.* 2014;15(7):443–454. 10.1038/nrn3743 24917305 PMC4254760

[106] Moore-Ede MC Sulzman FM Fuller CA : The clocks that time us : physiology of the circadian timing system. Cambridge, MA: Harvard University Press,1982. Reference Source

[107] Figueiro MG Hamner R Bierman A : Comparisons of three practical field devices used to measure personal light exposures and activity levels. *Light Res Technol.* 2013;45(4):421–434. 10.1177/1477153512450453 24443644 PMC3892948

[108] Rea MS Bierman A Figueiro MG : A new approach to understanding the impact of circadian disruption on human health. *J Circadian Rhythms.* 2008;6(1):7. 10.1186/1740-3391-6-7 18510756 PMC2430544

[109] Cain SW McGlashan EM Vidafar P : Evening home lighting adversely impacts the circadian system and sleep. *Sci Rep.* 2020;10(1): 19110. 10.1038/s41598-020-75622-4 33154450 PMC7644684

[110] Wu Y : Elastic net for cox's proportional hazards model with a solution path algorithm. *Stat Sin.* 2012;22:27–294. 10.5705/ss.2010.107 23226932 PMC3515861

[111] Murphy KP : Dynamic Bayesian networks: representation, inference and learning.In: *Computer science.*University of California, Berkeley,2002. Reference Source

[112] Iorfino F Varidel M Marchant R : The temporal dependencies between social, emotional and physical health factors in young people receiving mental healthcare: a dynamic Bayesian network analysis. *Epidemiol Psychiatr Sci.* 2023;32:e56. 10.1017/S2045796023000616 37680185 PMC10539737

[113] Blumberg HP : Euthymia, depression, and mania: what do we know about the switch? *Biol Psychiatry.* 2012;71(7):570–1. 10.1016/j.biopsych.2012.02.003 22424111 PMC3874046

[114] Hadaeghi F Hashemi Golpayegani MR Moradi K : Does "crisis-induced intermittency" explain bipolar disorder dynamics? *Front Comput Neurosci.* 2013;7: 116. 10.3389/fncom.2013.00116 23986691 PMC3750208

[115] Bayani A Hadaeghi F Jafari S : Critical slowing down as an early warning of transitions in episodes of bipolar disorder: a simulation study based on a computational model of circadian activity rhythms. *Chronobiol Int.* 2017;34(2):235–245. 10.1080/07420528.2016.1272608 28060532

[116] Vandenbroucke JP von Elm E Altman DG : Strengthening the Reporting of Observational Studies in Epidemiology (STROBE): explanation and elaboration. *PLoS Med.* 2007;4(10):e297. 10.1371/journal.pmed.0040297 17941715 PMC2020496

[117] Lauder S Chester A Castle D : Development of an online intervention for bipolar disorder. www.moodswings.net.au. *Psychol Health Med.* 2013;18(2):155–165. 10.1080/13548506.2012.689840 22712771

[118] Lauder S Berk M Castle D : www.moodswings: the highs and lows of an online intervention for bipolar disorder — preliminary findings. *Aust N Z J Psychiatry.* 2008;42(Supplement 3):A83–4. Reference Source

[119] Murray G Thomas N Michalak EE : Mindfulness-based online intervention to improve quality of life in late-stage bipolar disorder: a randomized clinical trial. *J Consult Clin Psychol.* 2021;89(10):830–844. 10.1037/ccp0000684 34807658

[120] Fletcher K Lindblom K Seabrook E : Pilot testing in the wild: feasibility, acceptability, usage patterns, and efficacy of an integrated web and smartphone platform for Bipolar II Disorder. *JMIR Form Res.* 2022;6(5): e32740. 10.2196/32740 35639462 PMC9198820

[121] Fletcher K Foley F Thomas N : Web-based intervention to improve quality of life in late stage bipolar disorder (ORBIT): randomised controlled trial protocol. *BMC Psychiatry.* 2018;18(1): 221. 10.1186/s12888-018-1805-9 30001704 PMC6044003

[122] Michalak EE Lane K Hole R : Towards a better future for Canadians with bipolar disorder: principles and implementation of a community-based participatory research model. *Engaged Scholar Journal.* 2015;1(1):132–147. 10.15402/esj.v1i1.41

[123] Michalak EE Jones S Lobban F : Harnessing the potential of community-based participatory research approaches in bipolar disorder. *Int J Bipolar Disord.* 2016;4(4):1–9. 10.1186/s40345-016-0045-5 26856996 PMC4746206

[124] Michalak EE Suto MJ Barnes SJ : Effective self-management strategies for bipolar disorder: a community-engaged Delphi consensus consultation study. *J Affect Disord.* 2016;206:77–86. 10.1016/j.jad.2016.06.057 27466745

[125] Cohen JP Cao T Viviano JD : Problems in the deployment of machine-learned models in health care. *CMAJ.* 2021;193(35):E1391–E1394. 10.1503/cmaj.202066 34462316 PMC8443295

[126] Kraus B Sampathgiri K Mittal VA : Accurate machine learning prediction in psychiatry needs the right kind of information. *JAMA Psychiatry.* 2024;81(1):11–12. 10.1001/jamapsychiatry.2023.4302 38019526

[127] Solomon DA Leon AC Coryell WH : Longitudinal course of bipolar I disorder: duration of mood episodes. *Arch Gen Psychiatry.* 2010;67(4):339–347. 10.1001/archgenpsychiatry.2010.15 20368510 PMC3677763

[128] Stirling RE Nurse ES Payne D : User experience of a seizure risk forecasting app: a mixed methods investigation. *Epilepsy Behav.* 2024;157: 109876. 10.1016/j.yebeh.2024.109876 38851123 PMC13529357

[129] Dols A Kupka RW Mathijssen H : The time has come to question the infinite maintenance treatment for bipolar disorders. *Bipolar Disord.* 2024;26(5):415–417. 10.1111/bdi.13447 38736270

[130] Wilkinson MD Dumontier M Aalbersberg IJJ : The FAIR guiding principles for scientific data management and stewardship. *Sci Data.* 2016;3(1): 160018. 10.1038/sdata.2016.18 26978244 PMC4792175

