# Forecasting the course of bipolar disorder using rest-activity rhythms: Protocol for a multi-study modelling project

**DOI:** 10.12688/wellcomeopenres.24189.2

**Published:** 2026-07-13

**Authors:** Greg Murray, Sandipan Ray, Richard Porter, Fatemeh Hadaeghi, Denny Meyer, Maree Choi, Sara Lapsley, Matthew Tennant, Bridgette Thwaites, Ly Nguyen, Hailey Tremain, Saibal Saha, Jan Scott

**Affiliations:** 1 Centre for Mental Health and Brain Sciences, Swinburne University of Technology, Melbourne, Victoria, Australia; 2 Department of Biotechnology, Indian Institute of Technology Hyderabad, Hyderabad, India; 3 Mental Health Clinical Research Unit, University of Otago, Christchurch, Otago, New Zealand; 4 Institute of Computational Neuroscience, Universitatsklinikum Hamburg-Eppendorf, Hamburg, Hamburg, Germany; 5 Department of Psychology, Carleton University, Ottawa, Canada; 6 Psychological Medicine, Newcastle University, Newcastle, UK

**Keywords:** bipolar disorder, relapse, actigraphy, prospective, circadian, sleep, activity, molecular clock

## Abstract

**Background:**

Risk of new episodes is a constant concern for people living with bipolar disorder (BD). Research into sleep and circadian rhythm disruption (SCRD) suggests that relapse risk may be measurable in 24-hour rest-activity rhythms (RAR) via actigraphy. The primary aim of the Tipping Point project is to develop an algorithm forecasting relapse based on RAR. A secondary aim is to explore SCRD mechanisms linking RAR to mood dynamics in BD. The mechanistic investigation involves analysis of biological correlates of RAR, and exploration of system instability as a predictor of two types of state transition (relapse and recovery).

**Method:**

Three prospective studies will enrol adults living with BD I or II. Study 1 (Australia) recruits *N* = 100 individuals with interepisode BD who will undergo three contiguous 26-week monitoring epochs. Each epoch commences with 14 days of actigraphy, followed by retrospective interviews at 13 and 26 weeks. RAR predictors of time to first relapse will be explored using three computational frameworks - statistical risk, probabilistic network graphs and non-linear dynamic models. Study 2 (India) replicates one of the 26-week epochs of Study 1, recruiting 25 individuals with BD and 25 matched healthy controls. Study 2 investigates the replicability of models identified in Study 1 and tests the mechanistic association between amplitude of the RAR and amplitude of clock gene expression. Study 3 (New Zealand) explores system instability as a mechanism, by investigating whether complex system indicators of relapse risk (from Study 1) also forecast recovery from acute episodes. In Study 3, actigraphy data will be collected from people experiencing a severe episode of BD depression ( *N* = 30) or mania ( *N* = 15).

**Discussion:**

The Tipping Point project uses a multi-study design to develop a mechanistically-informed predictive algorithm for relapse risk in BD.

## Plain Language Summary

Even with the best available treatments, people living with bipolar disorder (BD) remain at risk of new episodes of depression and mania. One of the most challenging features of BD for patients and clinicians is that we cannot reliably predict when an individual might experience a further episode. This paper describes the protocol for an international project that aims to help with this problem. The Tipping Point project builds on research into, (i) sleep and body clock disturbances in BD, and (ii) ongoing monitoring in rest- activity and sleep-wake patterns in BD using an actigraph (a wrist-worn movement sensor similar to, but a bit more sophisticated than ‘consumer wearables’ such as Fitbits or sports watches). A collaborative team of researchers, clinicians, and lived experience experts have designed three studies which, together, will support development of an algorithm (based on rest-activity patterns) that forecasts relapse risk and response to treatment in BD. Of course, we appreciate our project will not answer all the questions people have about predicting BD outcomes, but we will share our data and our algorithm with other groups around the world to see if our findings can be replicated in different populations and geographical locations. A particular advantage of the project is that it offers a low-cost, non-invasive method that allows both clinicians and individuals with BD to spot early warning signs and symptoms of relapse and enables people to better manage their BD.

## 1. Introduction

Bipolar disorder (BD) is a serious episodic mood disorder associated with significant morbidity and mortality. ^1^ The psychological, familial, social, and clinical burden of BD is reflected in its economic cost. ^2^^,^^3^ In Australia, the excess health care costs of BD are about AU$7.3 billion per annum. ^4^ It is estimated that three-quarters of this cost could be saved with improved management, particularly avoidance of hospitalisation via early warning of impending relapse. ^4^ The present project takes up the challenge of improving forecasting of the short-term course (weeks and months) of BD, with the clinical aim of identifying an algorithm for BD relapse risk based on rest-activity rhythms (RAR).

The course of BD remains poorly understood, but we do know it is highly heterogeneous within and between individuals, ^5^ and clinical characteristics (prior course, residual symptoms, etc.) are only modest predictors of relapse and recurrence. ^6^ Emerging computational approaches have great potential to improve understanding of the course of mood disorders, with models ranging from purely data-driven [e.g., 7, 8] to theoretically-informed [e.g., 9, 10]. Here, the latter approach is prioritised, leveraging off rapidly growing understanding of sleep and circadian rhythm disruption (SCRD) in psychopathology. ^11^^,^^12^

The SCRD framework has unique strengths as an explanatory approach to BD. First, recent reviews highlight evidence for SCRD involvement in BD’s development and course, and specifically that SCRD variables may act as predictive biomarkers. ^13^^–^^15^ Secondly, an associated passive data collection methodology – 24-hour rest-activity rhythms (RAR) measured by actigraphy – supports investigation of chronobiology in the real world. ^16^ Furthermore, actigraphy is non-invasive and inexpensive, and could potentially translate to an automated early warning tool. ^17^^,^^18^

The primary aim of this project is to develop an SCRD-informed, RAR based algorithm forecasting relapse risk in BD. Confidence in the reliability and generalisability of a predictive algorithm would be strengthened with improved mechanistic understanding of SCRD in BD as measured by RAR in this forecasting context. Consequently, the project’s secondary aim is to contribute to mechanistic understanding by, (i) investigating molecular biological correlates of RAR parameters, and (ii) exploring system instability as a predictor of both relapse into, and recovery from acute episodes (with both phenomena understood as transitions in state space [e.g., 19]).

### 1.1 Introduction to the Circadian System and Sleep

Evolution on earth has favoured organisms that anticipate, rather than react to, the planet’s daily shifts between light and dark. The embodiment of this ‘predictive homeostasis’ is the circadian system, which coordinates internal physiology and environmental activity in most species. ^20^ The adaptive endpoint of biological clockwork is circadian (approximately 24-hour) rhythmicity in biological (e.g., core body temperature), behavioural (the activity-rest cycle), and mental (attention, affect) processes.

The molecular genetics of circadian rhythmicity is well characterised in mammals. ^21^ Within the master oscillator of the suprachiasmatic nucleus (SCN), self-sustained rhythmicity is generated by a primary intra-cell autoregulatory transcription-translation feedback loop (TTFL) involving the activators CLOCK and BMAL1, and their target genes *PER1, PER2, CRY1* and *CRY2*, whose products form a negative-feedback repressor complex [additional stabilisation and auxiliary loops interlock with this core process, 22]. Similar cellular clocks operate throughout the body, receiving inputs from the SCN to coordinate internal timing across tissues: Diurnal variation in clock gene expression in red blood cells, for example, has been used as an indicator of circadian amplitude and phase. ^23^^,^^24^ The secondary mechanistic aim of the present project includes investigation of the association between blood-based circadian markers (gene expression and associated metabolites) and RAR parameters.

From a circadian viewpoint, the sleep-wake cycle is the most visible circadian rhythm in humans. ^25^ From the viewpoint of sleep *per se*, the circadian system is one of two primary drivers of sleep timing [the other being a homeostatic sleep drive, 26]. Parsing circadian from sleep processes in humans is a methodological challenge, traditionally addressed by sleep-disrupting laboratory protocols that are unsuitable for people with BD. ^27^ As discussed below, contemporary translational research is increasingly using passive data collection and computational methods to study these processes in everyday life.

### 1.2 Sleep and Circadian Rhythm Disruption may be a Predictive Biomarker in BD

Sleep and circadian rhythm disturbances are common symptoms of BD episodes but have also been shown to precede full-threshold episodes [e.g., 28– 33]. For example, candidate gene association studies provide some support for associations between BD and common variants in *CLOCK* [the ClockΔ19-mutant mouse is the best-characterised animal model of BD, 34], and variants of *ARNTL* [see, for a review, 15]. A recent international expert review concluded that SCRD plays an important role in mental health, but causal pathways remain poorly understood. ^35^ In mood disorders, several SCRD pathways have been proposed to be mechanistically important. ^36^^–^^38^ For example, decreased robustness of circadian rhythmicity – measurable in decreased amplitude of downstream RAR – may be an endophenotype of BD [e.g., 13, 15, 39– 42].

### 1.3 Actigraphy

Actigraphy was originally used to measure sleep patterns, where it has been shown to be reliable and valid, ^43^ including amongst individuals with BD. ^44^^,^^45^ The last decade has seen rapidly increasing interest in actigraphy due to: (i) recognition of activity disturbance as core to the BD phenotype ^46^^,^^47^; (ii) its promotion by the US National Institute of Mental Health as a method for investigating arousal and regulatory systems ^48^; (iii) growing research into digital phenotyping and passive sensing via wearables, ^49^^–^^52^ and (iv) emerging personalised models of psychopathology. ^8^^,^^53^

Polysomnography (PSG) is the gold standard measure of sleep patterns and circadian markers of sleep-wake cycles. However, studies using actigraphy are overtaking PSG as the former offers an ecologically valid measurement of individual chronobiological functioning in everyday life. ^54^ Actigraphy research in BD has moved beyond the analysis of sleep parameters generated from the manufacturers’ software programmes (e.g., mean and standard deviation estimates of total sleep time, sleep onset latency, sleep efficiency, wake after sleep onset) to include estimation of RAR parameters that may be more critical for mental disorders such as stability, variability or predictability of 24-hour sleep-wake cycles and the regularity of timing of activity. ^55^ Other work in BD has investigated actigraphy as a measure of chronotype [often delayed in BD compared to healthy controls, 54] and a range of sleep timing variables. ^56^

Prominent in this RAR work are three non-parametric variables originally proposed by Van Someren and colleagues: Relative Amplitude, Intradaily Variability, and Interdaily Stability. ^57^ Amongst these ‘Van Someren variables’, Relative Amplitude is of particular interest in this project. Understood as a measure of circadian robustness, Relative Amplitude (the difference between most and least active periods compared with total activity per 24 hours) has been associated with a range of BD phenotypes and outcomes. ^16^

Actigraphy generates a high-resolution data stream (14 days’ recording generates 1.2 million data points), supporting a range of analyses, including but not limited to the macro-level sleep and circadian proxy variables introduced above [see, 54]. Actigraphic measurement figures prominently in computational models of mental health, ^58^^,^^59^ and mood disorders particularly [e.g., 60, 61]. The present project uses three complementary frameworks to advance prediction of BD course from RAR – statistical risk (Cox Survival Analysis), probabilistic network graphs (Dynamic Bayesian Network Analysis) and non-linear dynamic analysis (Hadaeghi’s Complex System Model of BD, 19). The RAR measures to be modelled as predictors in these analyses include the three non-parametric Van Someren variables (above). Relative Amplitude is elevated as a correlate of circadian biology in the mechanistic investigation of Study 2 (see below).

*Actigraphic Measurement of the 24-h Rest-Activity Rhythm can Predict Course of BD*

A substantial body of research has investigated *cross-sectional* associations between actigraphy-measured RAR variables and BD phenotypes. A recent systematic review identified 70 studies investigating RAR via actigraphy in BD samples. ^16^ Attenuated amplitude of RAR has been shown to associate with several BD phenotypes. ^62^^–^^64^ Consistent with the circadian robustness hypothesis (above), the largest study to date ^31^ found that a one quintile (20%) decrease in Relative Amplitude was associated with a small but significant increased risk of lifetime major depressive disorder (OR 1.06, 95% CI 1.04–1.08) and lifetime BD (OR 1.11, 1.03–1.20) [ 1].

Several groups have argued for the potential of RAR signals to generate a clinically-useful predictive algorithm of short-term course in mood disorders and BD specifically. ^10^^,^^16^^,^^18^^,^^43^^,^^66^^–^^68^ Prospective empirical investigations are limited to date, but an illustrative example is the study of Ferrand and colleagues, in which RAR was sampled with 14 days actigraphy data before 69 euthymic individuals with BD were prospectively tracked for a median duration of 3.5 years. ^69^ Approximately 65% of participants experienced a relapse or recurrence during follow-up. After adjusting for key clinical variables (age, sex, BD subtype, number of mood stabilisers, etc.), time to episode onset was predicted by an actigraphy dimension comprising amplitude and variability/stability of circadian rhythms ( *p* = 0.009). The area under the curve (AUC) was improved from 0.64 for clinical predictors alone to 0.82 with the addition of actigraphy-derived Intradaily Variability ( *p* = 0.04).

A recent evidence-map review of prospective studies by Scott and colleagues investigated actigraphy parameters as predictors of course, outcome, and treatment response in BD. ^55^ Scott et al. conclude actigraphy markers have potential to predict future mental state, with the strongest individual predictors being attenuated amplitude and delayed sleep onset. The authors note significant limitations in existing literature, including small sample sizes, varied endpoints, and an unsystematic approach to covariates and statistical modelling.

### 1.4 The Tipping Point project

The Tipping Point project is fundamentally exploratory. Its primary aim is to *develop and cross-validate an RAR algorithm of relapse risk for BD.* The algorithm could form the basis of a future actigraphy-based early warning device for people living with BD. The project applies three analytic frameworks (see 2.2 and 2.6 below) to develop predictions of BD course from RAR – statistical risk (Cox Survival Analysis), probabilistic network graphs (Dynamic Bayesian Network Analysis) and non-linear dynamic analysis (Hadaeghi’s Complex System Model of BD).

The project’s secondary aim is to contribute to improved mechanistic understanding of SCRD in BD as measured by RAR in this forecasting context. Consistent with the brief literature review above, two types of mechanism warrant investigation. First, to ground the actigraphy signal in circadian biology, RAR parameters will be correlated with circadian gene expression patterns amongst people with BD and a matched control sample. Second, by conceptualising relapse in non-linear dynamic terms as a state transition, we explore whether Hadaeghi’s Complex System Model ^19^ applied to prediction of *relapse* generalises to prediction of an alternative clinically significant state transition, namely, *response to treatment* for an acute episode.

The project involves four work packages. Three of these are prospective empirical studies amongst people living with BD. Study 1 (WP1, Australia) aims to predict early relapse by modelling RAR data. Study 2 (WP2, India) tests predictive models from RAR developed in Study 1 and tests the mechanistic relationship between a key RAR variable (Relative Amplitude, representing circadian robustness) and diurnal amplitude of gene expression and metabolites in blood. Study 3 (WP3, New Zealand) advances mechanistic understanding of the link between RARs and mood in BD by investigating a different state transition, namely, recovery from an acute episode, operationalised in response to evidence-based treatment. Thus, Studies 1 and 2 contribute to the project’s primary aim of algorithm development, and Studies 2 and 3 contribute (in different ways) to the secondary aim of improved mechanistic understanding of RAR variables in predicting BD course. Finally, WP4 is computational, conducting model comparison, cross-validation and synthesis using ensemble machine learning. A key output of WP4 is an open dataset and computational pipelines for sharing with future researchers.

## 2. Protocol

To ensure clarity and appropriate detail for a project with this hierarchical structure, a multi-paper registration strategy has been adopted. Study-specific methodological details essential for direct replication of each component (e.g., detailed assessment schedules, site-specific recruitment procedures, and embedded sub-studies) will be provided in three forthcoming, cross-referenced study-level protocols [ 2]. This manuscript presents the overarching project protocol, detailing the scientific rationale, the high-level design of the three empirical studies, and the cross-study analytic plan (WP4). Box 1 provides an overview and comparison of the three studies’ methods.

Box 1. **Comparison of methods across three empirical work packages.**

**Table:** 

Design Feature	Study 1 (Australia)	Study 2 (India)	Study 3 (New Zealand)
Primary Aim	Develop and train a predictive model for relapse risk from RAR data.	Replicate the relapse model in a low-middle income context and test biological correlates of RAR.	Test generalisability of the relapse model to predict treatment response (recovery).
Population	Adults with interepisode BD I or II.	Adults with interepisode BD I or II, plus matched healthy controls.	Adults experiencing an acute manic or depressive episode of BD I or II.
Setting	Outpatients/Community recruitment.	Outpatients/recruitment via hospital-based clinicians.	Predominantly inpatients, some outpatient recruitment via clinicians.
Primary Predictor	Actigraphy (RAR variables) over 14 days (at baseline of three epochs).	Actigraphy (RAR variables) over 14 days (at baseline of one epoch).	Actigraphy (RAR variables) over 14 days (at baseline).
Primary Outcome	Time to Relapse over three 26-week epochs (measured via retrospective LIFE interview on two occasions per epoch).	Time to Relapse over a 26-week epoch (measured via retrospective LIFE interview on two occasions over 26 weeks).	Treatment Response (≥50% symptom reduction) at 8 weeks.
Biological Sampling	No	Yes (blood samples) for circadian gene expression and metabolomics.	No
Clinical Predictors in models	Yes (five key clinical variables included as covariates, e.g., BD subtype, residual symptoms).	Yes (five clinical variables included to enable direct replication of Study 1).	No (to minimise participant burden in an acutely ill population).
Contribution to project	Core model development and initial validation.	Cross-cultural replication and mechanistic analysis via biological correlates.	Mechanistic analysis via testing of model generalisation from one form of state transition (relapse) to another (recovery)

### 2.1 Inclusion and exclusion criteria

The approach to sampling and inclusion/exclusion balances four principles: maximising generalisability of findings, maximising inclusivity for people living with BD, participant empowerment and duty of care.

*All studies*

In all studies, participants must have a diagnosis of BD I or II confirmed by structured clinical interview. Reason to believe that participation would interfere with treatment and/or recovery is an exclusion criterion. Participants must have sufficient language fluency (English in Studies 1 and 3; English, Hindi, Telegu, or Bengali in Study 2) to be able to provide informed consent and to complete self-report and interview-based assessments. Age range for inclusion is 18–65 years. This range was selected to limit variance associated with the distinct circadian and sleep changes of adolescence and later life and is conventional for operationalising the ‘adult’ life phase in mental health research. Similarly, age 65 is a common cut-off in BD [e.g., 70] and sleep/circadian research [e.g., 40].

Presence of a physical problem that might confound RAR interpretation is an exclusion. The decision about whether to exclude is made case-by-case, considering the sampling principles of generalisability and inclusivity (above). Screening for this exclusion differs by study. In Studies 1 and 2, participants are asked at screening about any physical conditions that impact movement, which are then followed up at interview. In Study 3, nurses involved in recruitment are trained to observe for movement (particularly Parkinsonian tremor in the inpatient setting) as a potential exclusion.

Presence of common BD comorbidities is not an exclusion criterion. Anxiety disorders, substance use disorders and sleep disorders will be assessed by structured clinical interview at baseline and explored for potential inclusion in predictive models. This approach allows us to build models that generalise to complex clinical presentations while still accounting for their impact on models.

*Study 1 and 2*

In Study 1 and 2, being in an episode *at baseline* is an exclusion criterion. Following common cut-offs [e.g., 71, 72] presence of an episode is defined as a score of > 12 on the Montgomery-Asberg Depression Rating Scale [MADRS, 73], or > 8 on the Young Mania Rating Scale [YMRS, 74] [ 3]. Meeting episode criteria at one of the follow-up interviews is, however, not an exclusion criterion. Considering patient empowerment, risk management and the scientific aims of tracking BD course over an extended period, it was decided on balance to make case-by-case decisions on whether ongoing participation is affected by identification of an episode at a follow-up interview (see below) [ 4].

On duty of care grounds, clinical participants in the outpatient Studies 1 and 2 must be currently receiving treatment for BD from a medical practitioner and provide contact details and consent for the doctor to be contacted if required. An additional exclusion criterion in Study 1 and 2 is the presence of active suicidality, measured at baseline and at each follow-up interview. Because of blood draws, a further exclusion criterion in Study 2 only is severe anaemia (Hb level < 7 g/d).

*Study 3*

Study 3 is distinct from Studies 1 and 2 in population, setting and primary outcome ( Box 1). Being in a severe episode of mania or bipolar depression is an inclusion criterion, as defined by a Clinical Global Impression Scale [CGI, 76] of 5 and above, and the majority of participants will be recruited as inpatients. Current intoxication or withdrawal from illicit substances are exclusions unique to Study 3: it is not uncommon for individuals to be admitted to hospital in a state of intoxication or acute withdrawal (e.g., from alcohol or methamphetamines). These states would profoundly impact the capacity to consent and confound the actigraphy data collected in the initial days.

*Written and Informed Consent*

In all studies, written informed consent will be obtained from each participant after they receive detailed explanations about the study procedure in the language best understood by them. Participants can withdraw from the study at any time but will not be able to withdraw data already provided. Participants will be invited to provide ‘extended’ consent so that data can be shared with appropriate research teams upon completion of this project. All participants will be assigned a study ID to be used in place of the participant’s name on all assessment materials. The list linking IDs and names will be held under the same security conditions as study data (see below).

### 2.2 Work packages

Three prospective monitoring studies are planned as three work packages. A fourth integrative work package will synthesise mechanistic and modelling findings from Studies 1–3 and develop the theoretically-informed relapse risk algorithm for testing in future independent samples.

*Study 1: Predicting relapse in interepisode BD from rest-activity rhythms*

**Figure 1.**
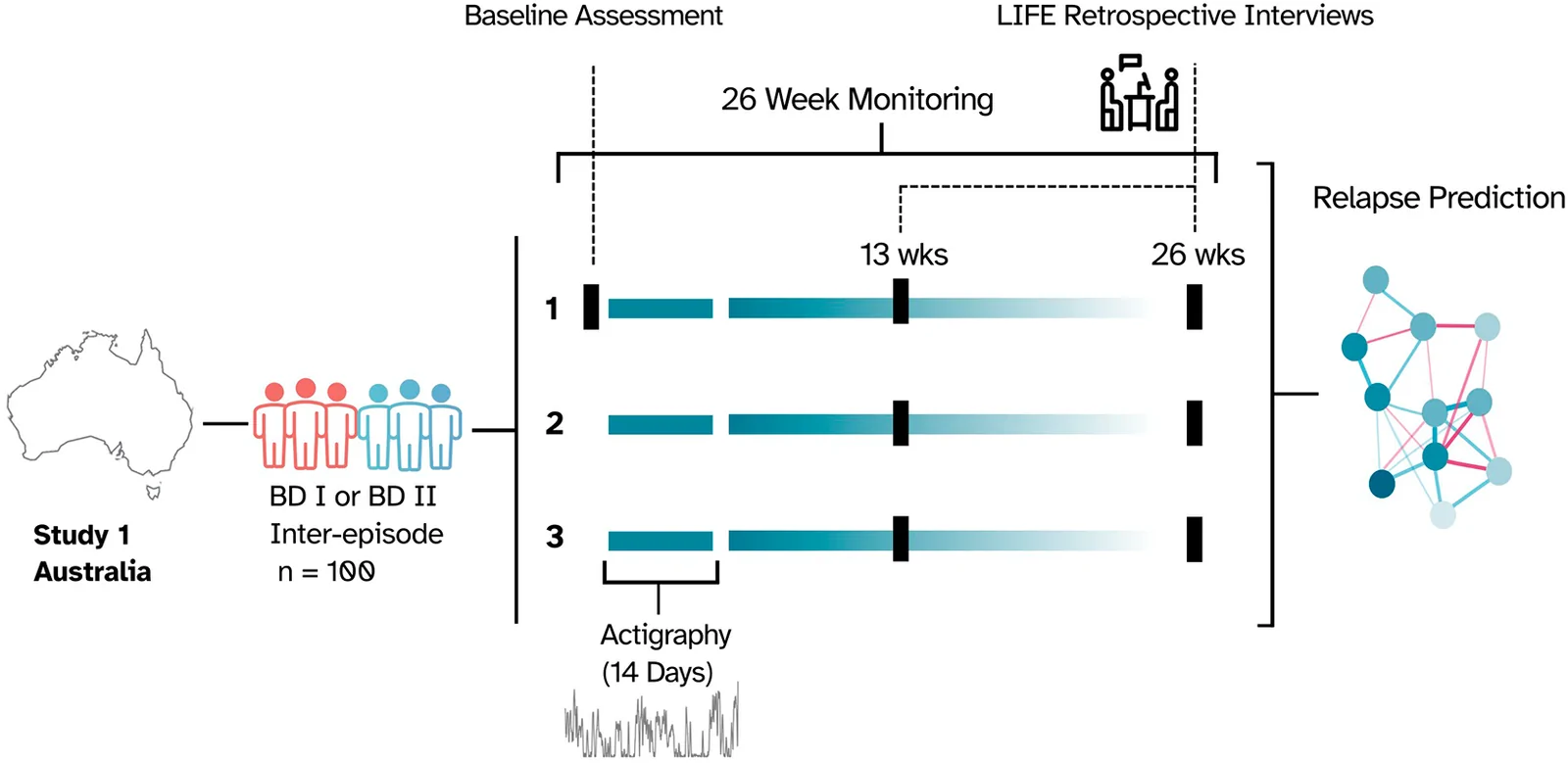
Study 1 design overview.

Study 1 (Australia) provides the primary 18-month dataset on which to conduct Cox Survival Analysis, develop Dynamic Bayesian Network models predicting evolution of symptoms and full-threshold relapse, and to train/test Hadaeghi’s Complex System Model. A total of *N* = 100 individuals with BD will wear actigraphs for 14 days at the start of each of three consecutive 26-week monitoring epochs (for a total window of 3 × 26 = 78 weeks forecasting, see Figure 1). In Study 1 (and Study 2), time to relapse will be measured on the retrospective LIFE interview ^77^ conducted at 13 and 26 weeks of each epoch. We propose that 26 weeks is a clinically meaningful timeframe for the present interest in course across weeks and months, as it generates translatable information for use in clinical settings (e.g., at initial assessment, or at the transition from inpatient to outpatient management).

The three analytic approaches generate distinct, complementary research questions for the project’s primary aim of algorithm development:

• Research Question 1: Do Relative Amplitude, Intradaily Variability, and/or Interdaily Stability emerge as independent predictors of time to relapse using Cox Survival Analysis?
• Research Question 2: Can a Dynamic Bayesian Network Analysis of RAR parameters predict change in BD psychopathology and full-threshold relapses in a sample of individuals with BD I and II?
• Research Question 3: Can Hadaeghi’s Complex System Model, trained on statistical, time-frequency, and nonlinear dynamic features from RAR data collected from a cohort of *n* = 75 participants predict relapse in an unseen subset of *n* = 25 participants?
• Research Question 4: Do the three approaches to analysis (Cox Survival Analysis, Dynamic Bayesian Network Analysis, and Hadaeghi’s Complex System Model) generate convergent findings about relapse risk prediction from RAR?

*Study 2: Exploring replicability of models in a lower-middle income sample; testing molecular rhythmicity correlates of RAR variables.*

**Figure 2.**
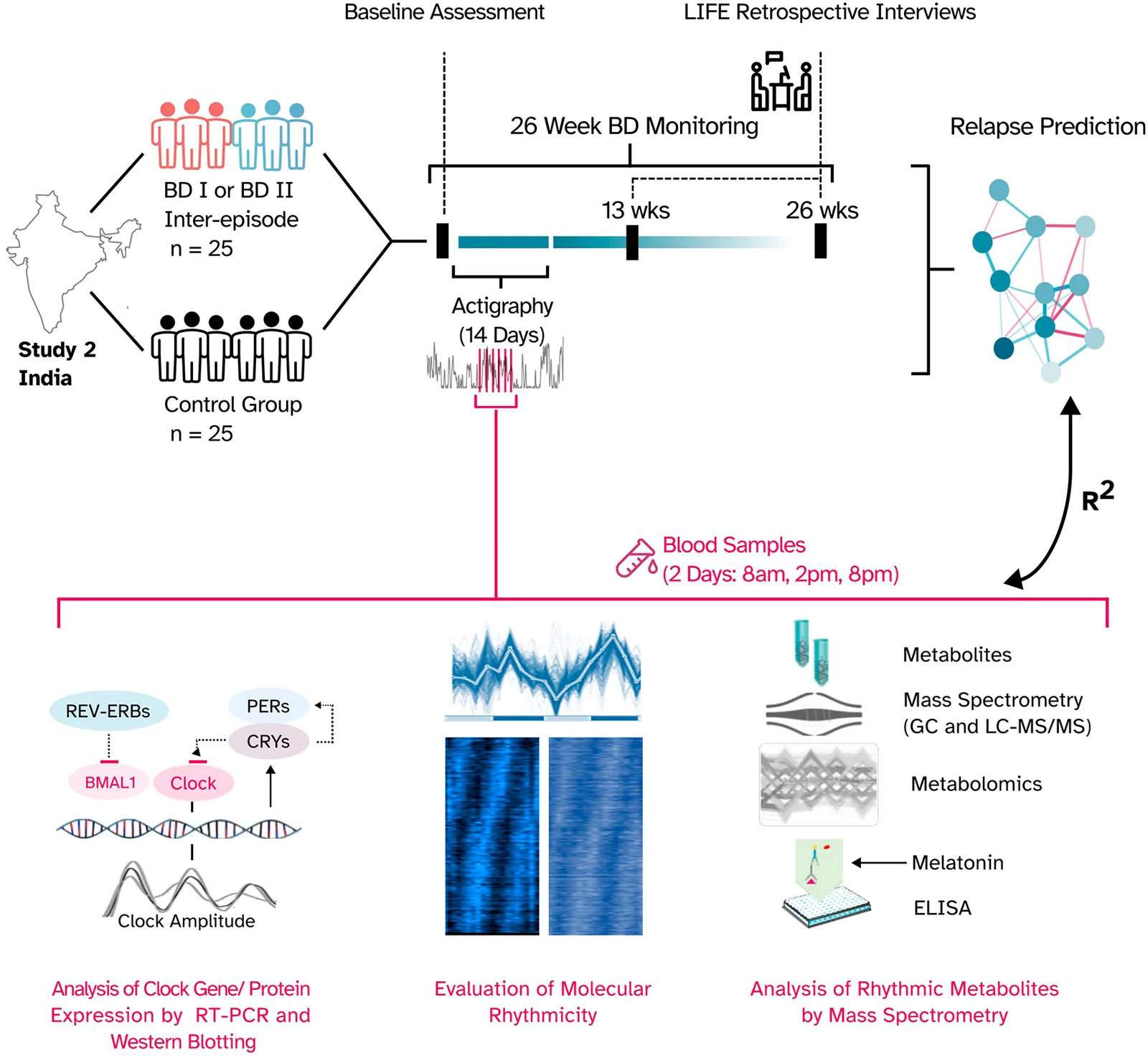
Study 2 design overview.

Study 2 (India) repeats Study 1’s prospective design amongst interepisode individuals with BD, but with only one 26-week epoch ( Figure 2). Study 2 aims, first, to explore the replicability of Study 1’s models of relapse prediction in an out-of-distribution cohort from a lower-middle income country (LMIC). Secondly, Study 2 will, (i) test the mechanistic prediction that Relative Amplitude will correlate with diurnal amplitudes of gene expression and metabolites in blood, and (ii) test the hypothesis that both Relative Amplitude and diurnal amplitudes of gene expression and metabolites in blood are attenuated in individuals with BD ( *N* = 25) relative to matched healthy controls ( *N* = 25).

Three research questions and two hypotheses are therefore set for Study 2:

• Research Question 1: Does the Cox Survival Analysis model derived in Study 1 replicate amongst *N* = 25 BD participants in an LMIC cohort?
• Research Question 2: Does Dynamic Bayesian Network Analysis enable examination of relapse prediction amongst *N* = 25 BD participants in an LMIC cohort?
• Research Question 3: Does Hadaeghi’s Complex System Model of relapse prediction derived in Study 1 replicate amongst *N* = 25 BD participants in an LMIC cohort?
• Hypothesis 1: Amongst the complete sample ( *N* = 50), Relative Amplitude will correlate with the diurnal amplitude of both circadian gene expression and blood metabolites.
• Hypothesis 2: Diurnal amplitude of circadian gene expression and blood metabolites, and Relative Amplitude will be attenuated in people with interepisode BD ( *N* = 25) compared to age, gender, and employment-matched healthy controls ( *N* = 25).

*Study 3: Exploring generalisability of the Complex System model of state transition from relapse prediction to treatment response.*

Study 3 contributes to the project’s secondary mechanistic aim by investigating if complex system features found in Study 1 to predict relapse (state transition into an episode) generalise to prediction of treatment response from an acute episode (state transition to recovery). If generalisation is demonstrated, it could be hypothesised that Hadaeghi’s Complex System Model captures generic system instability (as opposed to a relapse-specific process).

**Figure 3.**
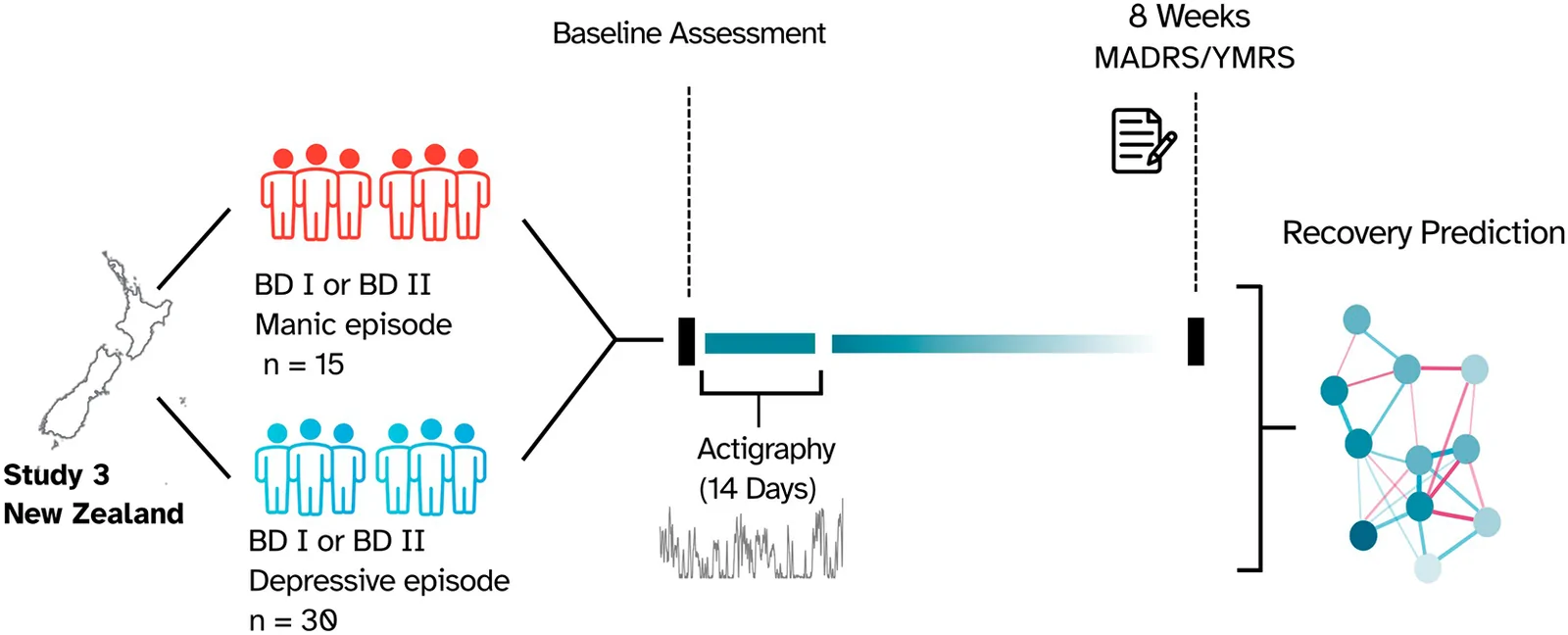
Study 3 design overview.

The primary aim of Study 3 is therefore to investigate whether the relapse risk algorithm informed by Hadaeghi’s Complex System Model (trained and tested on Study 1 data) can be extended to response to treatment. ^19^ The state transition of Study 3 is measured in binary response to treatment. Response to treatment at 8 weeks is operationalised as 50% reduction in MADRS (for those in depressive episode at baseline) or 50% reduction in YMRS (for those in manic episode at baseline). Participants will wear actigraphs for 21 days – for consistency with Study 1 and 2, the first 14 days only will be used in modelling of binary response to treatment. Figure 3 below summarises the study design for this primary aim.

Study 3 has two additional aims related to improved characterisation of associations between RAR signals and short-term mood dynamics. The secondary aim of Study 3 is to examine the effect of baseline episode polarity—that is, whether the acute episode is depressive or manic—on the performance and structure of the predictive model. The analysis of Study 3’s secondary aim will investigate whether polarity moderates model accuracy against the binary outcome of 50% reduction in symptoms at 8 weeks, using 14 days’ actigraphy data. To increase the granularity of the investigation of this state transition, a tertiary aim of Study 3 is to explore temporal patterns in weekly RAR in relation to weekly clinician ratings of mood (MADRS, YMRS) and clinical state (CGI). The process-focus of this exploratory analysis takes advantage of the full 21 days of actigraphy.

Study 3 therefore investigates the prediction of acute treatment response from RAR variables amongst people who are experiencing a severe episode of BD [an important clinical question in its own right, 78]. Participants will be individuals with BD receiving guideline-based care and treatment ^75^ for acute manic ( *N* = 15) or bipolar depressive ( *N* = 30) episodes (on an inpatient, outpatient, or hybrid basis). Participants will be patients of Te Whatu Ora-Waitaha Canterbury Mental Health Services.

Three research questions address the development and testing of a state transition model, and its potential moderation by baseline episode polarity.

• Research Question 1: How many additional samples ( *x*) from the *N* = 45 Study 3 sample are required to retrain the Study 1 complex system model predicting relapse ( *N* = 75) to achieve acceptable prediction accuracy for *a state transition model* predicting both relapse amongst interepisode patients and response to treatment amongst acutely ill patients?
• Research Question 2: Can a complex system model of state transition trained on *N* = 75 Study 1 samples and *x* (from Research Question 1) Study 3 samples predict state transition in an unseen data set of *N* = 25 Study 1 samples and N = (45 – *x*) samples from Study 3?
• Research Question 3: Is episode polarity at baseline in the Study 3 cohort ( *n* = 30 depression versus *n* = 15 manic) a moderator for the models developed in Research Question 1 and Research Question 2?

*Work Package 4: Integrative Data Synthesis*

Work Package 4 will develop an integrated relapse-risk algorithm that synthesises findings from the three preceding studies. The aim is to combine insights from statistical survival models, probabilistic network models, and non-linear dynamical models into a unified predictive framework. We will develop the integrated algorithm as a meta-model, using ensemble learning techniques to combine either the most informative features or the predicted probabilities from each of the component models. This approach will allow the algorithm to take advantage of the strengths of different analytical methods—statistical interpretability from survival models, multivariate pattern recognition from network models (and other forms of machine learning), and dynamic stability indicators from non-linear dynamic models. Model weighting, calibration, and validation will be performed using cross-validation and regularised optimisation to maximise generalisability and avoid overfitting. The resulting meta-model will provide individualised, probabilistic estimates of relapse risk, which can be iteratively refined as additional longitudinal data become available.

In addition to developing this ensemble meta-model, WP4 may include exploratory theoretical work aimed at integrating network-based and complex system approaches in the context of relapse prediction [see, e.g., 79– 82]. Two lines of innovation are of particular interest. First, multi-perturbation Shapley-value analysis (MSA) ^83^ has been extended in our group to study temporal causal connectivity in brain and artificial neural networks. ^84^^,^^85^ MSA can be applied here to rank the relative contribution of variables (or network nodes) to relapse prediction for any given predictive or dynamical model. Second, the team has experience with excitable network modelling, which examines how small perturbations can propagate through a dynamic graph to reveal relationships between structural and functional connectivity. ^86^^–^^88^ These methods will be explored if data structure and model performance indicate potential for further mechanistic insight.

A detailed statistical analysis plan specifying the ensemble architecture, validation approach, and any additional exploratory analyses will be finalised and published prior to conducting analyses.

### 2.3 Baseline assessment measures

*All studies*

In all three studies, diagnostic inclusion and exclusion criteria will be confirmed on the Structured Clinical Interview for DSM-5, Research Version [SCID-5-RV, 89], as will the presence of common anxiety and substance use comorbid diagnoses. The Structured Clinical Interview for Sleep Disorders-Revised [SCISD-R, 90] will be used to assess the presence of common sleep disorders at baseline.

*Study 1 and 2*

People interested in participating will be invited to visit the study website (or if internet is not readily available, provided with written documentation where they can review study details), and then complete a consent form and answer an initial screening survey on demographic and inclusion/exclusion criteria (age, sex/gender, currently receiving medical treatment for a diagnosis of BD I or II, presence of lifestyle or physical condition impacting RAR). Those who pass the online screening questions will be invited to an appointment for a screening interview, conducted online in Study 1 (Australia) and in person in Study 2 (India). Interviewers will confirm that the potential participant is engaged in medical care for their BD and confirm consent. To minimise fraudulent engagement with these paid studies, ^91^ participants’ identity will be confirmed in Study 1 by presentation of a photo ID during the teleconference. In Study 1 and 2, the exclusion criterion of active suicidality is operationalised as a score of 3, 4, or 5 on the Columbia Suicide Severity Rating Scale [CSSRS, 92].

*Study 3*

In Study 3, participants must be in an episode of mania or bipolar depression at baseline as assessed by a senior psychiatrist of the health service and have a CGI score of 5 and above (markedly ill).

### 2.4 Primary outcomes

*Study 1 and 2: Time to relapse*

In Study 1 and 2, the time to relapse of any BD episode (manic, hypomanic, depressive) will be assessed using the LIFE [ 5]. The LIFE was developed to retrospectively assess the longitudinal course of psychiatric disorders over extended periods. Weekly data are elicited retrospectively, in an interview format. The method allows for the overall quantification of mood burden, through the interviewer determining ordinal weekly scores for severity of depression and mania. Patients are rated on a 1–6 scale, where 1—no symptoms, 2—residual symptoms, 3—partial remission, 4—does not meet DSM criteria but has major symptoms or impairment, 5—meets definite DSM criteria for an “episode”, and 6—fulfills definite criteria for an “episode” with the presence of either psychotic symptoms or extreme impairment in functioning. By determining whether syndromal symptoms last 2 weeks, relapse into a depressive or manic episode can be determined. The LIFE method has been used in several longer-term studies in BD to determine time to episode occurrence and overall mood morbidity [e.g., 79– 82]. The retrospective LIFE avoids the problem of participant burden with prospective (e.g., weekly or monthly) measurement in long-term outcome research, and in BD populations has the additional strength of minimising the confound of episode-related impaired insight. Porter and colleagues have recently published an analysis of two studies validating the LIFE’s retrospective weekly estimates against concurrently rated clinician ratings on the MADRS and YMRS. ^72^

In Study 1 and 2, LIFE interviews will occur twice in each monitoring epoch (13 weeks, and 26 weeks, with 13 weeks’ retrospection window at each interview). The LIFE is commonly administered at 6-month intervals, and there is reason to believe that the 13-week intervals here will improve the reliability and resolution of our estimate of time to relapse. ^71^^,^^77^ Because of remote recruitment, the LIFE will be conducted by telehealth in Study 1. In Study 2, the LIFE will be conducted in person.

In Study 1 and 2, two types of secondary analysis will be conducted. First, recognising that the number of observed relapses may not be sufficient to support reliable modelling, a secondary analysis will be conducted on the prediction of *symptom burden* in the follow-up window (three epochs in Study 1, one in Study 2). Weekly symptom burden will be measured on the LIFE on 6-point scales for weekly depression and mania (see above). Second, in all studies, modelling analyses will be re-conducted using the outcome measure of self-reported Quality of Life (QoL), a patient-valued outcome. For the present project, QoL scores have the advantage of being a meaningful variable in both the interepisode cohorts of Study 1 and 2, and the highly symptomatic cohort of Study 3. The BD-specific QoL.BD [short form, 83, 84] will be collected at 13- and 26-week follow-ups in Study 1 and 2 and at the week 8 endpoint in Study 3.

*Study 3: Treatment response*

The primary outcome variable in Study 3 will be 50% reduction in MADRS or YMRS scores at 8 weeks. The prospective relationship between weekly RAR and weekly symptom assessments (MADRS, YMRS, CGI) for 21 days will be explored to investigate dynamics of RAR in relation to symptoms over these shorter timeframes.

### 2.5 Predictor variables

*Sleep and circadian rhythm disruption predictors*

First and foremost, in all three studies, predictor variables include objectively measured RAR parameters from 14 days of actigraphy (see above). In Study 1 and 2 only, to interpret actigraphy-based sleep variables, participants will also complete the Consensus Sleep Diary ^85^ across the 14 days of monitoring. The Van Someren variables Relative Amplitude, Interdaily Stability and Intradaily Variability will be considered for inclusion in all models. Beyond these commonly studied actigraphy variables, Complex System Modelling may identify novel predictive features from RAR (see below).

Given the intimate involvement of environmental light in circadian entrainment, ^86^^,^^87^ predictive models may be improved if the actigraphy signal is augmented with continuous illuminance data [see, e.g., 88, 94]. Participants in Study 1 and 2 will wear *MiEye*, a lapel device measuring melanopic and photopic illuminance, for 7 of the 14 actigraphy days. ^95^

*Clinical predictors of relapse (Study 1 and 2)*

The present project assumes that objective SCRD data from actigraphy potentially augments, rather than replaces clinical variables in the prediction of BD course. ^53^^,^^95^ Informed by existing literature, five clinical predictors will be included in modelling of RAR-based variables in Study 1 and 2 (clinical predictors will not be included in the models of Study 3 because of concerns about participant burden). Analyses will investigate the impact of, (i) BD subtype, (ii) number of past episodes (measured on the SCID-5-RV), (iii) current use of multiple mood stabilisers by self-report, (iv) residual depressive symptoms measured on the MADRS, and (v) self-reported medication adherence on the Medication Adherence Report Scale. ^53^

The decision to elevate these five predictors was informed by a critical review, ^6^ by a meta-analysis of risk of subsequent mood episodes by Radua and colleagues, ^96^ and the published study most similar in design to the present Study 1. ^69^ Each of these predictors has arisen in at least one prospective data set: BD subtype (time to relapse shorter in BD-II ^96^), number of past episodes ^6^ (iii) current use of multiple mood stabilisers, ^6^ (iv) residual depressive symptoms, ^96^ (v) medication adherence. ^6^ Other variables will be included if a clear rationale can be provided, but a subject-to-variable ratio of 7 to 1 will be preferred.

### 2.6 Modelling approaches

As noted above, it is not possible to present a detailed statistical analysis plan until the data to be analysed is clear. Here, we overview data preprocessing plans, present a power analysis for Study 1, and overview the three planned analytic approaches.

*Data preprocessing*

Two categories of missing data will be identified and managed using tailored approaches. For actigraphy data, short and scattered gaps, where the device is worn but a few data points are missing, will be imputed using forward- and backward-fill (FFill/BFill) methods, which are suitable for brief intervals. Longer and continuous gaps, arising from non-wear periods such as device charging or participant non-adherence, will be addressed using a subject-specific model that combines a circadian baseline estimating expected activity at each clock time with a recurrent neural network (RNN) that models short-term deviations from this baseline. A gating mechanism will determine the weighting between these components according to the duration of the gap, giving greater weight to the baseline for longer periods. All imputed data points will be flagged for potential exclusion in sensitivity analyses.

Missingness in clinical data will be handled according to analytic role: participants missing essential outcomes (e.g., relapse status) will be excluded from analyses requiring those outcomes. Clinical predictors and covariates will not be imputed; analyses will use available-case data. Patterns and proportions of missing data will be reported, and sensitivity analyses will assess the robustness of study findings.

*Power Analysis*

Based on the effect size identified by Ferrand et al (see above), a one standard deviation increase in the actigraphy measure increases the risk of relapse by 57% (Hazard Ratio = 1.57). Assuming a sample size of *N* = 100 participants, initially assuming zero drop-out and then 20% drop-out ( *N* = 80) we used simulation to calculate power estimates based on different Hazard Rate assumptions for a Cox Survival Analysis in Study 1. First, a sample of *N* = 100 provides 90.6% power to detect a 57% increase in the relative risk of relapse while a sample size of *N* = 80 provides 75.6% power to detect such an increase. Second, a sample of *N* = 100 provides 83.0% power to detect a 49% increase in the relative risk of relapse while a sample size of *N* = 80 provides 83.8% power to detect a 65% increase in the relative risk of relapse. This suggests that even with a 20% dropout the study is nearly sufficiently powered to detect the expected relative risk of relapse (Hazard Ratio = 1.57) and powered to detect a slightly higher relative risk of relapse (Hazard Ratio = 1.65). Note also that the selected sample sizes of *N* = 100 people with BD in Study 1, 50 in Study 2 (25 BD, 25 matched controls), and 45 in Study 3 (15 manic and 30 depressed) were pragmatic maxima. Recruitment beyond these pragmatic maxima will continue if resourcing permits.

*Cox Survival Analysis*

Cox Survival Analysis will be employed with Elastic Net ^97^ to determine variables to include. Relative Amplitude, Intradaily Variability, Interdaily Stability will be included as predictors in a random effects or frailty model of survival time to relapse (across the three epochs of Study 1 and the single epoch of Study 2). Initial time to first episode analysis: Proportional Hazards (Cox) regression with the five clinical covariates listed above. For the modelling of recurrent episodes a Prentice-Williams-Prentice (PWP) model will be used rather than a frailty model because the PWP is appropriate when only a small number of events is likely. ^98^

To model the relationship between the actigraphy signal and time to first episode, a two-stage joint survival model will be used. These models analyse time-to-event outcomes that may be linked to biomarkers that are repeatedly measured. A two-stage joint survival model analyses time-to-event outcomes influenced by a longitudinal, time-varying biomarker by fitting two separate models in sequence: a longitudinal model for the biomarker and a survival model for the event. This is a computationally efficient alternative to fully joint models, which can be very time-consuming to fit. The main drawback is that standard two-stage methods can disregard the uncertainty from the first stage’s estimates when they are used in the second, though advanced methods use techniques like multiple imputation to account for this. ^99^

Survival analysis has shown that the probability of recovery is significantly lower from major depressive episodes compared to mania or hypomania. Recovery is also less likely from cycling episodes (switching between poles without a recovery period). This means that, ideally, manic and depressive episodes should be analysed together (suggesting a joint survival model for these two types of episode), but it is unclear if we will have sufficient data for this. Decisions will be made on review of the complete dataset and reported in the statistical analysis plan (forthcoming).

*Dynamic Bayesian Network Analysis*

Network analysis is a variant of machine learning designed to illuminate the architecture of complex systems. ^97^ In this project, network analysis is used to model potential causal pathways amongst sleep and circadian variables in the prediction of relapse. The analysis generates a graphical representation of nodes (individual characteristics and variables) and edges (connections and links among nodes). Location of nodes and strength/valence of links between nodes represent the specific association between two nodes while controlling for all other variables.

Scott and colleagues recently undertook a pilot study in a sample of young people with emerging BD and produced a network model of RAR variables relative to each other and to phenotypic features of BD. ^33^ This study found further support for the importance of robust circadian rhythmicity as measured in Relative Amplitude (see above). However, the authors highlight significant uncertainties in their modelling outcomes, concluding that “… whilst all research findings require replication and confirmation, we stress that this is particularly important in this emerging field of research” (p. 225).

The network analysis of Scott et al. was developed using cross-sectional data, while the present prospective project will permit exploration of how RAR variables change in relation to each other and to other BD symptoms over time. To model these temporal dependencies, Dynamic Bayesian Network Analysis will be applied. Dynamic Bayesian Networks are well suited to representing probabilistic relationships that evolve dynamically and can therefore capture time-dependent causal pathways among RAR, sleep, and clinical variables [see, e.g., 100, 101]. In addition to Dynamic Bayesian Network Analysis, other machine learning frameworks that complement causal inference with predictive performance will be explored. Specifically, ensemble-based models such as Gradient Boosting Machines and Random Forests, and regularised regression approaches such as LASSO or Elastic Net, will be considered to identify combinations of RAR features that best predict relapse or treatment outcomes.

*Hadaeghi’s Complex System Model of BD*

A key clinical challenge in predicting the course of BD is the condition’s dramatic state changes, most commonly discussed in relation to switching. ^102^ It has been proposed that switching and other nonlinear patterns in the course of BD are best understood from a complex system viewpoint, as clinically critical transitions in state space. ^68^^,^^103^ From this perspective, relapse into a new episode and recovery from an acute episode both represent state transitions at which interacting biological and behavioural processes lose or regain stability, and the system shifts to a new state.

Importantly for the theoretical coherence of the present SCRD-grounded project, Hadaeghi’s Complex System Model conceptualises BD as the interaction between two coupled subsystems: the circadian pacemaker and the frontal–limbic system, which governs emotional regulation and behavioural activation. Feedback between these subsystems can maintain stable oscillations (corresponding to episode stability) or, under certain conditions, produce unstable dynamics manifesting as manic or depressive episodes. Endogenous perturbations (e.g., sleep–wake irregularity) or exogenous influences (e.g., psychosocial stress or treatment changes) can shift the system toward instability.

In this project, we implement a complex system-inspired predictive model that adapts the principles of Hadaeghi’s nonlinear dynamical framework to empirical actigraphy data. Rather than directly solving the system of equations, we estimate proxy indicators of system stability from the actigraphy signal. These indicators, which constitute the model’s features, will serve as a bridge between the abstract dynamical system and observed behavioural data.

The extracted indicators will include statistical, time–frequency, and nonlinear features derived from the activity time series and computed over sliding temporal windows, yielding time-varying feature trajectories that reflect changes in system stability. Statistical features (e.g., mean, variance, skewness) will describe overall activity levels; time–frequency features (e.g., spectral power, rhythm amplitude, phase stability) will capture periodicity and circadian patterns; and nonlinear features (e.g., sample entropy, fractal dimension, Lyapunov exponent, correlation dimension, detrended fluctuation analysis) will quantify the complexity and predictability of rest–activity dynamics.

The predictive framework will be implemented using nonlinear dynamic modelling approaches, such as recurrent neural networks (RNNs) or nonlinear autoregressive models, which model sequences of time-indexed feature vectors and are, therefore, well-suited to capturing feedback relationships and temporal dependencies in evolving system dynamics. These models do not explicitly recover the underlying dynamical equations but are designed to learn temporal dependencies in the observed data. These data-driven models are conceptually aligned with Hadaeghi’s formulation of BD as an interacting dynamical system.

The model will be trained to identify patterns and trajectories in these features that precede critical transitions in system stability. In Studies 1 and 2, these transitions will correspond to relapse events among interepisode participants, while in Study 3 they will represent treatment response during acute manic or depressive episodes.

*Input and outcome variables for modelling*

The primary input data for all predictive modelling in Studies 1, 2 and 3 will consist of 14 days actigraphy time-series and derived features extracted through both time-domain and frequency-domain analyses. Feature extraction will include conventional RAR metrics such as the Van Someren variables (Relative Amplitude, Intradaily Variability, and Interdaily Stability), sleep–wake regularity, rhythm phase, and fragmentation, as well as signal-based features obtained from Fourier and wavelet analyses that capture periodicity, spectral power distribution, and nonstationary temporal patterns. Together, these actigraphy-derived metrics will form the principal set of predictors for analyses based on Cox Survival Analysis, Dynamic Bayesian Networks, Hadaeghi’s Complex System Model of BD and other machine learning approaches to be explored.

Auxiliary variables, including demographic and clinical data, will be used to support feature interpretation and to define model outcomes. In Studies 1 and 2, the primary outcome will be relapse, defined as the onset of a new depressive, manic or hypomanic episode meeting DSM-5 diagnostic criteria. Relapse will be identified through structured follow-up interviews using the LIFE (above). Time to first relapse will also be analysed as a survival outcome. In Study 3, the primary outcome will be treatment response, defined as at least a 50% reduction in baseline MADRS or YMRS scores at 8-week endpoint.

These outcome variables will be used to label actigraphy epochs and to train, validate, and compare predictive models of relapse risk and treatment response.

### 2.7 Procedures

*Project registration*

The three-study project as detailed here was registered with Open Science Foundation on 24 ^th^ April 2025 ( https://doi.org/10.17605/OSF.IO/UABNJ).

*Ethics*

All three studies have ethics approval. Ethics approval for Study 1 was provided by Swinburne University of Technology Human Research Ethics Committee (SUHREC/2025/8127). Study 2 has received biosafety approval from Institutional Biosafety Committee (IBSC) of Indian Institute of Technology Hyderabad and Institutional Ethics Committees of the Medical College Kolkata and Indian Institute of Technology Hyderabad (ITH/IEC/2025/01/2). Study 3 received approval from the New Zealand Health and Disability Ethics Committee (HDEC, 2024 EXP 22105). Scientific findings will be reported according to the Strengthening the Reporting of OBservational studies in Epidemiology (STROBE) guidelines. ^104^ The project sponsor is Swinburne University of Technology.

*Risk management approach Study 1 and Study 2*

In all studies, treatment as usual continues throughout. Risk management for Study 1 and 2 has been developed through our experience with other online interventions and websites for BD [e.g., 105– 108], and through consultation with our lived experience colleagues. Key principles of the approach include protection of autonomy where possible, explicit assignment of risk management to participants and their local networks, and advance planning for crises. ^109^ Statements of informed consent highlight, (i) the project cannot and does not provide any therapy, treatment, support, or emergency management, (ii) the participant and their local networks of care remain central in their own safety and well-being throughout. The project landing page reiterates that no crisis support can be obtained through the site, but includes a ‘crisis’ tab, which provides suicide hotline phone numbers worldwide ( *unsuicide.wikispaces.com* ).

To minimise the impact of any adverse events in Study 1 and 2, being under the ongoing care of a nominated medical practitioner is an inclusion criterion. In Study 1, once participants consent, an email will be sent to their practitioner noting that the patient has enrolled in the study, that the researchers are not taking over their care, and that the provider will potentially be contacted should the researchers become aware of crisis or risk. At each follow-up assessment interview, clinicians’ contact details will be updated if required and suicidality checked by re-administering the CSSRS.

Four specific risk management processes of Studies 1 and 2 are noteworthy. First, active suicidality (operationalised as CSSRS of 3, 4 or 5) is an exclusion criterion, as is reason to think that participation might compromise care. People who are at baseline or become ineligible due to active suicidality will be provided with support and referred back to their clinician. On a case-by-case basis, people excluded because of active suicidality may be invited to recontact researchers once treatment and symptom stability are in place.

Second, as noted above, meeting MADRS or YMRS episode criteria (> 12 and > 8, respectively) is an exclusion criterion at baseline. Following a protocol we have used previously to balance risk management with lived experience empowerment, ^20^ potential participants who are in an episode at baseline, but meet all other criteria for the study will be offered the option of being recontacted in 4 weeks to see if they would like to repeat the eligibility assessment (‘re-assessed’).

Third, as part of ensuring that participation does not negatively impact participants’ treatment or recovery, people showing high level of symptoms at follow-up assessment interviews (i.e., meeting episode criteria of MADRS > 12 or YMRS > 8) will be reviewed by the research team (including the study clinician) post-interview. Actions arising from this review will include sharing with the participant that they currently meet episode criteria and encouraging them to organise an appointment with their clinician. On a case-by-case basis, actions may also include, (i) offering the participant to pause their participation for 4 weeks and be recontacted to repeat the eligibility assessment, or (ii) informing the participant that they are ineligible to continue on the basis of symptom burden/complexity (this potentially difficult news would be delivered by the project manager or a clinician as required, and may include identifying other ways the individual can participate in other aspects of the team’s program of research and community outreach).

Finally, management of acute suicidality in Study 1 and Study 2 again follows principles developed in our earlier research. ^20^ If the CSSRS indicates active suicidality at baseline or follow-up interview, research assistants will use a warm handover procedure via teleconference to escalate the participant to a clinical psychologist (Study 1) or psychiatrist (Study 2). The clinician will then explore risk further, if necessary developing a safety plan, which may include contacting the participant’s clinician or emergency services (participants consent to this specific practice). Staff training (and an associated manual) emphasises issues of self-harm, suicidality, mania and depression symptom severity scores in this population, and use of the CSSRS interview to assess for active suicidality. Training will prioritise procedures for providing feedback in a clinically sensitive manner, and escalating risk to the on-call clinician. At all times patient welfare is prioritised over collection of data.

*Risk management Study 3*

All participants in Study 3 will be receiving active management under a specialist mental health team. Everyone receiving care under the specialist mental health services is required to have a treatment plan which includes a risk management plan. The nurse or research assistant conducting interviews will be aware of the individual’s existing treatment plan and will refer to this if required.

*Data Security*

In Study 1, data will be gathered using REDCap, a highly secure web-based research data collection system. REDCap provides several data security features, including secure passwords required for staff log-in, well-defined levels of data access based on a staff member’s role in the project, and data storage on the password-protected OneDrive of the lead organisation (Swinburne University of Technology, henceforth ‘Swinburne’). In Study 2 and 3, data will initially be collected using paper-pen and subsequently transferred to REDCap. REDCap system design will be standardised across studies where possible to facilitate merging of the data for WP4. An additional separate REDCap system will be created for the genetic and metabolomic data of Study 2.

In Study 1, video contact with participants will be conducted by Swinburne Zoom accounts or Teams accounts; participants have the option to turn off their cameras during those meetings or to request telephone interviews in place of video calls (except for the initial confirmation of identity via photo ID). In Study 2 and 3, participants will be interviewed exclusively in person. Study 3 participants who are recruited as inpatients will be interviewed in person, those recruited as outpatients will have baseline interviews in person, and follow-up interviews by phone.

Only members of the research team will have access to the data during data collection. However, deidentified data will also be released to the data safety and monitoring committee (DSMC). All data collected at each of the sites will be de-identified before transfer and uploaded as re-identifiable, password-protected files to the Swinburne OneDrive. Swinburne will hold a backup of all these data. Only members of the research team will be permitted to access these data. Data access and security arrangements will be detailed in the Participant Information Form and consented to by all participants.

A key deliverable of WP4 is a new multi-national dataset and data processing pipelines to be shared with future researchers. Data records and materials will be retained by Swinburne and archived for 20 years on Swinburne’s OneDrive. Beginning 12 months after publication, deidentified data will, on a case-by-case basis, be made available to external researchers with methodologically sound proposals that are consistent with participants’ provision of extended consent. Code for the actigraphy processing pipeline will be shared through the project’s GitHub repository server. Metabolomics data will be submitted to the Metabolights platform for sharing ( https://www.ebi.ac.uk/metabolights/index). This data repository follows globally accepted regulations.

To protect participant confidentiality, privacy standards that match or exceed those of all countries from which the project recruits will be maintained. Swinburne will be the data custodian of data from all sites. Upon completion of the project and acceptance of its primary scientific outputs, de-identified data, data analysis scripts, statistical analysis plan, informed consent, publicly available (non-copyrighted) measures will be shared on the Open Science Foundation.

### 2.8 Project governance

**Figure 4.**
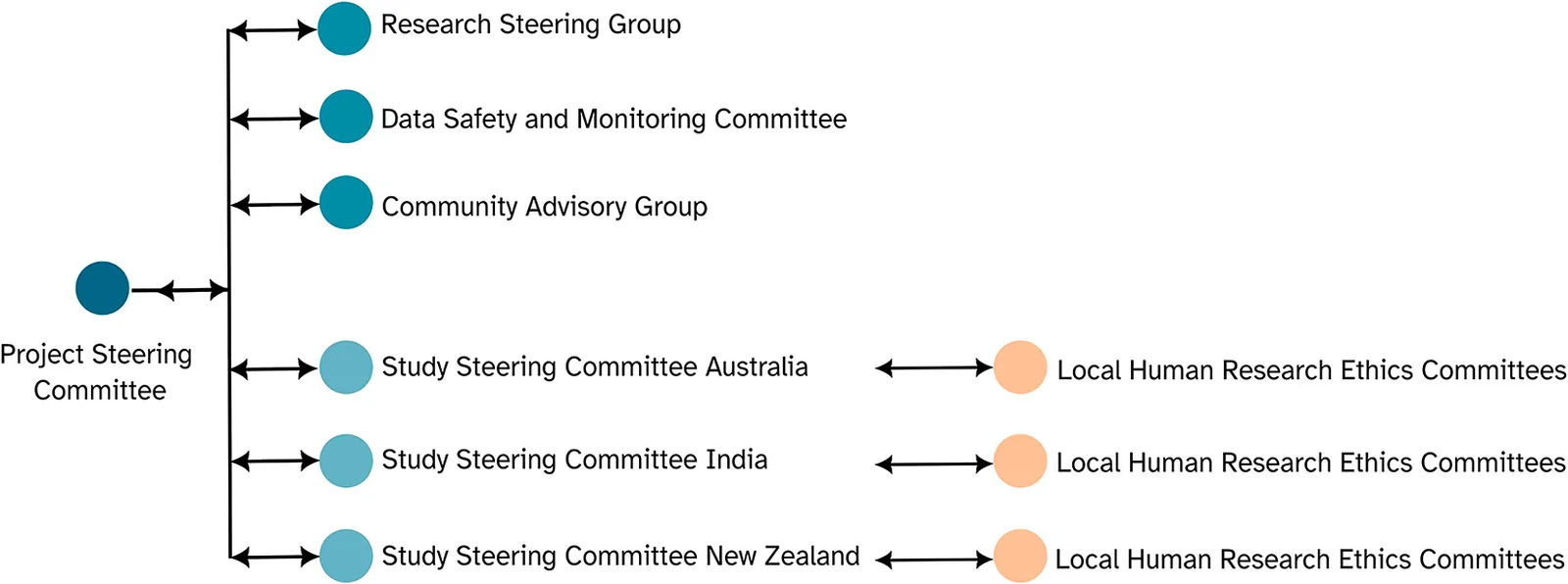
Project governance structure.

A Project Steering Committee meets monthly to oversee project management, and scientific, ethical, legal, and societal aspects of the project (see Figure 4). Members of the Committee include the Project Manager (Secretary), the individual study leads for the three empirical studies (WP1 = GM; WP2 = SR; WP3 = RP), leads of the data synthesis and modelling work (JS, FH, DM) and at least one LE collaborator. Local study steering committees will have day-to-day responsibility for the three empirical studies.

A research steering group (RSG) has been constituted to keep the funder informed about project progress. Membership of the RSG includes the Principal Investigator (GM); one representative of the Swinburne’s technology transfer office, and at least one representative or nominee of Wellcome. The RSG meets annually and receives project reports every 6 months.

The DSMC is led by an independent chair. The committee charter empowers the committee to recommend removing participants from a study or recommend that a study be modified or stopped. Membership includes an independent statistician, and an early career researcher with experience in circadian rhythms, mood disorders, and prospective study designs. The committee reviewed all aspects of the protocol before data collection began and will be informed of the progress of the three empirical studies, including enrolment, attrition, outcome variables, adverse events, and planned protocol changes. The DSMC will review individual safety reports, aggregate event rates, data quality and security, protocol adherence, participant recruitment and retention rates (including participant burden issues). The DSMC will review participants’ formal outcome assessments and any adverse events. Serious adverse events (participant death, hospital admissions, episodes of crisis assessment team treatment, attempted suicide, violent incidents leading to police involvement) will be recorded systematically. The DSMC will review data of the first 5 baseline assessments for Study 1, 2, and 3 to exclude systematic adverse events associated with participation. The DSMC will meet twice during the first year of data collection, and annually thereafter or as required. Reports of the DSMC will be forwarded to the RSG (and where required, local HRECs).

Finally, a community advisory group (CAG) has been developed to provide input to the Project Steering Committee. Chaired by one of our LE collaborators (SL), the CAG will meet three times throughout the project with provisional agendas set for each session (e.g., Yr 1: project protocol, participant burden of assessments and recruitment, Yr 3: recruitment and retention, Yr 5: interpreting and disseminating findings, next steps, participants’ views of the findings). The Project Steering Committee will provide feedback to the CAG on how their input did or did not affect project plans and why.

### 2.9 Timelines and Deliverables

All four work packages will be conducted in parallel (see Table 1). Careful attention has been paid to staffing of the work packages to ensure feasible workloads across researchers and project staff. As of July 2026, data collection has commenced for all three studies, and meetings of the DSMC, CAG and RSG have been held as scheduled.

**Table 1.** Gantt chart of project activities.

Task	Year 1				Year 2				Year 3				Year 4				Year 5			
	1	2	3	4	1	2	3	4	1	2	3	4	1	2	3	4	1	2	3	4
**Project oversight**																				
Governance structures																				
Inter-institution contracts																				
Project-level protocol paper																				
Recruit project manager																				
**Work Package 1 Australia**																				
Ethics approval																				
Recruit and train study staff																				
Build local data base																				
Recruitment																				
Data collection																				
Local data cleaning and analysis																				
**Work Package 2 India**																				
Ethics approval																				
Build local data base																				
Recruitment																				
Data collection																				
Local data cleaning and analysis																				
**Work Package 3 New Zealand**																				
Ethics approval																				
Build local data base																				
Recruitment																				
Data collection																				
Local data cleaning and analysis																				
**Work Package 4**																				
Theoretical work																				
Data pipeline development																				
Coordinate data transfer to Swinburne																				
Cross-validation of findings																				
Data synthesis																				
Model-building																				
**Dissemination**																				
Draft publications																				
Prepare conference submissions																				
Report findings to participants, seek feedback																				

Note: Formal project commencement date: 14th July 2024.

## 3. Discussion

The Tipping Point project is conducted at a time of growing scientific interest in SCRD, wearables and computational approaches in mood disorders. A key driver of this interest is the potential for clinically translatable tools. The present project takes up this challenge and has two linked aims. The primary aim is to develop a relapse risk algorithm for people living with BD. The algorithm has translational potential, because actigraph technology is non-invasive, cheap and acceptable to people with BD. The project’s secondary aim is to increase confidence in the derived RAR algorithm by improving mechanistic understanding of RAR-measured SCRD involvement in BD. The underlying mechanisms of RAR-based prediction of relapse risk will be illuminated by, (i) testing the association between RAR parameters and molecular biological measures of circadian function, and (ii) investigating whether a complex systems model of relapse risk can also predict another state transition in BD – recovery from an acute episode.

The project has several strengths. First, it is highly interdisciplinary, with team members bringing expertise in psychology, psychiatry, statistics, computational biology, molecular biology, and behavioural analyses. Second, our long-standing lived experience collaborators have been influential in developing the grant application, the present protocol, and remain active in data collection, governance, and translation of findings. Growing from our work in participatory research [e.g., 70, 110, 111], an assumption of the project is that participants are also collaborators in, rather than subjects of, the research: participants are properly remunerated for their time and will be consulted on project findings to inform next steps. Third, the project compares data from Australian and Indian samples: whilst the latter sample is of modest size, the project is an important first attempt to see if modelling findings replicate beyond high-income countries. ^112^ Finally, the person-days of Study 1 ( *N* = 14 days X 3 epochs X 100 participants = up to 4,200 actigraphy days; *N* = 7 days X 26 weeks X 3 epochs X 100 participants = up to 54,600 follow-up days) make it to our knowledge the largest prospective investigation of actigraphic predictors of BD course to date.

Nonetheless, the most important limitation of the project is the relatively small number of data points for computational modelling. ^113^ For example, the total number of participants ( *N* = 100 at baseline) for modelling relapse events in Study 1 is small: A commonly cited prospective study estimates individuals with BD I experience 0.4 new episodes per annum on average, ^114^ so we estimate only approximately 60 relapses will be observed. Replication of the project’s findings in unseen data sets is therefore essential. We are in discussion with teams worldwide for data sharing to support these tests. For example, we have had discussions with, (i) another Wellcome-funded project collecting RAR data via actigraphy in a large prospective sample of BD https://wellcome.org/research-funding/funding-portfolio/funded-grants/ambient-and-passive-collection-sleep-and-circadian (ii) the BD2 consortium which is collecting RAR via Fitbit in a large prospective study of people diagnosed with BD-I https://www.bipolardiscoveries.org/our-work/integrated-network/.

Several strategies are embedded to mitigate the sample size constraint and maximise the project’s value. First, the high-resolution nature of actigraphy (> one million data points per epoch per participant) is leveraged to support modelling within-subject dynamics. Second, Study 1 collects three distinct monitoring epochs per participant, increasing the number of analytic events available for intra-individual analyses. Finally, the inclusion of the simple, widely used Van Someren variables provides a crucial anchor to large cross-sectional datasets (e.g., UK Biobank), allowing for contextualisation and comparison of our prospective findings.

The project’s findings may have several implications. First and foremost, if a predictive algorithm of relapse risk can be identified and subsequently replicated in independent samples, the project will have made a step towards an automated forecasting tool for BD [as being trialled for people living with epilepsy, 102]. Beyond providing early warning advice, such an indicator may play a more positive role in BD management, by minimising treatment burden. For example, such a tool could play a part in deciding who may not require ‘infinite maintenance treatment’ for their BD. ^115^

Secondly, the project will generate an entirely novel network model of the relationship between RAR, sleep and circadian parameters as predictors of relapse in BD. As argued in a recent expert report, mechanistic understanding of SCRD in mental health is lacking. ^35^ The actigraphy signal alone is not a direct measure of biological mechanisms, but the use here of multiple computational frameworks and the triangulation provided by three studies (including investigations of novel mechanistic questions in Study 2 and 3) will support parsing out these interwoven signals. ^54^

A further deliverable is a large open science database and analysis pipelines, to be shared with future researchers who wish to apply different statistical and analytic techniques. In close collaboration with other research groups funded by the Wellcome Trust SCRD funding scheme, the research team will ensure that the multi-national dataset and processing pipelines meet FAIR criteria. ^116^

## 4. Conclusion

The present paper introduces the rationale for, and four work packages of, a 5-year international project designed to incrementally advance understanding of RAR as a predictor of mood dynamics in BD. The project was designed to be the obvious next step in a rapidly expanding research area. The multi-study, multi-disciplinary design, which notably includes a focus on building new team capacity and involving a lower-middle income country site, is our strategic response to the complexity of these questions. We aim to make a thoughtful, exploratory contribution by systematically charting what is known, what is not, and where the true potential lies.

## Authors’ contributions

Murray drafted the paper based on an original grant application developed by Murray, Scott, Ray, Porter, Hadaeghi, Choi, Lapsley, and Meyer. Meyer, Tremain, Nguyen (Australia), Ray, Saha (India), Porter, and Tennant (New Zealand) provided detailed input into individual study designs. Meyer developed the survival analysis and power analysis for Study 1 and had particular input into these sections. Scott developed the Dynamic Bayesian Network Analysis and had particular input into these sections. Hadaeghi developed the complex system analysis and had particular input into these sections. Ray developed the molecular analysis protocol and had particular input into these sections. Choi, Thwaites, and Lapsley developed the planned Community Advisory Group. All other authors provided suggestions and had significant intellectual input into the final draft.

## Ethical considerations

Study 1 and Study 2 participants will provide written and informed consent at study sign-up via the online sign-up form. Participants will then reconfirm their written and informed consent with the research assistants prior to the commencement of the baseline assessment. Study 3 participants will provide written and informed consent prior to the commencement of the study interview after their capacity to provide consent has been confirmed by an independent senior clinician. Study 3 participants will be encouraged to use supported decision making with a trusted family member or friend and will have their consent revisited during and after their time in the study. Ethical approval for Study 1 was provided by Swinburne University of Technology Human Research Ethics Committee on 11/11/2024 (SUHREC/2025/8127). Ethical approval for Study 2 was provided by the Indian Institute of Technology Hyderabad Institutional Ethics Committee (IEC) on 05/05/2025 (IITH/IEC/2025/01/21). Study 2 has also received biosafety approval from the Institutional Biosafety Committee (IBSC) of the Indian Institute of Technology Hyderabad on 30/12/2024 (IBSC/IITH/2024/Meeting-II/05). Study 3 obtained ethical approval (2025 EXP 22105), from the New Zealand Health and Disability Ethics Committee (HDEC) on 9/06/2025.

## Footnotes

^1^

Consensus is emerging that circadian rhythm disruption is not specific to BD [ 15] and is potentially a transdiagnostic mental health risk factor. ^12^^,^^65^

^2^
These papers include additional local activities (e.g., embedded PhD projects) that expand beyond the project described here.

^3^
In Study 1 and 2, participants are not required to be strictly euthymic or to have maintained a stable mood for any period of time. Participants are required to not be experiencing an episode of BD at baseline, but a level of residual symptoms is accepted to maximise generalisability of the findings.

^4^
The baseline exclusion criterion of current episode status in Study 1 and 2 (MADRS > 12 or YMRS > 8) is not the outcome measure of the occurrence of an episode relapse event. As discussed below, the retrospective Longitudinal Interview Follow-up Evaluation (LIFE) interview ^75^ is used to assess occurrence of a new episode in Study 1 and Study 2.

^5^
Some participants may experience multiple distinct relapse events: decisions about modelling these outcomes will be made after preliminary analyses of LIFE weekly ratings are used to characterise individual patterns of relapse, recurrence and remission [informed by, 93].

## References

[1] Whiteford HA Degenhardt L Rehm J : Global burden of disease attributable to mental and substance use disorders: findings from the Global Burden of Disease Study 2010. *Lancet.* 2013;382(9904):1575–1586. 10.1016/S0140-6736(13)61611-6 23993280

[2] Bergeson JG Kalsekar I Jing Y : Medical care costs and hospitalization in patients with bipolar disorder treated with atypical antipsychotics. *Am Health Drug Benefits. * 2012;5(6):379–386. 24991334 PMC4031693

[3] Chen M Fitzgerald HM Madera JJ : Functional outcome assessment in bipolar disorder: A systematic literature review. *Bipolar Disord.* 2019;21(3):194–214. 10.1111/bdi.12775 30887632 PMC6593429

[4] KPMG: *Paving the way for mental health: The economics of optimal pathways to care.* 2014. Reference Source

[5] Duffy A Grof P : Longitudinal studies of bipolar patients and their families: translating findings to advance individualized risk prediction, treatment and research. *Int J Bipolar Disord.* 2024;12(1):12. 10.1186/s40345-024-00333-y 38609722 PMC11014837

[6] Etain B Bellivier F Olié E : Clinical predictors of recurrences in bipolar disorders type 1 and 2: A FACE-BD longitudinal study. *J Psychiatr Res.* 2021;134:129–137. 10.1016/j.jpsychires.2020.12.041 33385631

[7] Colombo F Calesella F Mazza MG : Machine learning approaches for prediction of bipolar disorder based on biological, clinical and neuropsychological markers: A systematic review and meta-analysis. *Neurosci Biobehav Rev.* 2022;135:104552. 10.1016/j.neubiorev.2022.104552 35120970

[8] Lipschitz JM Lin S Saghafian S : Digital phenotyping in bipolar disorder: Using longitudinal Fitbit data and personalized machine learning to predict mood symptomatology. *Acta Psychiatrica Scandinavica.* 2025;151(3):434–447. 10.1111/acps.13765 39397313 PMC13552231

[9] Esaki Y Obayashi K Saeki K : Association between circadian activity rhythms and mood episode relapse in bipolar disorder: a 12-month prospective cohort study. *Translational Psychiatry.* 2021;11(1):525. 10.1038/s41398-021-01652-9 34645802 PMC8514471

[10] Fried EI Proppert RKK Rieble CL : Building an Early Warning System for Depression: Rationale, Objectives, and Methods of the WARN-D Study. *Clin Psychol Eur.* 2023;5(3):e10075. 10.32872/cpe.10075 38356901 PMC10863640

[11] Hickie IB Crouse JJ : Sleep and circadian rhythm disturbances: plausible pathways to major mental disorders? *World Psychiatry.* 2024;23(1):150–151. 10.1002/wps.21154 38214638 PMC10786001

[12] Meyer N Lok R Schmidt C : The sleep-circadian interface: A window into mental disorders. *Proc Natl Acad Sci USA.* 2024;121(9):e2214756121. 10.1073/pnas.2214756121 38394243 PMC10907245

[13] Scott J Etain B Miklowitz D : A systematic review and meta-analysis of sleep and circadian rhythms disturbances in individuals at high-risk of developing or with early onset of bipolar disorders. *Neurosci Biobehav Rev.* 2022;135:104585. 10.1016/j.neubiorev.2022.104585 35182537 PMC8957543

[14] Scott J Hennion V Meyrel M : An ecological study of objective rest-activity markers of lithium response in bipolar-I-disorder. *Psychol Med.* 2022;52(12):2281–2289. 10.1017/S0033291720004171 33183364

[15] McCarthy MJ Gottlieb JF Gonzalez R : Neurobiological and behavioral mechanisms of circadian rhythm disruption in bipolar disorder: A critical multi-disciplinary literature review and agenda for future research from the ISBD task force on chronobiology. *Bipolar Disord.* 2022;24(3):232–263. 10.1111/bdi.13165 34850507 PMC9149148

[16] Panchal P de Queiroz Campos G Goldman DA : *Toward a Digital Future in Bipolar Disorder Assessment: A Systematic Review of Disruptions in the Rest-Activity Cycle as Measured by Actigraphy.* Frontiers. *Psychiatry.* 2022;13:780726. 10.3389/fpsyt.2022.780726 35677875 PMC9167949

[17] Bullock B Judd FK Murray G : Using actigraphy to monitor sleep-wake patterns in bipolar disorder - A case study. *Clin Psychol Forum.* 2014;1:37–41. 10.53841/bpscpf.2014.1.253.37

[18] Jakobsen P Côté-Allard U Riegler MA : Early warning signals observed in motor activity preceding mood state change in bipolar disorder. *Bipolar Disorders.* 2024;26(5):468–478. 10.1111/bdi.13430 38639725

[19] Hadaeghi F Hashemi Golpayegani MR Jafari S : Toward a complex system understanding of bipolar disorder: A chaotic model of abnormal circadian activity rhythms in euthymic bipolar disorder. *Aust N Z J Psychiatry.* 2016;50(8):783–792. 10.1177/0004867416642022 27164924

[20] Foster RG : Sleep, circadian rhythms and health. *Interface Focus.* 2020;10(3):20190098. 10.1098/rsfs.2019.0098 32382406 PMC7202392

[21] Jagannath A Taylor L Wakaf Z : The genetics of circadian rhythms, sleep and health. *Hum Mol Genet.* 2017;26(R2):R128–r138. 10.1093/hmg/ddx240 28977444 PMC5886477

[22] Takahashi JS : Transcriptional architecture of the mammalian circadian clock. *Nat Rev Genet. * 2017;18(3):164–179. 10.1038/nrg.2016.150 27990019 PMC5501165

[23] Laloum D Robinson-Rechavi M : Methods detecting rhythmic gene expression are biologically relevant only for strong signal. *PLoS Comput Biol.* 2020;16(3):e1007666. 10.1371/journal.pcbi.1007666 32182235 PMC7100990

[24] Yang MY Yang WC Lin PM : Altered expression of circadian clock genes in human chronic myeloid leukemia. *J Biol Rhythms.* 2011;26(2):136–148. 10.1177/0748730410395527 21454294

[25] Webb WB : Sleep as a Biological Rhythm: A Historical Review. *Sleep.* 1994;17(2):188–194. 10.1093/sleep/17.2.188 8036374

[26] Borbély AA Daan S Wirz-Justice A : The two-process model of sleep regulation: a reappraisal. *J Sleep Res.* 2016;25:131–143. 10.1111/jsr.12371 26762182

[27] Yousefzadehfard Y Wechsler B DeLorenzo C : Human circadian rhythm studies: Practical guidelines for inclusion/exclusion criteria and protocol. *Neurobiol Sleep Circadian Rhythms.* 2022;13:100080. 10.1016/j.nbscr.2022.100080 35989718 PMC9382328

[28] American Psychiatric Association: Text Revision of the diagnostic and statistical manual of mental disorders.Fifth edition: DSM-5-TR. 5th ed. Washington, DC: Author;2022;1120. Reference Source

[29] Gottlieb JF Benedetti F Geoffroy PA : The chronotherapeutic treatment of bipolar disorders: A systematic review and practice recommendations from the ISBD task force on chronotherapy and chronobiology. *Bipolar Disord.* 2019;21(8):741–773. 10.1111/bdi.12847 31609530

[30] Logan RW McClung CA : Rhythms of life: circadian disruption and brain disorders across the lifespan. *Nat Rev Neurosci.* 2019;20(1):49–65. 10.1038/s41583-018-0088-y 30459365 PMC6338075

[31] Lyall LM Wyse CA Graham N : Association of disrupted circadian rhythmicity with mood disorders, subjective wellbeing, and cognitive function: a cross-sectional study of 91 105 participants from the UK Biobank. *Lancet Psychiatry.* 2018;5(6):507–514. 10.1016/S2215-0366(18)30139-1 29776774

[32] McCarthy MJ : Missing a beat: assessment of circadian rhythm abnormalities in bipolar disorder in the genomic era. *Psychiatr Genet.* 2019;29(2):29–36. 10.1097/YPG.0000000000000215 30516584

[33] Scott J Etain B Grierson A : A network analysis of rest-activity rhythms in young people with emerging bipolar disorders. *J Affect Disord.* 2022;305:220–226. 10.1016/j.jad.2022.03.007 35288205

[34] Vitaterna MH King DP Chang AM : Mutagenesis and mapping of a mouse gene, Clock, essential for circadian behavior. *Science.* 1994;264(5159):719–725. 10.1126/science.8171325 8171325 PMC3839659

[35] Wellcome Trust: *Sleep, circadian rhythms and mental health.* London, UK: Wellcome Trust;2022:47. Reference Source

[36] Hühne A Welsh DK Landgraf D : Prospects for circadian treatment of mood disorders. *Ann Med.* 2018;50(8):637–654. 10.1080/07853890.2018.1530449 30265156

[37] McClung CA : How might circadian rhythms control mood? Let me count the ways. *Biol Psychiatry.* 2013;74(4):242–249. 10.1016/j.biopsych.2013.02.019 23558300 PMC3725187

[38] Roguski A Ritter P Smith DJ : Sensitivity to light in bipolar disorder: implications for research and clinical practice. *Br J Psychiatry.* 2024;224(5):143–146. 10.1192/bjp.2023.150 38174418 PMC7615859

[39] Bhatnagar A Murray G Ray S : Circadian biology to advance therapeutics for mood disorders. *Trends Pharmacol Sci.* 2023;44(10):689–704. 10.1016/j.tips.2023.07.008 37648611

[40] Bullock B Murray G : Reduced amplitude of the 24 hour activity rhythm: A biomarker of vulnerability to bipolar disorder? *Clin Psychol Sci. * 2014;2(1):86–96. 10.1177/2167702613490158

[41] Guglielmo R Miskowiak KW Hasler G : Evaluating endophenotypes for bipolar disorder. *Int J Bipolar Disord.* 2021;9(1):17. 10.1186/s40345-021-00220-w 34046710 PMC8160068

[42] Hwang JY Choi JW Kang SG : Comparison of the Effects of Quetiapine XR and Lithium Monotherapy on Actigraphy-Measured Circadian Parameters in Patients With Bipolar II Depression. *J Clin Psychopharmacol.* 2017;37(3):351–354. 10.1097/JCP.0000000000000699 28328790

[43] Patterson MR Nunes AAS Gerstel D : 40 years of actigraphy in sleep medicine and current state of the art algorithms. *NPJ Digit Med. * 2023;6:51. 10.1038/s41746-023-00802-1 36964203 PMC10039037

[44] Kaplan KA Talbot LS Gruber J : Evaluating sleep in bipolar disorder: comparison between actigraphy, polysomnography, and sleep diary. *Bipolar Disord.* 2012;14(8):870–879. 10.1111/bdi.12021 23167935 PMC3549461

[45] Ng TH Chung KF Ho FYY : Sleep–wake disturbance in interepisode bipolar disorder and high-risk individuals: A systematic review and meta-analysis. *Sleep Medicine Reviews.* 2015;20:46–58. 10.1016/j.smrv.2014.06.006 25060968

[46] American Psychiatric Association: *Diagnostic and statistical manual of mental disorders: DSM-5.* 5th ed. Washington, DC: Author;2013;947. 10.1176/appi.books.9780890425596

[47] Scott J Murray G Henry C : Activation in Bipolar Disorders: A Systematic Review. *JAMA Psychiatry.* 2017;74(2):189–196. 10.1001/jamapsychiatry.2016.3459 28002572

[48] National Advisory Mental Health Council Workgroup on Tasks and Measures for Research Domain Criteria: *Behavioral Assessment Methods for RDoC Constructs.* Bethesda, MD, USA: National Institutes of Health;2016. Reference Source

[49] Zambotti M de Goldstein C Cook J : State of the science and recommendations for using wearable technology in sleep and circadian research. *Sleep.* 2024;47(4) 10.1093/sleep/zsad325 38149978

[50] Hauser TU Skvortsova V de Choudhury M : The promise of a model-based psychiatry: building computational models of mental ill health. *Lancet Digit Health.* 2022;4(11):e816–e828. 10.1016/S2589-7500(22)00152-2 36229345 PMC9627546

[51] Lee MP Kim DW Fang Y : The real-world association between digital markers of circadian disruption and mental health risks. *NPJ Digit Med. * 2024;7(1):355. 10.1038/s41746-024-01348-6 39639100 PMC11621392

[52] Liang Y Zheng X Zeng DD : A survey on big data-driven digital phenotyping of mental health. *Inform Fusion.* 2019;52:290–307. 10.1016/j.inffus.2019.04.001

[53] Wright AGC Woods WC : Personalized Models of Psychopathology. *Annu Rev Clin Psychol. * 2020;16(1):49–74. 10.1146/annurev-clinpsy-102419-125032 32070120

[54] Murray G Gottlieb J Hidalgo MP : Measuring circadian function in bipolar disorders: Empirical and conceptual review of physiological, actigraphic, and self-report approaches. *Bipolar Disord.* 2020;22(7):693–710. 10.1111/bdi.12963 32564457

[55] Scott J Colom F Young A : An evidence map of actigraphy studies exploring longitudinal associations between rest-activity rhythms and course and outcome of bipolar disorders. *Int J Bipolar Disord.* 2020;8(1):37. 10.1186/s40345-020-00200-6 33258017 PMC7704984

[56] Fekedulegn D Andrew ME Shi M : Actigraphy-Based Assessment of Sleep Parameters. *Ann Work Expo Health.* 2020;64(4):350–367. 10.1093/annweh/wxaa007 32053169 PMC7191872

[57] Van Someren EJW : Circadian rest-activity disturbances in Alzheimer’s disease. *Biol Psychiatry.* 1996;40:259–270. 10.1016/0006-3223(95)00370-3 8871772

[58] Anikwe CV : Mobile and wearable sensors for data-driven health monitoring system: State-of-the-art and future prospect. *Expert Syst Appl.* 2022;202:117362. 10.1016/j.eswa.2022.117362

[59] Chen ZS Kulkarni P(P) Galatzer-Levy IR : Modern views of machine learning for precision psychiatry. *Patterns.* 2022;3(11):100602. 10.1016/j.patter.2022.100602 36419447 PMC9676543

[60] Abd-Alrazaq A AlSaad R Shuweihdi F : Systematic review and meta-analysis of performance of wearable artificial intelligence in detecting and predicting depression. *NPJ Digit Med.* 2023;6(1):84. 10.1038/s41746-023-00828-5 37147384 PMC10163239

[61] Tietbohl-Santos B : Chapter 7 - Precision psychiatry in bipolar disorder.In *Biomarkers in Bipolar Disorders.* Machado-Vieira R Soares JC , editors. Academic Press;2022; pp.115–124. 10.1016/B978-0-12-821398-8.00001-1

[62] Grierson AB Hickie IB Naismith SL : Circadian rhythmicity in emerging mood disorders: state or trait marker? *Int J Bipolar Disord.* 2016;4(1):3. 10.1186/s40345-015-0043-z 26763505 PMC4712181

[63] Merikangas KR Swendsen J Hickie IB : Real-time Mobile Monitoring of the Dynamic Associations Among Motor Activity, Energy, Mood, and Sleep in Adults With Bipolar Disorder. *JAMA Psychiatry.* 2019;76(2):190–198. 10.1001/jamapsychiatry.2018.3546 30540352 PMC6439734

[64] Shou H Cui L Hickie I : Dysregulation of objectively assessed 24-hour motor activity patterns as a potential marker for bipolar I disorder: results of a community-based family study. *Transl Psychiatry.* 2017;7(8):e1211–e1211. 10.1038/tp.2017.136 28892068 PMC5611716

[65] Kahawage P : The Impact of Chronotherapeutic Interventions on Mood: A Systematic Scoping Review Across Multiple Populations. *Clin Psychol Rev.* Under review.

[66] Esaki Y Obayashi K Saeki K : Circadian variability of objective sleep measures predicts the relapse of a mood episode in bipolar disorder: findings from the APPLE cohort. *Psychiatry Clin Neurosci.* 2023;77:442–448. 10.1111/pcn.13556 37092883

[67] Heath R Murray G : Multifractal dynamics of activity data in Bipolar Disorder: Towards automated early warning of manic relapse. *Fractal Geometry and Nonlinear Analysis in Medicine and Biology.* 2016;2(1):140–149. 10.15761/FGNAMB.1000124

[68] Indic P Salvatore P Maggini C : Scaling behavior of human locomotor activity amplitude: Association with bipolar disorder. *PLoS One.* 2011;6(5): e20650. 10.1371/journal.pone.0020650 21655197 PMC3105113

[69] Ferrand L Hennion V Godin O : Which Actigraphy Dimensions Predict Longitudinal Outcomes in Bipolar Disorders? *J Clin Med.* 2022;11(8):2204. 10.3390/jcm11082204 35456294 PMC9027161

[70] Michalak EE Lane K Hole R : Towards a better future for Canadians with bipolar disorder: principles and implementation of a community-based participatory research model. *Engaged Scholar J.* 2015;1(1):132–147.

[71] Porter RJ Moot W Inder ML : Validation of the Longitudinal Interval Follow-Up Evaluation for the Long-Term Measurement of Mood Symptoms in Bipolar Disorder. *Brain Sci.* 2022;12(12):1717. 10.3390/brainsci12121717 36552176 PMC9776034

[72] Earley W Durgam S Lu K : Clinically relevant response and remission outcomes in cariprazine-treated patients with bipolar I disorder. *J Affect Disord.* 2018;226:239–244. 10.1016/j.jad.2017.09.040 29017067

[73] Montgomery SA Asberg M : A new depression scale designed to be sensitive to change. *Br J Psychiatry.* 1979;134:382–389. 10.1192/bjp.134.4.382 444788

[74] Young RC Biggs JT Ziegler VE : A rating scale for mania: reliability, validity and sensitivity. *Br J Psychiatry.* 1978;133:429–435. 10.1192/bjp.133.5.429 728692

[75] Malhi GS Bell E Bassett D : The 2020 Royal Australian and New Zealand College of Psychiatrists clinical practice guidelines for mood disorders. *Aust N Z J Psychiatry.* 2021;55(1):7–117. 10.1177/0004867420979353 33353391

[76] Guy W : *Clinical global impression scale. The ECDEU assessment manual for psychopharmacology-revised volume.* Vol. Publ No ADM 76.1976: DHEW.

[77] Keller MB : The Longitudinal Interval Follow-up Evaluation: A Comprehensive Method for Assessing Outcome in Prospective Longitudinal Studies. *Arch Gen Psychiatry.* 1987;44(6):540–548. 10.1001/archpsyc.1987.01800180050009 3579500

[78] Etain B Meyrel M Hennion V : Can actigraphy be used to define lithium response dimensions in bipolar disorders? *J Affect Disord.* 2021;283:402–409. 10.1016/j.jad.2021.01.060 33581466

[79] Alloy LB Boland EM Ng TH : Low social rhythm regularity predicts first onset of bipolar spectrum disorders among at-risk individuals with reward hypersensitivity. *J Abnorm Psychol.* 2015;124(4):944–952. 10.1037/abn0000107 26595474 PMC4662076

[80] Fiedorowicz JG Merranko JA Iyengar S : Validation of the youth mood recurrences risk calculator in an adult sample with bipolar disorder. *J Affect Disord.* 2021;295:1482–1488. 10.1016/j.jad.2021.09.037 34563392

[81] Inder ML Crowe MT Luty SE : Randomized, controlled trial of Interpersonal and Social Rhythm Therapy for young people with bipolar disorder. *Bipolar Disord.* 2015;17(2):128–138. 10.1111/bdi.12273 25346391

[82] Simon GE Ludman EJ Bauer MS : Long-term Effectiveness and Cost of a Systematic Care Program for Bipolar Disorder. *Arch Gen Psychiatry.* 2006;63(5):500–508. 10.1001/archpsyc.63.5.500 16651507

[83] Michalak EE Murray G : Development of the QoL.BD: a disorder-specific scale to assess quality of life in bipolar disorder. *Bipolar Disord.* 2010;12(7):727–740. 10.1111/j.1399-5618.2010.00865.x 21040290

[84] Morton E Murray G Yatham LN : The Quality of Life in Bipolar Disorder (QoL.BD) Questionnaire a decade on – A systematic review of the measurement of condition-specific aspects of quality of life in bipolar-disorder. *J Affect Disord.* 2021;278:33–45. 10.1016/j.jad.2020.09.017 32949871

[85] Carney CE Buysse DJ Ancoli-Israel S : The consensus sleep diary: standardizing prospective sleep self-monitoring. *Sleep.* 2012;35(2):287–302. 10.5665/sleep.1642 22294820 PMC3250369

[86] Legates TA Fernandez DC Hattar S : Light as a central modulator of circadian rhythms, sleep and affect. *Nature Reviews Neuroscience.* 2014;15(7):443–454. 10.1038/nrn3743 24917305 PMC4254760

[87] Moore-Ede MC Sulzman FM Fuller CA : *The clocks that time us: physiology of the circadian timing system.* Cambridge. MA: Harvard University Press;1982. Reference Source

[88] Figueiro M Hamner R Bierman A : Comparisons of three practical field devices used to measure personal light exposures and activity levels. *Light Res Technol.* 2013;45(4):421–434. 10.1177/1477153512450453 24443644 PMC3892948

[89] First MB Williams J Karg RS : *Structured Clinical Interview for DSM-5, Research Version (SCID-5-RV).* Arlington, VA: American Psychiatric Association;2015.

[90] Taylor DJ Wilkerson AK Pruiksma KE : Reliability of the Structured Clinical Interview for DSM-5 Sleep Disorders Module. *J Clin Sleep Med.* 2018;14(3):459–464. 10.5664/jcsm.7000 29458705 PMC5837848

[91] Kumarasamy V Goodfellow N Ferron EM : Evaluating the Problem of Fraudulent Participants in Health Care Research: Multimethod Pilot Study. *JMIR Form Res.* 2024;8:e51530. 10.2196/51530 38833292 PMC11185910

[92] Posner K Brown GK Stanley B : The Columbia-Suicide Severity Rating Scale: initial validity and internal consistency findings from three multisite studies with adolescents and adults. *Am J Psychiatry.* 2011;168(12):1266–1277. 10.1176/appi.ajp.2011.10111704 22193671 PMC3893686

[93] Tohen M Frank E Bowden CL : The International Society for Bipolar Disorders (ISBD) Task Force report on the nomenclature of course and outcome in bipolar disorders. *Bipolar Disord.* 2009;11(5):453–473. 10.1111/j.1399-5618.2009.00726.x 19624385

[94] Rea MS Bierman A Figueiro MG : A new approach to understanding the impact of circadian disruption on human health. *J Circadian Rhythms.* 2008;6(1):7. 10.1186/1740-3391-6-7 18510756 PMC2430544

[95] Cain SW McGlashan EM Vidafar P : Evening home lighting adversely impacts the circadian system and sleep. *Sci Rep.* 2020;10(1):19110. 10.1038/s41598-020-75622-4 33154450 PMC7644684

[96] Radua J Grunze H Amann BL : Meta-Analysis of the Risk of Subsequent Mood Episodes in Bipolar Disorder. *Psychotherapy and Psychosomatics.* 2017;86(2):90–98. 10.1159/000449417 28183076

[97] Wu Y : Elastic net for Cox’s proportional hazards model with a solution path algorithm. *Stat Sin.* 2012;22:27–294. 10.5705/ss.2010.107 23226932 PMC3515861

[98] Amorim LD Cai J : Modelling recurrent events: a tutorial for analysis in epidemiology. *Int J Epidemiol.* 2014;44(1):324–333. 10.1093/ije/dyu222 25501468 PMC4339761

[99] Asar Ö Ritchie J Kalra PA : Joint modelling of repeated measurement and time-to-event data: an introductory tutorial. *Int J Epidemiol. * 2015;44(1):334–344. 10.1093/ije/dyu262 25604450

[100] Murphy KP : Dynamic Bayesian Networks: Representation, Inference and Learning.In *Computer Science.* Berkeley: University of California;2002. Reference Source

[101] Iorfino F Varidel M Marchant R : The temporal dependencies between social, emotional and physical health factors in young people receiving mental healthcare: a dynamic Bayesian network analysis. *Epidemiol Psychiatr Sci.* 2023;32:e56. 10.1017/S2045796023000616 37680185 PMC10539737

[102] Stirling RE Nurse ES Payne D : User experience of a seizure risk forecasting app: A mixed methods investigation. *Epilepsy Behav.* 2024;157:109876. 10.1016/j.yebeh.2024.109876 38851123 PMC13529357

[103] Hadaeghi F Hashemi Golpayegani MR Gharibzadeh S : What is the mathematical description of the treated mood pattern in bipolar disorder? *Front Comput Neurosci.* 2013;7:106. 24009579 10.3389/fncom.2013.00106 PMC3753574

[104] Vandenbroucke JP von Elm E Altman DG : Strengthening the Reporting of Observational Studies in Epidemiology (STROBE): explanation and elaboration. *PLoS Med.* 2007;4(10):e297. 10.1371/journal.pmed.0040297 17941715 PMC2020496

[105] Lauder S Chester A Castle D : Development of an online intervention for bipolar disorder. www.moodswings.net.au. *Psychol Health Med.* 2013;18(2):155–165. 10.1080/13548506.2012.689840 22712771

[106] Lauder S Berk M Castle D : www.moodswings: The highs and lows of an online intervention for bipolar disorder — preliminary findings. Aust N Z J Psychiatry. 2008;42(Supplement 3):A83–A84. Reference Source

[107] Murray G Thomas N Michalak EE : Mindfulness-based online intervention to improve quality of life in late-stage bipolar disorder: A randomized clinical trial. *J Consult Clin Psychol.* 2021;89(10):830–844. 10.1037/ccp0000684 34807658

[108] Fletcher K Lindblom K Seabrook E : Pilot Testing in the Wild: Feasibility, Acceptability, Usage Patterns, and Efficacy of an Integrated Web and Smartphone Platform for Bipolar II Disorder. *JMIR Form Res.* 2022;6(5):e32740. 10.2196/32740 35639462 PMC9198820

[109] Fletcher K Foley F Thomas N : Web-based intervention to improve quality of life in late stage bipolar disorder (ORBIT): Randomised controlled trial protocol. *BMC Psychiatry.* 2018;18(1):221. 10.1186/s12888-018-1805-9 30001704 PMC6044003

[110] Michalak EE : Commentary: Harnessing the Potential of Community-Based Participatory Research Approaches in Bipolar Disorder. *Int J Bipolar Disord.* 2016;4(4):1–9. 26856996 10.1186/s40345-016-0045-5 PMC4746206

[111] Michalak EE Suto MJ Barnes SJ : Effective self-management strategies for bipolar disorder: A community-engaged Delphi Consensus Consultation study. *J Affect Disord. * 2016;206:77–86. 10.1016/j.jad.2016.06.057 27466745

[112] Cohen JP Cao T Viviano JD : Problems in the deployment of machine-learned models in health care. *CMAJ.* 2021;193(35):E1391–e1394. 10.1503/cmaj.202066 34462316 PMC8443295

[113] Kraus B Sampathgiri K Mittal VA : Accurate machine learning prediction in psychiatry needs the right kind of information. *JAMA Psychiatry.* 2024;81(1):11–12. 10.1001/jamapsychiatry.2023.4302 38019526

[114] Solomon DA Leon AC Coryell WH : Longitudinal Course of Bipolar I Disorder: Duration of Mood Episodes. *Arch Gen Psychiatry.* 2010;67(4):339–347. 10.1001/archgenpsychiatry.2010.15 20368510 PMC3677763

[115] Dols A Kupka RW Mathijssen H : The time has come to question the infinite maintenance treatment for bipolar disorders. *Bipolar Disord.* 2024;26(5):415–417. 10.1111/bdi.13447 38736270

[116] Wilkinson MD Dumontier M Aalbersberg IJJ : The FAIR Guiding Principles for scientific data management and stewardship. *Sci Data.* 2016;3(1):160018. 10.1038/sdata.2016.18 26978244 PMC4792175

