# Supplementary material for: Forecasting the course of bipolar disorder using rest-activity rhythms: Protocol for a multi-study modelling project
Source: Wellcome Open Res. 2026 Jul 13;10:320. Originally published 2025 Jun 18. [Version 2] doi: 10.12688/wellcomeopenres.24189.2 (PMC13560521; doi:10.12688/wellcomeopenres.24189.2)
Supplement: Peer review history

# Reviewer report: Reviewer response for version 2

**DOI:** 10.21956/wellcomeopenres.28581.r161215

**Published:** 2026-07-31

**Authors:** Yeliz Aktaş

**Affiliations:** 1 İzmir Atatürk Training and Research Hospital, İzmir, Turkey

I would like to congratulate the authors on this well-conceived and innovative study protocol. The integration of actigraphy, circadian rhythm assessment, and machine learning to predict relapse in bipolar disorder addresses an important and clinically relevant research question. The multicenter, international design further strengthens the potential impact and generalizability of the study. Overall, the protocol is well presented, and I hope the following comments will help further improve the manuscript.

 1. Participants will continue receiving treatment as usual throughout follow-up, and treatment modifications are likely to occur in response to emerging prodromal symptoms. Such changes (e.g., medication adjustments, initiation of psychotherapy, hospitalization, or other clinical interventions) may alter both rest-activity rhythms and relapse risk, introducing time-varying confounding. The protocol should clarify how treatment changes during follow-up will be recorded and whether they will be incorporated into the predictive models or addressed using appropriate statistical methods for time-dependent confounding.

 2. The proposed sample size appears limited, particularly for Study 2 (25 patients with bipolar disorder) and Study 3 (45 patients), considering the complexity of the planned machine learning approaches. Although this is a study protocol, the authors should provide a stronger justification for the adequacy of these sample sizes for model development and external validation. In addition, it would be helpful to clarify the strategies that will be used to minimize overfitting and ensure model robustness, particularly given the relatively small datasets available for these analyses.

 3. The power analysis assumes a 20% attrition rate; however, the protocol does not clearly describe how participant dropout and incomplete follow-up will be managed during the 78-week follow-up period. Given the lengthy follow-up and repeated actigraphy assessments, participant attrition is likely. The authors should clarify how withdrawals, loss to follow-up, and incomplete actigraphy data will be handled in the primary analyses, as well as describe any planned retention strategies to minimize missing data.

Is the study design appropriate for the research question?

Yes

Is the rationale for, and objectives of, the study clearly described?

Yes

Are sufficient details of the methods provided to allow replication by others?

Partly

Are the datasets clearly presented in a useable and accessible format?

Not applicable

Reviewer Expertise:

psychiatry nursing, bipolar disorder, addiction, interpersonal social rhytm therapy

I confirm that I have read this submission and believe that I have an appropriate level of expertise to confirm that it is of an acceptable scientific standard, however I have significant reservations, as outlined above.

# Reviewer report: Reviewer response for version 1

**DOI:** 10.21956/wellcomeopenres.26682.r126730

**Published:** 2025-08-23

**Authors:** Danielle Novick

**Affiliations:** 1 Pittsburgh Veterans Affairs Medical Center, Pittsburgh, USA

In this manuscript, the authors detail a new project that aims to collect rest-activity rhythms (RAR) by actigraphy from individuals with bipolar disorder (BD) across two sites, one in Australia and one in India.  The project also includes four dissertation projects.

 The goals are two-fold: to develop an algorithm of relapse risk based on RAR data and to further understand RAR data in BD. 

 Overall, the manuscript is well written, clear, and concise.  The abstract is appropriate in content; there is a small typo (see below). The introduction clearly introduces relevant ideas and what is to follow.  The authors’ explanations of topics central to understanding the protocol is appropriate.  The methodology seems reasonable and sound, and statistical analyses appropriate (although, admittedly, outside the expertise of this reviewer). 

*Typo in Abstract*

**Method**

 Three prospective studies will enrol adults living with BD I or II. Study 1 (Australia) will recruit a sample of *N* = 100

 In this manuscript, the authors detail a new project that aims to collect rest-activity rhythms (RAR) by actigraphy from individuals with bipolar disorder (BD) across two sites, one in Australia and one in India.  The project also includes four dissertation projects.

 The goals are two-fold: to develop an algorithm of relapse risk based on RAR data and to further understand RAR data in BD. 

 Overall, the manuscript is well written, clear, and concise.  The abstract is appropriate in content; there is a small typo (see below). The introduction clearly introduces relevant ideas and what is to follow.  The authors’ explanations of topics central to understanding the protocol is appropriate.  The methodology seems reasonable and sound, and statistical analyses appropriate (although, admittedly, outside the expertise of this reviewer). 

*Typo in Abstract*

**Method**

 Three prospective studies will enrol adults living with BD I or II. Study 1 (Australia) will recruit a sample of *N* = 100

Is the study design appropriate for the research question?

Yes

Is the rationale for, and objectives of, the study clearly described?

Yes

Are sufficient details of the methods provided to allow replication by others?

Yes

Are the datasets clearly presented in a useable and accessible format?

Yes

Reviewer Expertise:

Bipolar disorder, Psychotherapy

I confirm that I have read this submission and believe that I have an appropriate level of expertise to confirm that it is of an acceptable scientific standard.

# Response

**Published:** 2026-02-03

**Authors:** GREG MURRAY

**Affiliations:** Centre for Mental Health and Brain Sciences, Swinburne University of Technology, Hawthorn, Victoria, Australia

REVIEWER 2 In this manuscript, the authors detail a new project that aims to collect rest-activity rhythms (RAR) by actigraphy from individuals with bipolar disorder (BD) across two sites, one in Australia and one in India.  The project also includes four dissertation projects. Note that there is also a study located in New Zealand (Study 3). We have made some additions to the text to make the rationale and aims of Study 3 clearer (please see responses to Reviewer 1). The goals are two-fold: to develop an algorithm of relapse risk based on RAR data and to further understand RAR data in BD.

 Correct.

 - Overall, the manuscript is well written, clear, and concise.  The abstract is appropriate in content; there is a small typo (see below). The introduction clearly introduces relevant ideas and what is to follow.  The authors’ explanations of topics central to understanding the protocol is appropriate.  The methodology seems reasonable and sound, and statistical analyses appropriate (although, admittedly, outside the expertise of this reviewer).

 Thank you.

 - Typo in Abstract: Three prospective studies will enrol adults living with BD I or II. Study 1 (Australia) will recruit a sample of N = 100 Not sure what the typo is here?  We have italicised the ‘N’ because it is standard practice to italicise statistical terms.

 - Is the rationale for, and objectives of, the study clearly described? YES - - Is the study design appropriate for the research question? YES - - Are sufficient details of the methods provided to allow replication by others? YES - - Are the datasets clearly presented in a useable and accessible format? YES - -        

**References** 1.         Gershon, A., et al., *Daily Actigraphy Profiles Distinguish Depressive and Interepisode States in Bipolar Disorder.* Clin Psychol Sci, 2016. **4**(4): p. 641-650. 2.         Ng, T.H., et al., *Sleep–wake disturbance in interepisode bipolar disorder and high-risk individuals: A systematic review and meta-analysis.* Sleep Medicine Reviews, 2015. **20**: p. 46-58. 3.         Fujita, A., et al., *Comparison of objective and subjective sleep parameters in patients with bipolar disorder in both euthymic and residual symptomatic periods.* J Psychiatr Res, 2021. **145**: p. 190-196. 4.         Yeom, J.W., et al., *Daily Self-Monitoring and Feedback of Circadian Rhythm Measures in Major Depression and Bipolar Disorder Using Wearable Devices and Smartphones-The Circadian Rhythm for Mood (CRM®) Trial Protocol: A Randomized Sham Controlled Double-Blind Trial.* Psychiatry Investig, 2024. **21**(8): p. 918-924. 5.         Murray, G., et al., *Online mindfulness-based intervention for late-stage bipolar disorder: pilot evidence for feasibility and effectiveness.* J Affect Disord, 2015. **178**: p. 46-51. 6.         Kahawage, P., et al., *Social Rhythm Disruption is Associated with Greater Depressive Symptoms in People with Mood Disorders: Findings from a Multinational Online Survey During COVID-19.* Can J Psychiatry, 2022. **67**(11): p. 831-840. 7.         Kim, M., et al., *Association Between Age and Sleep Quality: Findings From a Community Health Survey.* Sleep Med Res, 2021. **12**(2): p. 155-160. 8.         Hood, S. and S. Amir, *The aging clock: circadian rhythms and later life.* J Clin Invest, 2017. **127**(2): p. 437-446. 9.         Earley, W., et al., *Clinically relevant response and remission outcomes in cariprazine-treated patients with bipolar I disorder.* J Affect Disord, 2018. **226**: p. 239-244. 10.       Porter, R.J., et al., *Validation of the Longitudinal Interval Follow-Up Evaluation for the Long-Term Measurement of Mood Symptoms in Bipolar Disorder.* Brain Sciences, 2022. **12**(12): p. 1717. 11.       Fletcher, K., et al., *Web-based intervention to improve quality of life in late stage bipolar disorder (ORBIT): Randomised controlled trial protocol.* BMC Psychiatry, 2018. **18**(1): p. 221. 12.       Kim, J.A., et al., *Chronotherapeutic intervention targeting emotion regulation brain circuitry, symptoms, and suicide risk in adolescents and young adults with bipolar disorder: a pilot randomised trial.* BMJ Ment Health, 2025. **28**(1). 13.       Stromberg, A.R., et al., *Modeling and predicting mood instability in a longitudinal cohort of bipolar disorder.* Nature Mental Health, 2025. **3**(10): p. 1267-1275. 14.       Schneck, C.D., et al., *The prospective course of rapid-cycling bipolar disorder: findings from the STEP-BD.* Am J Psychiatry, 2008. **165**(3): p. 370-7; quiz 410. 15.       Fiedorowicz, J.G., et al., *Validation of the youth mood recurrences risk calculator in an adult sample with bipolar disorder.* J Affect Disord, 2021. **295**: p. 1482-1488. 16.       Etain, B., et al., *Clinical predictors of recurrences in bipolar disorders type 1 and 2: A FACE-BD longitudinal study.* J Psychiatr Res, 2021. **134**: p. 129-137. 17.       Uher, R., et al., *Clinical course predicts long-term outcomes in bipolar disorder.* Psychological Medicine, 2019. **49**(7): p. 1109-1117. 18.       Radua, J., H. Grunze, and B.L. Amann, *Meta-Analysis of the Risk of Subsequent Mood Episodes in Bipolar Disorder.* Psychotherapy and Psychosomatics, 2017. **86**(2): p. 90-98. 19.       Ferrand, L., et al., *Which Actigraphy Dimensions Predict Longitudinal Outcomes in Bipolar Disorders?* J Clin Med, 2022. **11**(8). 20.       Ioannidis, J.P.A., *Pre-registration of mathematical models.* Mathematical Biosciences, 2022. **345**: p. 108782.

# Reviewer report: Reviewer response for version 1

**DOI:** 10.21956/wellcomeopenres.26682.r126221

**Published:** 2025-08-22

**Authors:** Abigail Ortiz

**Affiliations:** 1 University of Toronto, Toronto, Ontario, Canada

Thank you for the opportunity to review.  Hope you find my comments useful.

1. In the abstract, what do you mean by “inter-episode BD”? An episodic course of illness?
2. Add abbreviations to Table 1
3. Some typos in the text “some 65%...”
4. Methods: why did you choose 65 years as a cut off?
5. Methods: you defined euthymia using MADRS/YMRS cutoffs but not duration.  One month? Three months? Longer?
6. Methods: major concern is sample size to conduct the statistical analyses authors propose.  Increasing the sample size is an absolute requirement to ensure all this effort results in robust findings.
7. Methods: no mention of how authors will manage missing data, either clinical or actigraph.
8. Methods: no mention of safety precautions (other than specifying cutoffs for exclusion criteria using the Columbia Suicide Scale).  How will researchers manage emerging suicidality throughout the study? At some point, the authors mention “they may contact” the patient’s provider – will this be done systematically? If suicidality continues, will participants be withdrawn?
9. Methods: If those in Study 1 and 2 are going to be euthymic, there is no need to exclude those with psychosis (as you are not planning to include those with a schizoaffective d/o diagnosis, correct?)
10. Methods: why are sleep or movement d/o not an exclusion criteria for Study 1 and 2?
11. Methods: why is “current intoxication or withdrawal from illicit substances" not an exclusion criteria for Study 1 and 2?
12. Methods: See  above (#11) – does that mean that comorbid substance use or abuse is allowed for the whole sample? Please clarify.  While I am aware your intention is to have broad inclusion criteria, substance use/abuse can certainly affect sleep and other circadian parameters, even though participants may not be “acutely intoxicated”.
13. Methods: Authors talk repeatedly about “several ML”, but only mention one.  Please clarify which others you are planning to use.  If you plan to use only DBN, please justify your choice.
  1. This is particularly important for Study 2, Research Question 2: “Do any ML models enable…”.  There is no information about which models exactly you plan to use.
  2. Clarify which variables will be your input (e.g., demographic, clinical, actigraph) and what you will be your output (predicting an episode) – as there are no clear labels as to how this will be defined (MADRS score of?? YMRS score of???). 
14. Methods: Clarify why 26 weeks is your predictive horizon.  Why not shorter/larger  windows?
15. Methods: What about episodes that occur between your interviews? This is a major concern even though, rightly, the authors claim that a 13-week assessment, compared to an assessment done every 6 months, will improve the “reliability and resolution of time to relapse” (page 10).
16. Methods: Include more information about Hadaeghi’s complex system model of relapse that is easy to grasp by a clinical audience
17. Methods: See above (#16): Your audience will be clinical – explain what you mean by “trained on statistical, time-frequency, and nonlinear features”.
18. Methods: Clarify which nonlinear features are you planning to use.  There are many.
19. Methods: Treatment response comes as a surprise in Study 3.  Authors never mentioned in the Introduction their plans to study this variable.  I also find it poorly defined.  Please enumerate scales you plan to use to measure treatment response; how will you account for polypharmacy in the model; how will you ensure treatment compliance?
  1. This part is very confusing – I assume it is an output but there is a gap: what will you be measuring that will permit treatment response to be understood as a state transition?
20. Methods: Secondary Aim of Study 3 is unclear.  Please clarify.
21. More detail about the integrated algorithm for risk relapse is needed
22. Fig 3 is different from what is written: will assessments be done weekly, as per Figure; or at week 8, as per text?
23. Why are you only considering residual depressive symptoms as predictors and not residual anxiety or residual cognitive impairment – as these are also common clinical characteristics that indicate a potentially better treatment response to anticonvulsants or antipsychotics?
  1. Please justify your choice of these 5 clinical predictors

Is the study design appropriate for the research question?

Yes

Is the rationale for, and objectives of, the study clearly described?

Yes

Are sufficient details of the methods provided to allow replication by others?

No

Are the datasets clearly presented in a useable and accessible format?

Not applicable

Reviewer Expertise:

Mood disorders; nonlinear analyses; wearable technology

I confirm that I have read this submission and believe that I have an appropriate level of expertise to confirm that it is of an acceptable scientific standard, however I have significant reservations, as outlined above.

# Response

**Published:** 2026-02-03

**Authors:** GREG MURRAY

**Affiliations:** Centre for Mental Health and Brain Sciences, Swinburne University of Technology, Hawthorn, Victoria, Australia

**Reviewer’s comment** **Our response** **Changes to text** **REVIEWER 1**

1. In the abstract, what do you mean by “interepisode BD”? An episodic course of illness?

‘Inter-episode’ is a term describing people with a diagnosis of BD who do not currently meet criteria for an episode of hypomania, mania or depression [see, e.g., 1, 2]. Inter-episode is not synonymous with ‘euthymic’, which can imply an absence of residual symptoms [e.g., 3] or the presence of mood stability for a period of time [4].   We notice however that the more common spelling might be ‘interepisode’, so we have removed the hyphen throughout. ‘inter-episode’ replaced with ‘interepisode’ throughout

1. Add abbreviations to Table 1

Table 1 has been removed from Version 2 of the manuscript. We thank the reviewer for highlighting the unclear project rational in the original manuscript, and removing Table 1 improves simplicity and clarity of Version 2.   See also response to comments #11 and #18 below.   -

1. Some typos in the text “some 65%...”

We thank the reviewer for this feedback. A thorough proof-reading, with all authors checking Version 2. Several typographical errors have been corrected, and simplified wording applied throughout. Several editorial changes have been made, including:

- ‘Some 65%’ changed to ‘Approximately 65%’
- ‘lower-middle-income’ changed to ‘lower-middle income’
- ‘relative amplitude’ changed to ‘Relative Amplitude’
- ‘some 60 relapses’ changed to ‘Approximately 60 relapses’

1. Methods: why did you choose 65 years as a cut off?

We are not sure what aspect of this decision the reviewer is referring to, apologies.    An upper and lower age cut-off was set to constrain between-person variance due to adolescence and later life changes in circadian and sleep function. The upper age cut-off of 65 is conventionally used to operationalise an ‘older adult’ in mental health research. Age of 65 is also a common cut-off in bipolar disorder [e.g., 5] and sleep/circadian research [e.g., 6]. For example, Kim et al found significant differences in sleep quality with age, with decreased sleep quality amongst people aged over 65 [7, see also, 8]. Age and gender will be considered for inclusion as covariates in all analyses.   See p.13 

1. Methods: you defined euthymia using MADRS/YMRS cutoffs but not duration. One month? Three months? Longer?

We thank the reviewer for raising this question.   Firstly, these are cut-offs for Study 1 and Study 2 only. In Study 3, being in an acute episode is an inclusion criterion.   We have changed the structure of the section on inclusion/exclusion criteria to clearly separate Study 3 from Studies 1 and 2 (which largely overlap in terms of design).   In Study 1 and 2, we do not require participants to be euthymic. Participants are required to be not experiencing an episode of bipolar disorder at baseline (because we are predicting relapse into an episode). Presence of an episode is quantified for these purposes using common cutoffs of > 12 on the MADRS or > 8 on the YMRS [e.g., 9, 10].   The reviewer is correct that some bipolar disorder studies do select participants on the basis of *mood stability*, sometimes operationalised as a low level of symptoms for an extended period [e.g., 4]. However, the focus of Study 1 and 2 is people with bipolar disorder who currently do not meet episode criteria. See pp. 14-15; Footnote 3

1. Methods: major concern is sample size to conduct the statistical analyses authors propose. Increasing the sample size is an absolute requirement to ensure all this effort results in robust findings.

We thank the reviewer for raising this concern.  A paragraph of the Discussion is now devoted to consideration of this issue.   The plan to address this limitation of the project is, (i) to acknowledge in advance the implications of sample sizes for the type of conclusions that can be drawn, (ii) to plan to access open data sets containing comparable prospective actigraphy (or commercial activity measure) data amongst interepisode people with bipolar disorder. To concretize the fact that sample sizes are insufficient to generate trustworthy models, we have expanded the text about our plans to cross-validate our models in data currently being collected by other groups (with whom our team has formal connections).   The selected sample size of 100 people with BD in Study 1, 50 in Study 2 (25 BD, 25 matched controls), and 45 in Study 3 (15 manic and 30 depressed) were pragmatic maxima. We will continue to recruit beyond these numbers if funding permits.   A power analysis on the Study 1 sample is reported to quantify our chances of demonstrating a significant effect in the Cox’s survival analysis and determined that ‘even with a 20% dropout the study is nearly sufficiently powered to detect the expected relative risk of relapse (Hazard Ratio = 1.57) and well powered to detect a slightly higher relative risk of relapse (Hazard Ratio = 1.65).   See p.26 and pp. 38-39  

1. Methods: no mention of how authors will manage missing data, either clinical or actigraph.

We are happy to provide more detail on this. A new subsection has been added to Modelling approaches section.   See pp. 25-26

1. Methods: no mention of safety precautions How will researchers manage emerging suicidality throughout the study?

We thank the reviewer for raising risk management and are happy to provide more detail.   Note that only Study 1 and 2 are under consideration here. In Study 3, all participants are recruited from either inpatient or outpatient settings of a specialist mental health team in Christchurch, New Zealand. Study 3 participants remain under the care of this service, and existing risk management procedures will apply.   Key principles of the approach include protection of autonomy where possible, explicit assignment of risk management to participants and their local networks, and advance planning for crises [11]. Text edited and expanded in *Risk Management Approach. *The new text more clearly distinguishes between management processes for *acute suicidality* versus management of a participant in whom suicidality or other clinical risk issues emerge through their involvement.   See pp.31-34

1. Methods: If those in Study 1 and 2 are going to be euthymic, there is no need to exclude those with psychosis

Agreed. By definition, a person cannot meet eligibility criteria if they are psychotic outside an episode or are currently in an episode. Reference to acute psychosis exclusion removed.

1. Methods: why are sleep or movement d/o not an exclusion criterion for Study 1 and 2?

The reviewer identifies two issues that were not adequately explained in the text, (i) the role of assessment of sleep disorders and other comorbidities, and (ii) exclusion criteria based on potential confounding of the actigraphy signal.   Having a physical problem that might confound RAR interpretation is an exclusion in all three studies and is a common exclusion in actigraphy research. The confusion in our text was that *different examples of physical problems that might impact RAR interpretation were provided for Study 1 and 2 compared with Study 3*. Text has been revised and some new text added.   Presence of common BD comorbidities is not an exclusion criterion. Anxiety disorders, substance use disorders and sleep disorders will be assessed by structured clinical interview at baseline and explored for potential inclusion in predictive models. This approach allows us to build models that generalise to complex clinical presentations while still accounting for their impact on models.   See last para p.13; first para p.14 11. Methods: why is “current intoxication or withdrawal from illicit substances" not an exclusion criteria for Study 1 and 2? We thank the reviewer for picking up this ambiguity. The reviewer has highlighted that the overall logic of the multi-study design can be made clearer.   The exclusion for "current intoxication or withdrawal" is only relevant in Study 3, in which participants are primarily recruited from an inpatient setting. It is not uncommon for individuals to be admitted to hospital in a state of intoxication or acute withdrawal (e.g., from alcohol or methamphetamines). These states would profoundly impact the capacity to consent and confound the actigraphy data collected in the initial days. Therefore, as a standard procedural step in this inpatient setting, potential participants are only approached for recruitment after this acute phase (~72 hours) has passed. Text signposting the project- versus study-level differences has been added, as has a new Box which compares and contrasts the three studies’ methods at a glance.   See first para. p15; Box 1 on p.45      

1. Methods: See above (#10) – does that mean that comorbid substance use or abuse is allowed for the whole sample?

We thank the reviewer for this request for clarification about the measurement of comorbid diagnoses. See response to point 11 above. Additional text added to Inclusion and Exclusion criteria Studies 1 and 2, new Box added.   The two most prevalent psychiatric comorbidities of BD (anxiety and substance disorders), and comorbid sleep disorders are rigorously assessed by clinical interview in all three studies. Comorbid substance use/abuse within the last 12 months (measured on the SCID for those who screen as potentially impacted by self-report AUDIT and DUDIT), anxiety disorders (measured on the SCID) and sleep disorders (via SCISD-R). To maximise generalisability of the findings, presence of these disorders is not an exclusion criterion.   The reviewer is correct that these comorbidities can impact sleep and circadian function. Anxiety, substance and sleep disorders will be investigated as potential covariates in analyses. See first para, p.14        

1.  Methods: Authors talk repeatedly about “several ML”, but only mention one.

  13.1 This is particularly important for Study 2, Research Question 2: “Do any ML models enable…”. We thank the reviewer for this observation. The text has been expanded to clarify that multiple complementary machine learning (ML) methods will be employed, each addressing a distinct analytical aim.       See p.25, pp.27-28

   13.2 Clarify which variables will be your input (e.g., demographic, clinical, actigraph) and what you will be your output (predicting an episode) We agree that this could have been made clearer in the original MS.  In Version 2, several changes have been made, including a new subsection headed ‘ **Input and outcome variables’**     See pp.30-31; p.24-25

1. Methods: Clarify why 26 weeks is your predictive horizon. Why not shorter/larger windows?

We thank the reviewer for requesting this clarification about Study 1 and 2 timeframes.   The dynamics of bipolar disorder course have been investigated across timescales from days [emotion regulation, e.g., 12] to weeks [mood instability, rapid cycling, 13, 14] to years [predominant polarity, seasonality, lifetime course, 15].  Research aiming to predict relapse events has tended to use longer predictive horizons [e.g., 3 years, 16]. The total predictive horizon for participants in Study 1 here is 78 weeks, assessed in six 13-week assessment windows (i.e., 3 X 26 week epochs, with two assessment interviews per epoch).   As mentioned in the first paragraph of the Introduction, the focus here is on “improving forecasting of the short-term course’ (weeks and months)”. This a clinically important timeframe:  the algorithm identified here could form part of a risk calculator for clinicians and patients.   Text clarified – brief rationale for 26-week predictive horizon added.   See p.16

1. Methods: What about episodes that occur between your interviews?

We thank the reviewer for highlighting a central design feature that we did not make clear. New text has been added in Version 2 (see also #5 above), along with further explication of the LIFE.  

1. The primary outcome in Study 1 and 2 is measured on the *retrospective* LIFE interview conducted at 13 and 26 weeks per epoch.
2. The primary purpose of the LIFE interview is to provide a medium-term measure of mood states across time, avoiding the participant burden of *prospective self-monitoring *of symptoms, and the problem that increasing symptoms are associated with growing impairment of insight.
3. As described in the section ***Primary outcome measures, *** the LIFE generates a weekly mood score, providing an estimate of presence versus absence of an episode per week in the retrospective window.
4. The reviewer highlights that there could be some confusion around the fact that Study 1 and Study 2 employ two very different measures of presence of an episode (MADRS/YMRS cut-offs versus LIFE retrospective estimates of weekly episode status). We have added a footnote to address this ambiguity.

See pp.22-23, including Footnote 5; see Footnote 4 on p.14.

1. Methods: Include more information about Hadaeghi’s complex system model of relapse

We thank the reviewer for these insightful comments (15, 16,17). The Complex systems analysis subsection has been rewritten to improve accessibility for a clinical readership. It has also been expanded to clarify the model inputs and nonlinear features to be examined.   We thank the reviewer for this suggestion.  New text has been added to explain Hadaeghi’s Complex System Model. This model conceptualises bipolar disorder (BD) as a dynamic system with two interacting subsystems: the circadian pacemaker (sleep–wake regulation) and the frontal–limbic emotional regulation network. Disturbances in either subsystem, or in their feedback loops, can push the system toward a critical transition or “tipping point,” corresponding clinically to a relapse into mania or depression. The new material includes explicit statement about the types of nonlinear features that are likely to appear in analyses.      See pp.28-30

1. Methods: See above (#16): Your audience will be clinical – explain what you mean by “trained on statistical, time-frequency, and nonlinear features”.

1. Methods: Clarify which nonlinear features are you planning to use. There are many.

1. Methods: Treatment response comes as a surprise in Study 3. 

    We thank the reviewer for requesting more detail on this issue. The original manuscript does include some words about the role of Study 3, but this comment has encouraged us to conduct a root and branch review of this aspect of the project.       From a complex systems viewpoint, relapse into a new episode is a critical state transition – metaphorically a tipping point. Our primary clinical objective in is to develop a predictive algorithm for this transition. To ensure this algorithm is robust and mechanistically informed, however, our project design includes a complementary investigation of an alternative state transition, namely, recovery from an acute episode. *Studying this second transition is not the primary clinical goal but is scientifically important. It allows us to test whether the signals predicting relapse are markers of state transition in general, thereby increasing confidence in their validity. It also provides a unique window into the system's dynamics during a period of rapid change, offering mechanistic insights that may refine the primary relapse model.* New text has been added at multiple points, and Abstract rewritten to elevate the notion of state transition in the project logic. Text introducing the project at the end of the Introduction has been revised to highlight the mechanistic significance of investigating whether dynamic models predicting relapse (from Study 1) extend to prediction of recovery (Study 3).   See Abstract; p.7; pp.11-12; pp.18-19; p.38; revised Figure 3.         Reviewer comment 18 continued:

1. Please enumerate scales you plan to use to measure treatment response;
2. How will you account for polypharmacy in the model;
3. How will you ensure treatment compliance?

We agree that the description of measurement of response to treatment in Study 3 was ambiguous in text and Figure 3.  Figure 3 has now been redrawn to focus only on the primary aim of Study 3.       Study 3 has primary, secondary, and tertiary aims. The primary aim is to investigate the extension of the algorithm informed by Hadaeghi’s Complex System Model of relapse prediction (trained and tested on Study 1 data) to response to treatment, with both relapse (Study 1 and 2 outcome) and treatment response (Study 3 outcome) being understood as state transitions (Hadaeghi et al., 2016).   The state transition of Study 3 is measured in a binary variable, namely, response to treatment operationalised as 50% reduction in MADRS (for those in depressive episode at baseline) or 50% reduction in YMRS (for those in manic episode at baseline) at 8 weeks. Participants will wear actigraphs for 21 days – for consistency with Study 1 and 2, the first 14 days only will be used in modelling of binary response to treatment.   Study 3 is a naturalistic study of treatment in the real world, and important covariates can be measured but not controlled.  Polypharmacy will be drawn from hospital records. Treatment compliance cannot be ensured, but we will record any notes about treatment compliance from hospital records for consideration.      See updated p.18 and Figure 3 19.1 This part is very confusing – I assume it is an output but there is a gap: what will you be measuring that will permit treatment response to be understood as a state transition? Agree. See new text added in response to #13.2 above

1. Methods: Secondary Aim of Study 3 is unclear. Please clarify.

Agree. Wording of secondary aim clarified, with primary, secondary and tertiary aims of Study 3 now distinguished. Text corrected throughout and Figure 3 redrawn to accurate reflect Study 3’s primary aim, and the primary aim only.        See pp.18-19; new Figure 3.

1. More detail about the integrated algorithm for risk relapse is needed

We are happy to provide more detail. **Work Package 4: Integrative Data Synthesis** section has been rewritten and expanded.    It is important to note that it is not possible to predict in advance what specific variables will warrant inclusion in models – this will depend on the distributions of collected variables and their interrelationships [20]. The project is therefore exploratory.  The Editor of Wellcome Open has agreed that we will submit a detailed data analysis plan only after data has been collected, screened and cleaned. See p.20-21

1. Fig 3 is different from what is written: will assessments be done weekly, as per Figure; or at week 8, as per text?

We apologise for this ambiguity, which has been corrected. Figure 3 has been redrawn, and text corrected.  See response to #19 above.

1. Why are you only considering residual depressive symptoms as predictors and not residual anxiety or residual cognitive impairment – as these are also common clinical characteristics that indicate a potentially better treatment response to anticonvulsants or antipsychotics?

We thank the reviewer for picking up on this ambiguity across studies.  The clinical predictors we are modelling are described for Study 1 and 2 only, and Version 2 makes this clear.  Text has been clarified to ensure the reader knows which studies include the five clinical predictors.   We are not planning to add clinical predictors into the models of Study 3 given the small sample size, and concerns about participant burden. Note that, as the reviewer comments, candidate predictors of response to treatment are not the same as candidate predictors of relapse.   See our response to #23.1 below for further discussion of our choice of five specific ‘clinical predictors’ to include in models of Study 1 and 2. See pp.24-25 23.1 Please justify your choice of these 5 clinical predictors These five clinical predictors are only being used in Studies 1 and 2, not the Study 3 prediction of treatment response (these are evidence-based predictors of relapse, not response to treatment). The five chosen predictors have now been explicitly justified in new text.   Given the focus here on modelling analyses, we cannot predict in advance what specific variables will warrant inclusion in models [20]. The project is exploratory, and the *Editor of Wellcome Open has accepted our suggestion that we will submit a detailed data analysis plan only after we have collected, screened and cleaned data*. See p.24-25

1. Is the rationale for, and objectives of, the study clearly described? YES

- -

1. Is the study design appropriate for the research question? YES

- -

1. Are sufficient details of the methods provided to allow replication by others? NO

We agree that the manuscript provides insufficient details for replications. We had mentioned in Footnote 1 that methodological details of each study would be provided in separate study-level protocol papers, but we agree with the reviewer that this manuscript needs to be more explicit about the relationship between project-level and study-level decisions.   Footnote 1 has been removed, and the explanation for the project-level protocol paper moved to text and expanded in an intro paragraph to the Protocol section   See p.12 and Box 1 **References** 1.         Gershon, A., et al., *Daily Actigraphy Profiles Distinguish Depressive and Interepisode States in Bipolar Disorder.* Clin Psychol Sci, 2016. **4**(4): p. 641-650. 2.         Ng, T.H., et al., *Sleep–wake disturbance in interepisode bipolar disorder and high-risk individuals: A systematic review and meta-analysis.* Sleep Medicine Reviews, 2015. **20**: p. 46-58. 3.         Fujita, A., et al., *Comparison of objective and subjective sleep parameters in patients with bipolar disorder in both euthymic and residual symptomatic periods.* J Psychiatr Res, 2021. **145**: p. 190-196. 4.         Yeom, J.W., et al., *Daily Self-Monitoring and Feedback of Circadian Rhythm Measures in Major Depression and Bipolar Disorder Using Wearable Devices and Smartphones-The Circadian Rhythm for Mood (CRM®) Trial Protocol: A Randomized Sham Controlled Double-Blind Trial.* Psychiatry Investig, 2024. **21**(8): p. 918-924. 5.         Murray, G., et al., *Online mindfulness-based intervention for late-stage bipolar disorder: pilot evidence for feasibility and effectiveness.* J Affect Disord, 2015. **178**: p. 46-51. 6.         Kahawage, P., et al., *Social Rhythm Disruption is Associated with Greater Depressive Symptoms in People with Mood Disorders: Findings from a Multinational Online Survey During COVID-19.* Can J Psychiatry, 2022. **67**(11): p. 831-840. 7.         Kim, M., et al., *Association Between Age and Sleep Quality: Findings From a Community Health Survey.* Sleep Med Res, 2021. **12**(2): p. 155-160. 8.         Hood, S. and S. Amir, *The aging clock: circadian rhythms and later life.* J Clin Invest, 2017. **127**(2): p. 437-446. 9.         Earley, W., et al., *Clinically relevant response and remission outcomes in cariprazine-treated patients with bipolar I disorder.* J Affect Disord, 2018. **226**: p. 239-244. 10.       Porter, R.J., et al., *Validation of the Longitudinal Interval Follow-Up Evaluation for the Long-Term Measurement of Mood Symptoms in Bipolar Disorder.* Brain Sciences, 2022. **12**(12): p. 1717. 11.       Fletcher, K., et al., *Web-based intervention to improve quality of life in late stage bipolar disorder (ORBIT): Randomised controlled trial protocol.* BMC Psychiatry, 2018. **18**(1): p. 221. 12.       Kim, J.A., et al., *Chronotherapeutic intervention targeting emotion regulation brain circuitry, symptoms, and suicide risk in adolescents and young adults with bipolar disorder: a pilot randomised trial.* BMJ Ment Health, 2025. **28**(1). 13.       Stromberg, A.R., et al., *Modeling and predicting mood instability in a longitudinal cohort of bipolar disorder.* Nature Mental Health, 2025. **3**(10): p. 1267-1275. 14.       Schneck, C.D., et al., *The prospective course of rapid-cycling bipolar disorder: findings from the STEP-BD.* Am J Psychiatry, 2008. **165**(3): p. 370-7; quiz 410. 15.       Fiedorowicz, J.G., et al., *Validation of the youth mood recurrences risk calculator in an adult sample with bipolar disorder.* J Affect Disord, 2021. **295**: p. 1482-1488. 16.       Etain, B., et al., *Clinical predictors of recurrences in bipolar disorders type 1 and 2: A FACE-BD longitudinal study.* J Psychiatr Res, 2021. **134**: p. 129-137. 17.       Uher, R., et al., *Clinical course predicts long-term outcomes in bipolar disorder.* Psychological Medicine, 2019. **49**(7): p. 1109-1117. 18.       Radua, J., H. Grunze, and B.L. Amann, *Meta-Analysis of the Risk of Subsequent Mood Episodes in Bipolar Disorder.* Psychotherapy and Psychosomatics, 2017. **86**(2): p. 90-98. 19.       Ferrand, L., et al., *Which Actigraphy Dimensions Predict Longitudinal Outcomes in Bipolar Disorders?* J Clin Med, 2022. **11**(8). 20.       Ioannidis, J.P.A., *Pre-registration of mathematical models.* Mathematical Biosciences, 2022. **345**: p. 108782.
